# Supplementary material for: Design of Tissue-Selective PROTACs Through Recruiting E3 Ligase Scaffolding Protein MAGEA11
Source: bioRxiv. 2025 Nov 3:2025.11.03.686228. Preprint. [Version 1] doi: 10.1101/2025.11.03.686228 (PMC12637679; doi:10.1101/2025.11.03.686228)
Supplement: Supplement 1 [file media-1.pdf]

Supporting Information

Design of Tissue-Selective PROTACs Through Recruiting E3 Ligase Scaffolding Protein MAGEA11

Isabella E. Jacobsen[1], Rui Shi[2], Cole R. Scholtz[1], William C.K. Pomerantz[1,2] and Gunda I. Georg[1,2]

[1]Department of Chemistry, University of Minnesota, 207 Pleasant St SE, Minneapolis, MN 55455, USA

[2]Department of Medicinal Chemistry and Institute for Therapeutics Discovery and Development, College of Pharmacy, University of Minnesota, 717 Delaware Street SE, Minneapolis, MN 55414, USA

Table of Contents

Biological Procedures 3
Supplementary Figures (S1-S13) 5
Supplementary Figure S1 5
Supplementary Figure S2 6
Supplementary Figure S3 7
Supplementary Figure S4 7
Supplementary Figure S5 8
Supplementary Figure S6 8
Supplementary Figure S7 8
Supplementary Figure S8 9
Supplementary Figure S9 10
Supplementary Figure S10 10
Supplementary Figure S11 11
Supplementary Figure S12 11

Supplementary Figure S13 12
Instrumentation and Materials 12
Synthetic Route and Characterization Data for PROTACs and SJ1008066 13
Supplementary Scheme 1: 13
Supplementary Scheme 2: 18
References 31
NMR Spectra 32
HPLC 44
HRMS/LCMS 60

Biological Procedures

Materials and Equipment

(+)-JQ1, MZ1, MG132, and MLN4924 were purchased as lyophilized powders from MedChem Express.

Cell Culture

All cell lines were cultured in a humidified environment at 37 °C with 5% CO_2. U2OS cells (ATCC HTB-96) were cultured in McCoy's 5A Media (ATCC 30-2007) with 5% 100 μg/mL penicillin-streptomycin solution and 10% fetal bovine serum. KYSE180 cells were cultured in RPMI 1640 media (ATCC 30-2001) with 5% 100 μg/mL penicillin-streptomycin solution and 10% fetal bovine serum. HEK293T cells (ATCC CRL-2316) were grown in DMEM (ATCC 30-2002) with 5% 100 μg/mL penicillin-streptomycin solution and 10% fetal bovine serum. For treatment, cells were plated in 6-well plates (Corning Costar 3516) at a density of 200,000 cells per well and allowed to adhere overnight. The compounds to be tested were dissolved in DMSO to a concentration of 10 mM and further diluted in media to achieve a final concentration of 1% DMSO. A 10x solution of the compounds was added to the cell plates at the desired concentration, and the cells were incubated for the desired time. The media was then removed, and the cells were washed with 1 mL of cold PBS. The cells were lysed with RIPA buffer (Sigma Aldrich) supplemented with cOmplete mini protease inhibitor cocktail (Roche, 1 tablet in 10 mL RIPA).

Western Blot

The soluble and insoluble layers of cellular lysates were separated via centrifugation (15000 rpm, 4 °C, 15 min). The soluble fraction was then removed, and the total protein concentration of each lysate was determined via a BCA assay kit (Thermo Fischer). The total protein levels of each lysate were then normalized by dilution with RIPA buffer, and 4x NuPAGE LDS sample buffer (Invitrogen) was added to each sample. The samples were boiled for 5 minutes, and the proteins were separated on a NuPAGE 3-8% tris-acetate gel (Invitrogen). The proteins were transferred to a PVDF membrane (BioRad) using a BioRad semi-dry Turbo Blot transfer system for 10 minutes, blocked with 5% milk in tris-buffered saline with 0.1% Tween 20 (TBST) for 1 h, and incubated with the appropriate antibody at 4 °C for 16 h. The membranes were then washed with TBST (10 mL, 5 x 3 min), incubated with the appropriate secondary antibody for 1 h, and washed with TBST (10 mL, 5 x 3 min). The proteins were visualized with SuperSignal West substrate (either Pico or Femto -Thermo Fischer) and a Licor Omega or BioRad ChemiTouch imaging system. Protein bands were quantified with ImageJ software and normalized to the appropriate loading control. Select blots were stripped with Restore™ PLUS Western Blot Stripping Buffer (Thermo Scientific) according to the manufacturer's protocol.

Protein Target	Species	Manufacturer (product #)	Dilution	Substrate
BRD4	Rabbit	Cell Signaling Technology (E2A7X)	1:1000	Pico
BRD2	Rabbit	Cell Signaling (D89B4)	1:1000	Femto
BRD3	Rabbit	Bethyl (BLR069G)	1:1000	Femto
BRDT	Rabbit	Cell Signaling (E6T6E)	1:1000	Pico
c-Myc	Rabbit	Cell Signaling (D84C12)	1:1000	Femto
RUNX2	Rabbit	Cell Signaling (D1H7)	1:1000	Femto
KRT14	Rabbit	Cell Signaling (46251S)	1:1000	Pico
MAGEA11	Rabbit	Cell Signaling (E2F1K)	1:1000	Femto
Tubulin	Rabbit	Cell Signaling (2148)	1:2000	Pico
Actin	Mouse	Millipore Sigma (A3853)	1:2000	Pico
Vinculin	Mouse	Invitrogen (14-9777-82)	1:1000	Femto
Mouse- HRP Conjugate	Goat	Invitrogen (31430)	1:2000	NA
Rabbit- HRP Conjugate	Goat	Invitrogen (31460)	1:2000	NA

Fluorescence Polarization

A his-tagged BRD4-BD1 was recombinantly expressed (residues 44-168), and a fluorescence polarization assay was performed as previously described.[1]

Cell Viability

Cells were seeded in a 384-well white opaque plate (Corning 3570) at a density of 500 cells/well in 25 µl total volume and allowed to adhere overnight. To each well was added a 10x stock of the desired compound at the desired concentration and titration, and the cells were incubated with the compound for 72 h. Viability was measured with CellTiter-Glo® 2.0 Cell Viability Assay (Promega) as follows: The cell plates and CellTiter-Glo® 2.0 reagent were allowed to equilibrate to room temperature for 30 minutes. CellTiter-Glo® 2.0 reagent (25 µl) was added to each well, and the plate was shaken on an orbital shaker for 2 minutes, then allowed to equilibrate undisturbed at room temperature for 10 minutes. The luminescence of each well was measured for 1 second with a Tecan Spark. The raw luminescence values were normalized to a vehicle control and plotted with GraphPad Prism 10 to calculate a GI_{50} value.

Supplementary Figures (S1-S13)

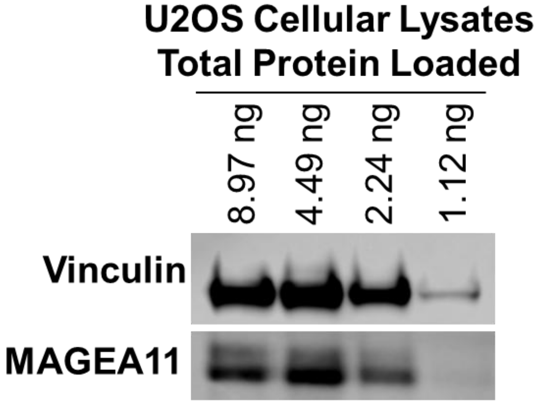

Supplementary Figure S1. Western blot confirmation of MAGEA11 expression in U2OS cells.

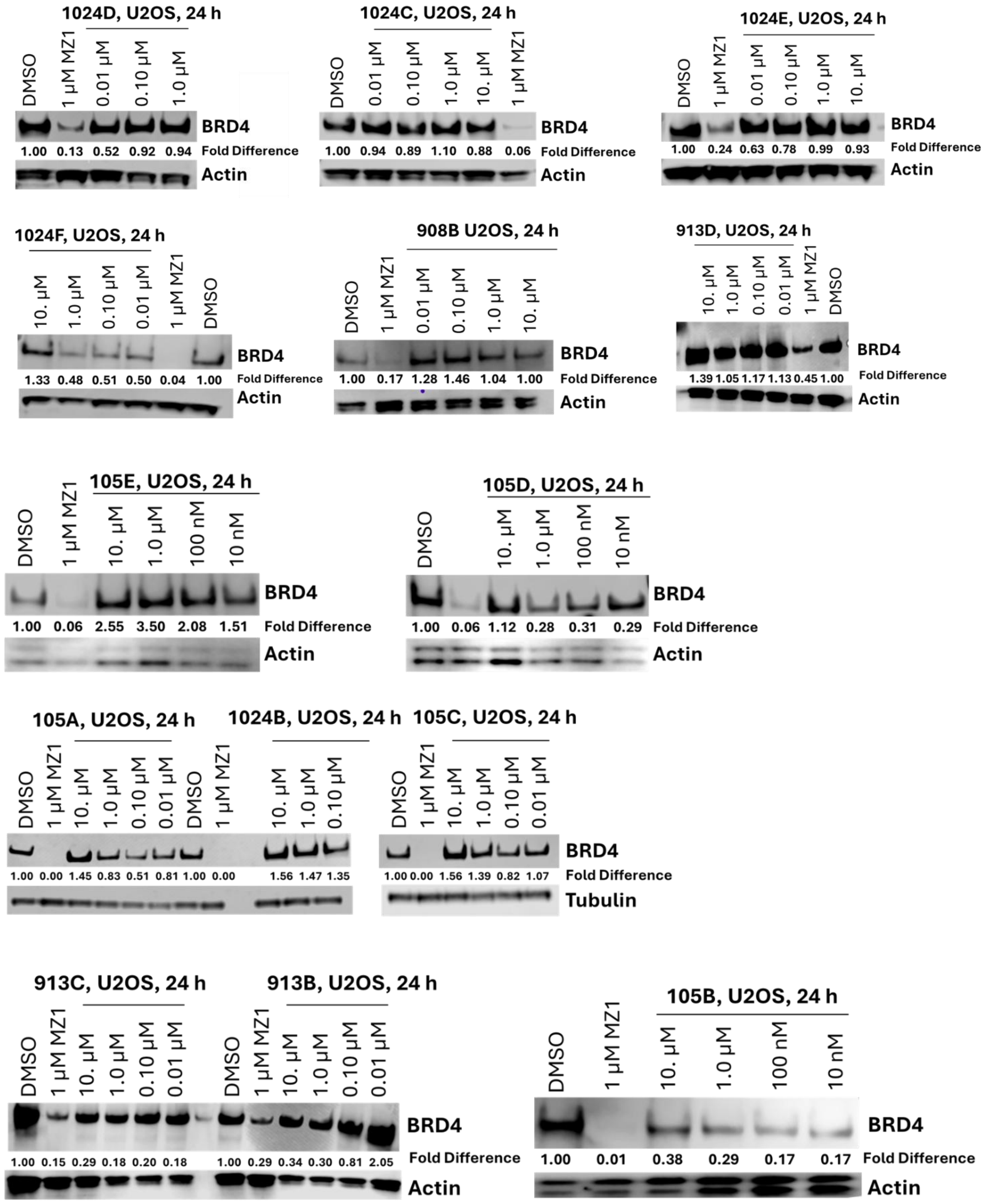

Supplementary Figure S2. Screening data for the PROTACs library.

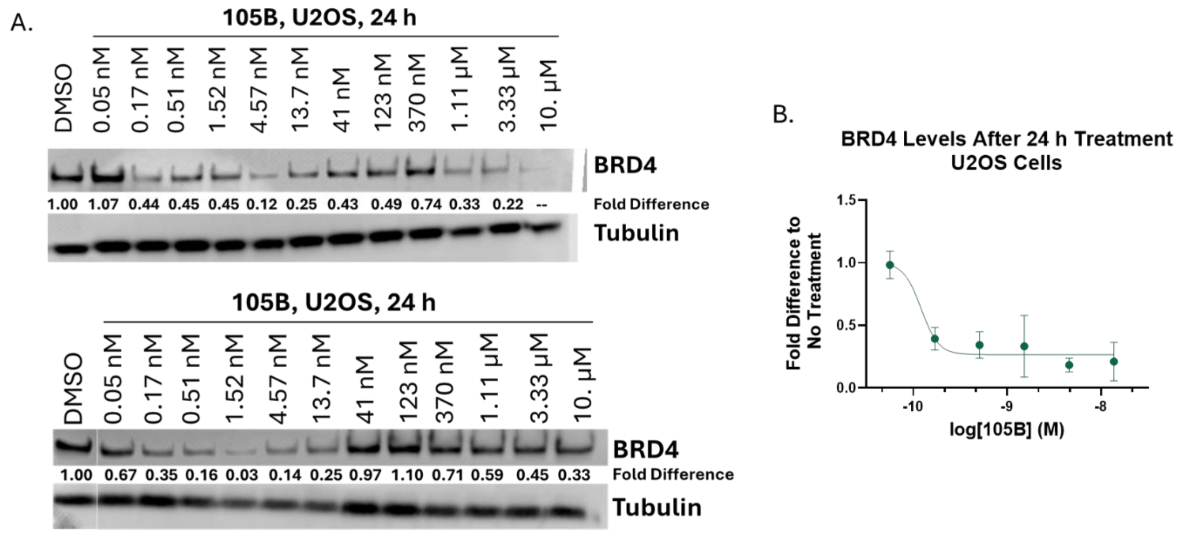

Supplementary Figure S3. Additional replicates of 12-point dose response curves of 105B in U2OS cells at 24 hours. A. Western blots of quantified BRD4 degradation. B. Dose-response curve used to calculate DC_{50} of 105B using GraphPad Prism 10. Each point represents the mean ± standard deviation of three experimental replicates. Points past the hook effect (> 41 nM) were excluded from the analysis.

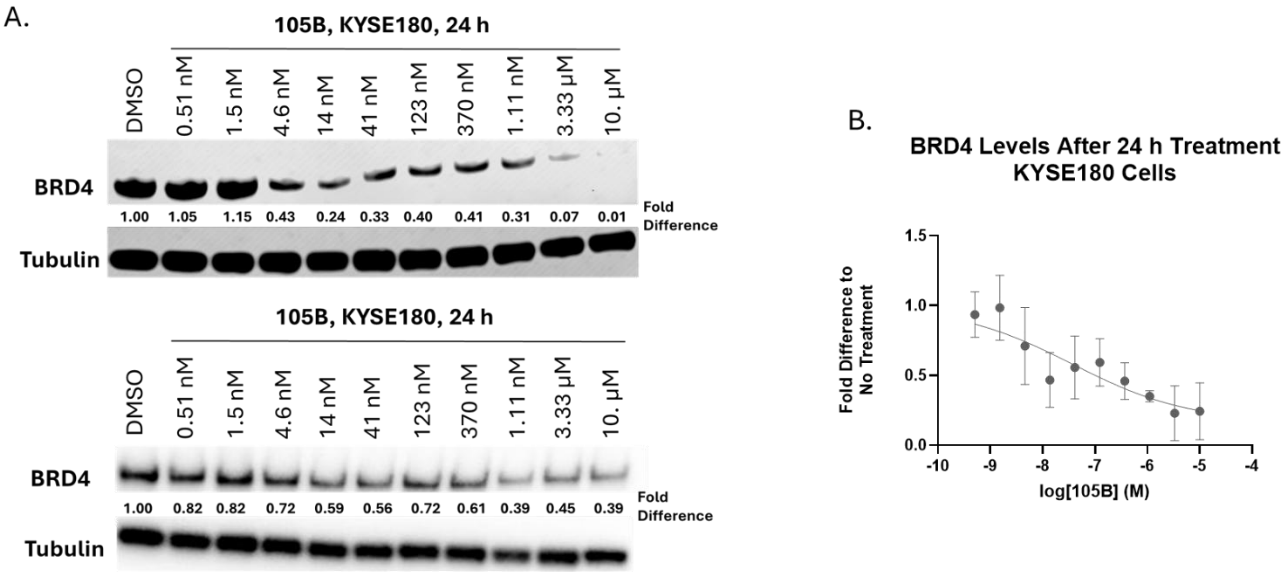

Supplementary Figure S4. Additional replicates of 12-point dose response curves of 105B in KYSE180 cells at 24 hours. A. Western blots of quantified BRD4 degradation. B. Dose-response curve used to calculate DC_{50} of 105B using GraphPad Prism 10. Each point represents the mean ± standard deviation of three experimental replicates.

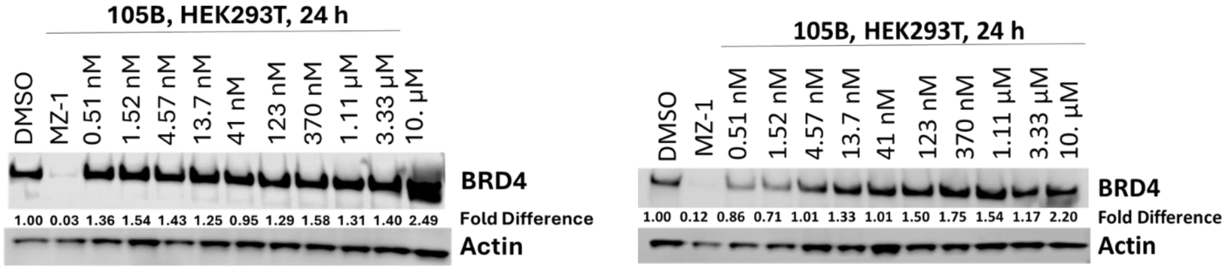

Supplementary Figure S5. Additional western blot replicates of 10-point dose response curves of 105B in HEK293T cells at 24 hours.

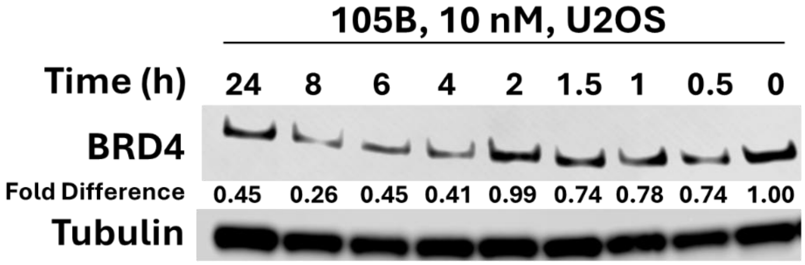

Supplementary Figure S6. Replication of the time course degradation experiment.

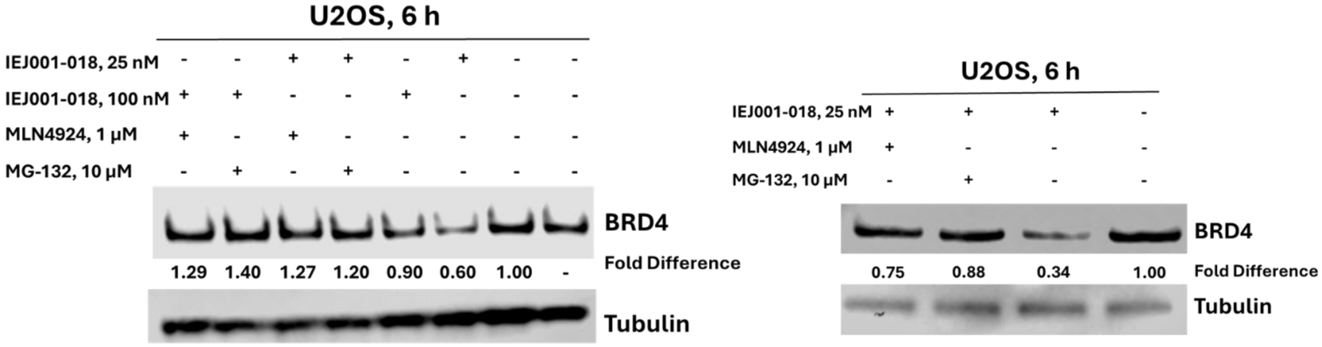

Supplementary Figure S7. Replicates of the MG132 and MLN4924 experiments.

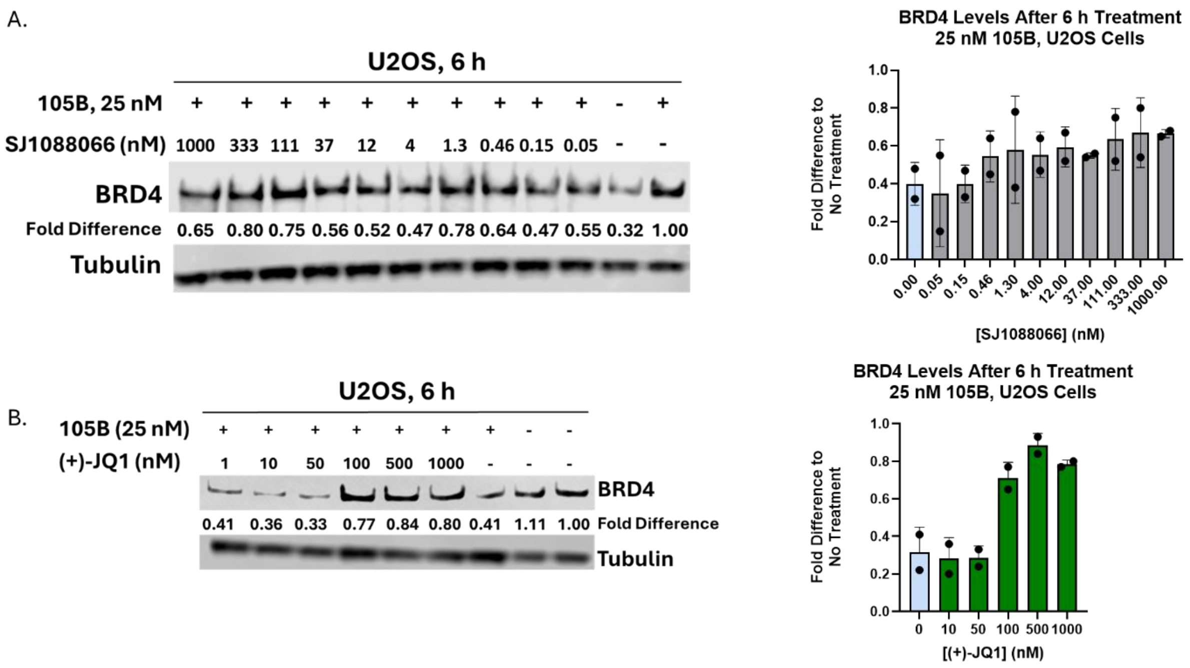

Supplementary Figure S8. Replicates of the ligand competition experiments. A. Replicate of the competition with increasing concentrations of SJ1088066. The graph represents the mean and standard deviation of two replicates. B. Replicate of competition with (+)-JQ1. The graph represents the mean and standard deviation of two replicates.

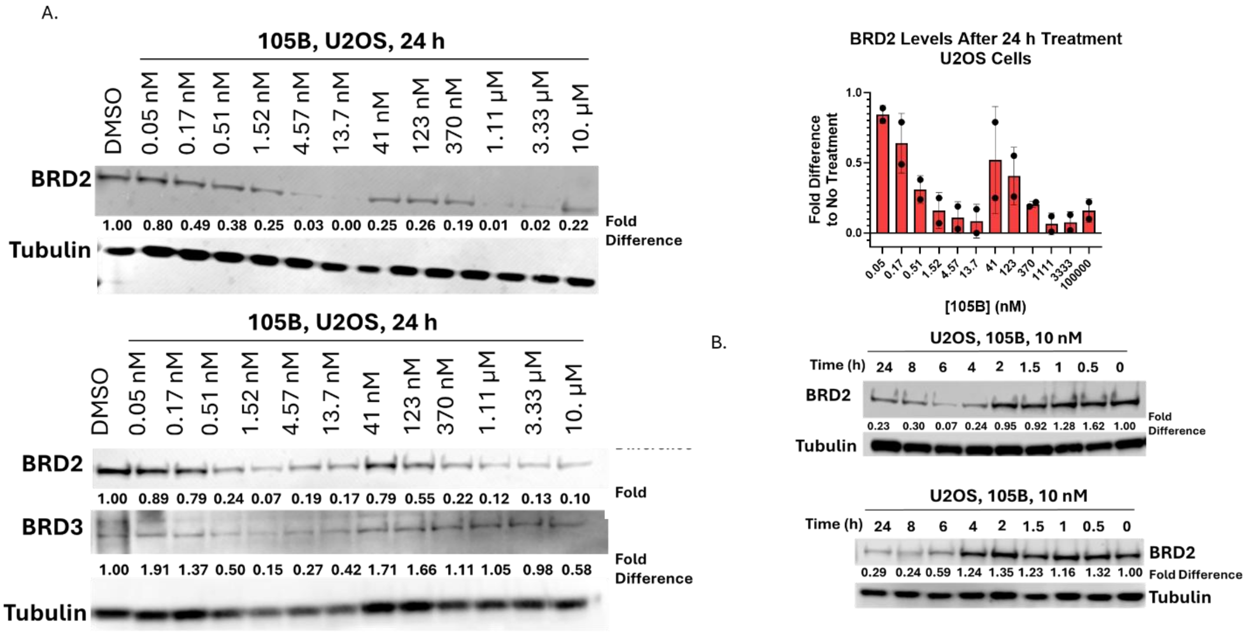

Supplementary Figure S9. Degradation of BRD2 and BRD3 in U2OS cells with 105B. A. Replicates of 12-point dose response of BRD2/3 degradation with 105B. The graph represents the mean of two replicates ± SD. B. Replicates of time course degradation of BRD2.

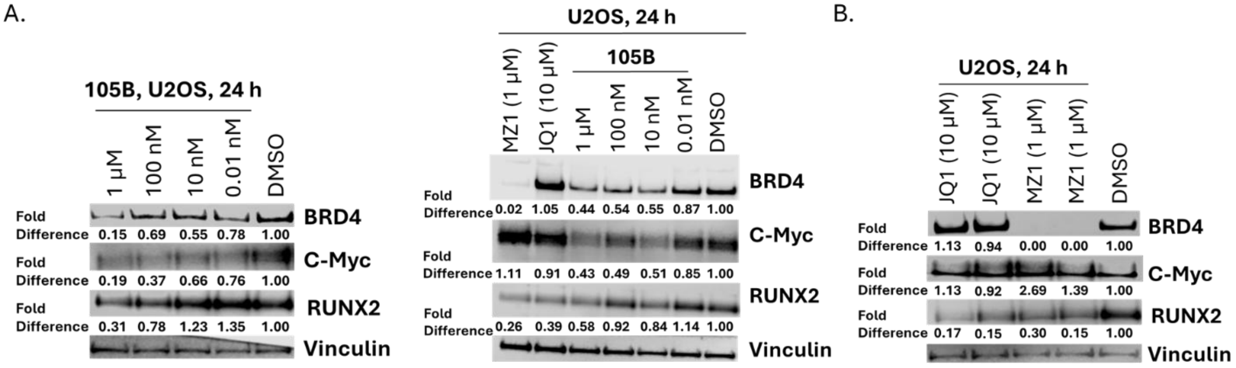

Supplementary Figure S10. Replicates of c-Myc and RUNX2 western blots in U2OS cells. A. Replicates of c-Myc and RUNX2 levels in U2OS cells after incubation with 105B for 24 hours. B. Replicates of c-Myc and RUNX2 levels in U2OS cells after incubation with controls (+)-JQ1 and MZ-1 for 24 hours.

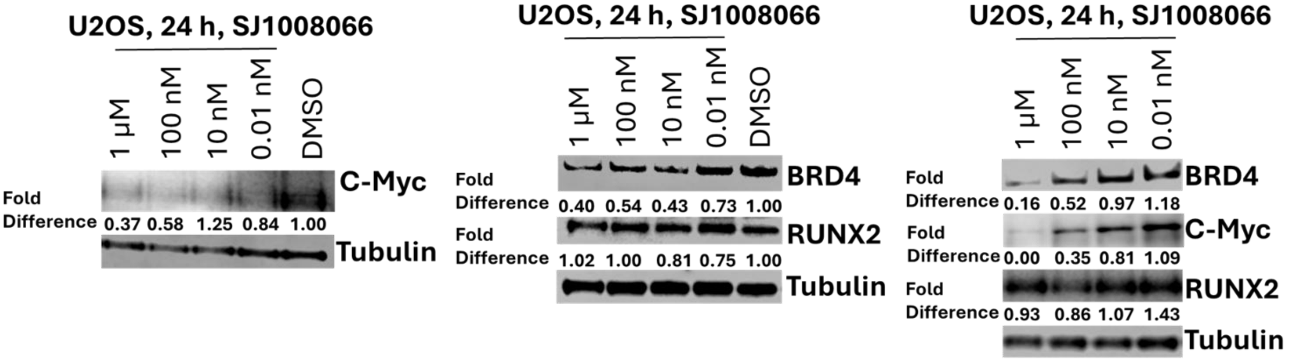

Supplementary Figure S11. Replicates of western blots probing levels of BRD4, c-Myc, and RUNX2 in U2OS cells treated with SJ1008066.

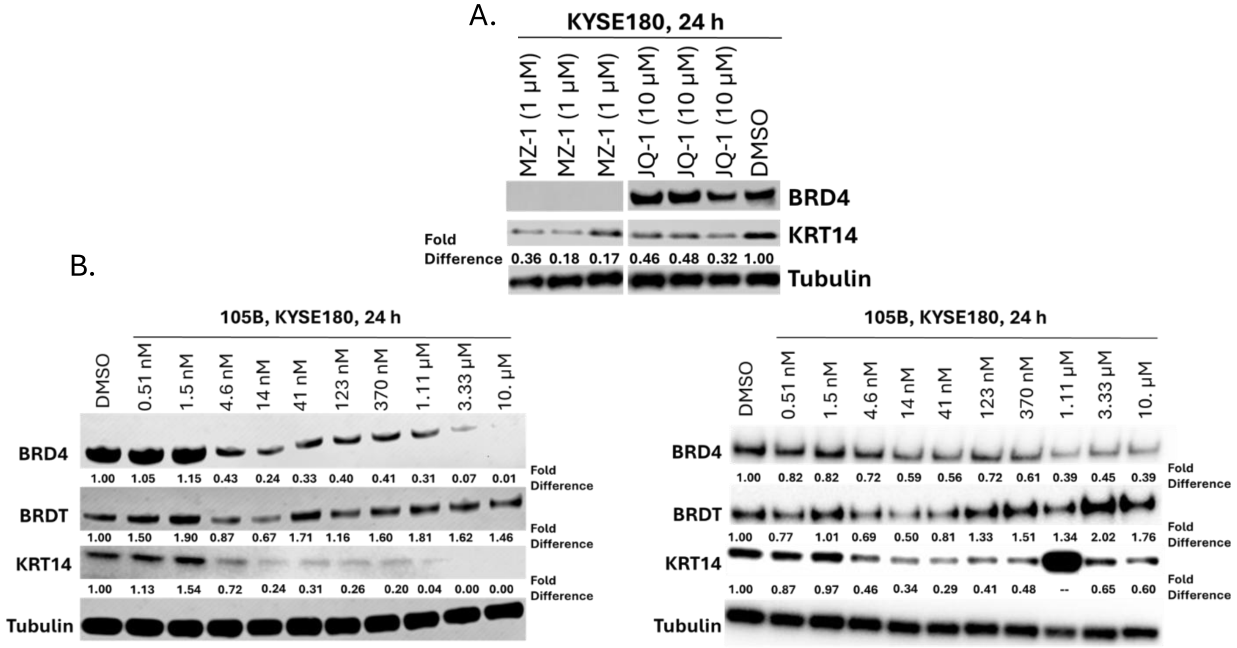

Supplementary Figure S12. A. Replicates of western blots of KRT14 and BRD4 levels in KYSE180 cells treated with (+)-JQ1 and MZ-1. B. Replicates of western blots probing levels of BRDT and KRT14 in KYSE180 cells. KRT14 levels in points 1.11 μM- 10 μM were not used in replicate 3 due to insoluble cell debris in the 1.11 μM lane, distorting the signal.

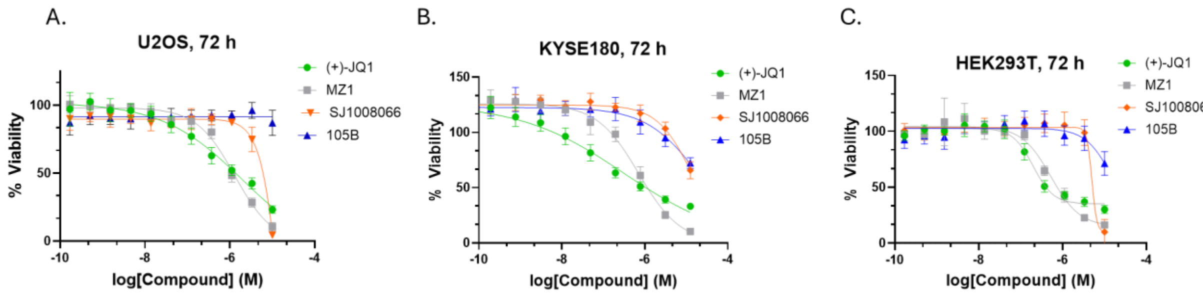

Compound	GI_{50} (μM)
(+)-JQ1	1.32 ± 0.27
MZ1	1.31 ± 0.1
SJ1008066	5.22 ± 0.39
105B	--

Compound	GI_{50} (μM)
(+)-JQ1	0.364 ± 0.147
MZ1	0.717 ± 0.070
SJ1008066	>10
105B (% viability at 10 μM)	72.3 ± 2.2

Compound	GI_{50} (μM)
(+)-JQ1	0.194 ± 0.020
MZ1	0.572 ± 0.126
SJ1008066	7.16 ± 1.85
105B (% viability at 10 μM)	71.3 ± 9.4

Supplementary Figure S13. CellTiter-Glo cell viability data for: A. U2OS cells, B. KYSE180 cells, and C. HEK293T cells. All curves represent triplicate data as the mean ± SD.

Instrumentation and Materials

Chemicals: (+)-JQ1 acid, 7-bromo-4-chloro quinoline, and (1*S*,2*S*)-*N*-*N*'-dimethylcyclohexane-1,2-diamine were purchased from Ambeed. All amine and propyl functionalized PEG linkers and indole-5-carbaldehyde were purchased from Astatech. All other chemicals, including solvents, were purchased from Sigma Aldrich. All chemicals were used from suppliers without further purification. **Safety statement:** Sodium azide poses an explosion risk if concentrated under reduced pressure.

HPLC: Purity of all compounds was obtained with a C18 column (201HS54 Vydac) on an UltiMate 3000 HPLC with a 0-50% gradient of ACN in H_2O w/0.1% TFA. 1 mM sample stocks in DMSO were injected onto HPLC and purity was calculated from the 254 nm wavelength UV trace.

NMR: NMR was measured on a Bruker Avance 700/850-MHz, Bruker Avance NEO 600-MHz, or a Bruker Avance III 400/100-MHz NMR instrument. Chemical shifts are reported in parts per million (ppm). Splitting patterns are designed as s, singlet; d, doublet; t, triplet; q, quartet; m, multiplet; br, broad singlet. Chemical shifts are reported in ppm relative to C_2D_6SO (2.50 ppm for ^{1}H, 39.5 ppm for ^{13}C). NMR spectra were processed with the MestReNova or TopSpin programs.

HRMS: HRMS was measured on a Sciex X500R QTOF-MS in MeOH.

LCMS: Characterization with LCMS was done with an Agilent 1260 Infinity II instrument with a 3x150 mm C18 column with a 5-100% gradient of ACN in H_2O w/0.1% formic acid.

Synthetic Route and Characterization Data for PROTACs and SJ1008066

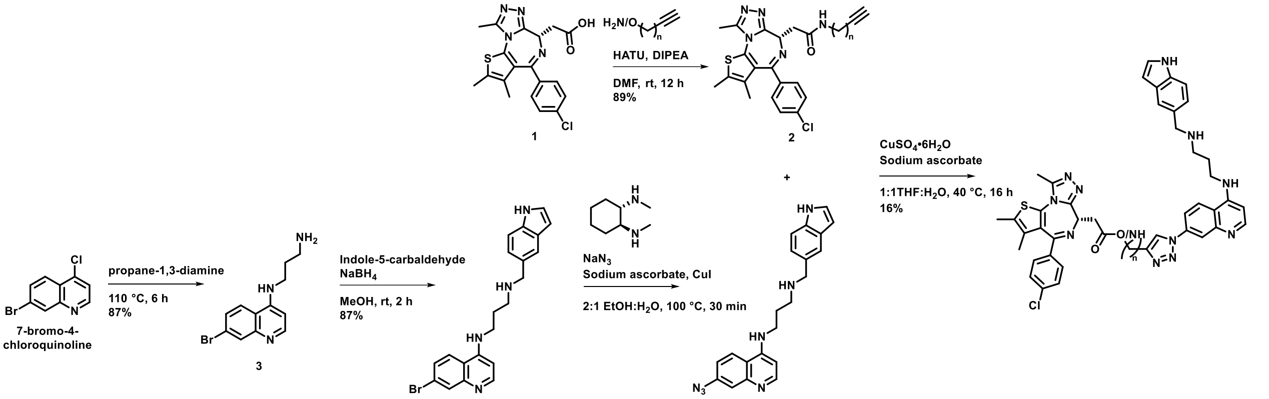

Supplementary Scheme 1: General Scheme for the Synthesis of PROTACs.

Intermediates used in PROTAC synthesis and an example linker for 105B:

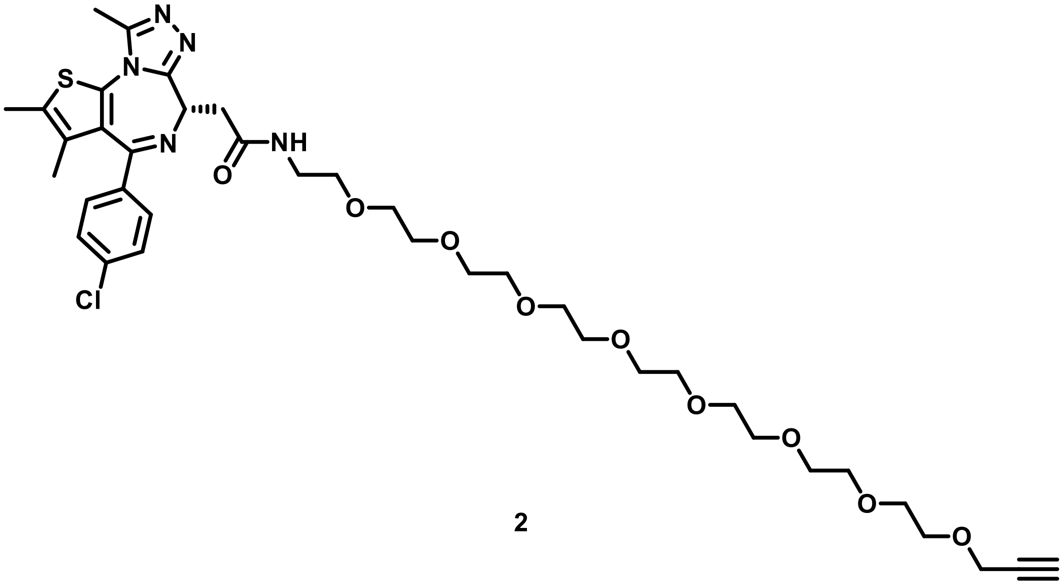

2

(*S*)-2-(4-(4-Chlorophenyl)-2,3,9-trimethyl-6*H*-thieno[3,2-f][1,2,4]triazolo[4,3-a][1,4]diazepin-6-yl)-*N*-(3,6,9,12,15,18,21,24-octaoxaheptacos-26-yn-1-yl)acetamide (2). Propargyl-PEG8-amine (125 mg, 1 eq), (+)-JQ1 carboxylic acid (123 mg, 1 eq), and HATU (128 mg, 1.1 eq) were dissolved in DMF (3.07 mL, 0.1 M). DIPEA (0.160 mL, 3 eq) was added, and the reaction was stirred at room temperature for 16 h. The crude reaction mixture was diluted in H_2O (5 mL). The product was extracted with DCM (3 x 10 mL), washed with brine (1 x 10 mL),

and dried over anhydrous $MgSO_4$. The mixture was concentrated *in vacuo* and purified via flash column chromatography (silica gel, 0-20% MeOH in DCM w/1% TEA) to afford **2** as a yellow oil (89% yield). ^{1}H NMR (400 MHz, C_2D_6SO w/0.1% TMS, standard residual internal C_2HD_5SO δ 2.50) δ 8.27 (br, 1H), 7.54 – 7.32 (m, 4H), 4.50 (t, *J* = 7.1 Hz, 1H), 4.13 (d, *J* = 2.5 Hz, 2H), 3.61 – 3.38 (m, 29H), 3.27-3.19 (m, 6H obscured by water), 2.66 (m, 1H), 2.59 (s, 3H), 2.41 (s, 3H), 2.32 (m, 1H), 1.62 (s, 3H). LCMS (ESI) calcd for $C_{38}H_{52}ClN_5O_9S$ 790.4, found 791.3 $[M+H]^+$.

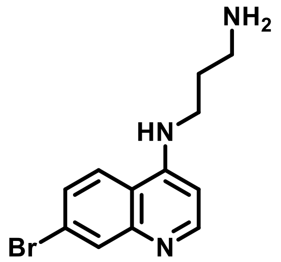

3

***N*¹-(7-Bromoquinolin-4-yl)propane-1,3-diamine (3).** 7-Bromo-4-chloroquinoline (1 g, 1 eq) was dissolved in 1,3-propanediamine (3 mL, 5.8 eq) and heated to 100 °C for 16 h. The reaction was cooled to room temperature, and 1 M NaOH (10 mL) and water (15 mL) were added, forming a white precipitate. The precipitate was filtered to afford **3** as an off-white powder. The product was used without further purification (Yield 87%). ^{1}H NMR (400 MHz, C_2D_6SO w/0.1% TMS, standard residual internal C_2D_5HSO δ 2.50) δ 8.37 (d, *J* = 5.4 Hz, 1H), 8.15 (d, *J* = 9.0 Hz, 1H), 7.93 (d, *J* = 2.1 Hz, 1H), 7.54 (d, *J* = 8.9, 2H), 7.54 (br, 1 H, overlapping with doublet), 6.48 (d, *J* = 5.5 Hz, 1H), 3.31 (t, *J* = 7.0 Hz, 2H, obscured by water), 2.68 (t, *J* = 6.5 Hz, 2H), 1.72 (p, *J* = 6.7 Hz, 2H). LCMS (ESI) calcd for $C_{12}H_{14}BrN_3$ 280.2 found, 281.0 $[M+H]^+$.

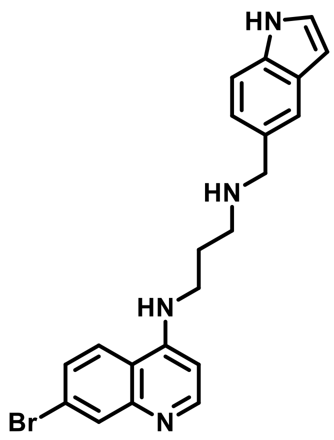

4

***N*¹-((1*H*-Indol-5-yl)methyl)-*N*3-(7-bromoquinolin-4-yl)propane-1,3-diamine (4).** Compound **3** (750 mg, 1 eq) and indole-5-carbaldehyde (389 mg, 1 eq) were dissolved in MeOH (27 mL, 0.1 M) and stirred for 1 h at rt. Sodium borohydride (50.6 mg, 0.5 eq) was added, and the reaction was stirred for an additional h. The reaction was quenched with H_2O, and the MeOH was removed *in vacuo*. The remaining aqueous layer was extracted with ethyl acetate (3 x 10mL), washed with brine (1x10 mL), and dried over anhydrous $NaSO_4$. The reaction mixture was concentrated *in vacuo* and purified via reverse phase flash column chromatography (C18, 5-100% ACN in H_2O) to afford **4** as an off-white powder (87% yield). ^{1}H NMR (400 MHz, C_2D_6SO w/0.1% TMS, standard residual internal C_2D_5HSO δ 2.50) δ 11.01 (s, 1H), 8.36 (d, *J* = 5.4 Hz, 1H), 8.01 (d, *J* = 9.0 Hz, 1H), 7.93 (d, *J* = 2.1 Hz, 1H), 7.67 (br, 1H), 7.48 (d, *J* = 1.5 Hz, 1H), 7.41 – 7.28 (m, 3H), 7.09 (dd, *J* = 8.3, 1.7 Hz, 1H), 6.45 (d, *J* = 5.5 Hz, 1H), 6.35 (t, *J* = 2.5 Hz, 1H), 3.77 (s, 2H), 3.32 (d, *J* = 13.5 Hz, 2H. Note: obscured by water), 2.69 (t, *J* = 6.4 Hz, 2H), 1.87-1.80 (m, 2H). ^{13}C NMR (400 MHz, C_2D_6SO) δ 152.3, 150.6, 149.8, 135.5, 131.4, 131.2, 128.0, 126.9, 125.8, 125.8, 124.45, 122.4, 122.3, 119.8, 118.1, 111.5, 101.3, 99.0, 54.2, 47.4, 41.9, 40.6, 40.4, 40.2, 40.0, 39.8, 39.6, 39.4, 28.1. LCMS (ESI) calcd for $C_{21}H_{21}BrN_4$ 409.3, found 410.1 $[M+H]^+$

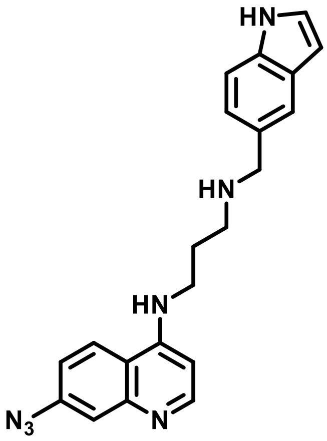

5

***N*[1]-((1*H*-Indol-5-yl)methyl)-*N*3-(7-azidoquinolin-4-yl)propane-1,3-diamine (5)**. To a flask under nitrogen was added **4** (50 mg, 1 eq), sodium azide (23.8 mg, 3 eq), sodium ascorbate (2.42 mg, 0.1 eq), copper iodide (2.33 mg, 0.1 eq), and (1*S*,2*S*)-N1,N2-dimethylcyclohexane-1,2-diamine (5.21 mg, 0.3 equiv.). The reaction mixture was dissolved in ethanol and water (2:1 ratio, 2 mL, 0.01 M) and heated in a microwave reactor (100 °C) for 30 minutes. The reaction was allowed to cool to room temperature and diluted with brine (5 mL). The product was extracted with THF (3 x 10 mL), washed with brine (10 mL), and dried with anhydrous $MgSO_4$. The product was concentrated under reduced pressure and purified via reverse phase flash column chromatography (C18, 5-100% ACN in H_2O) to afford **6** as a white powder**.** Identity confirmed via LCMS due to the inability to completely remove solvent *in vacuo*. LCMS (ESI) calcd for $C_{21}H_{21}N_7$ 371.5, found 372.2 $[M+H]^+$.

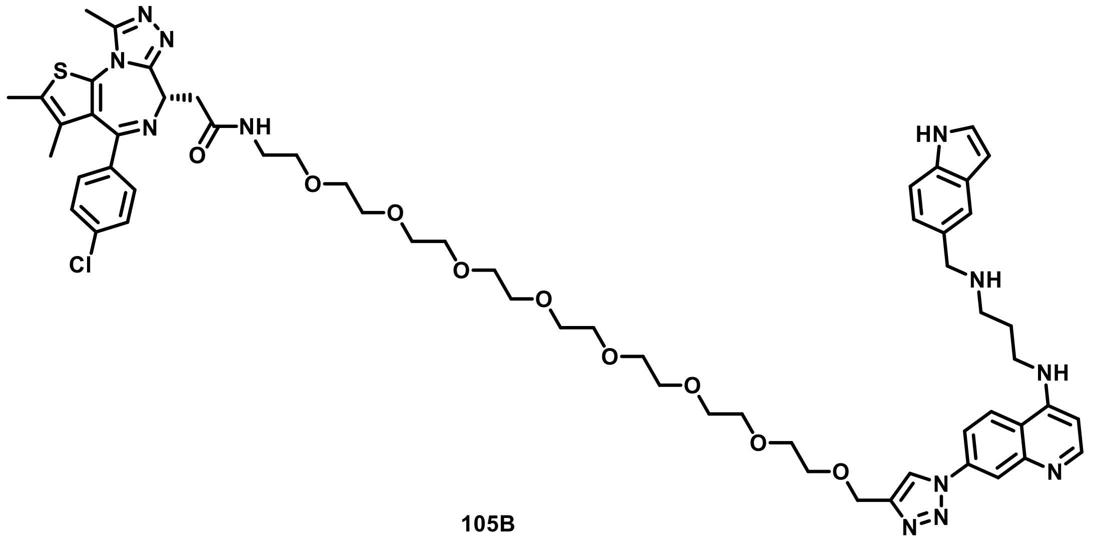

105B

(*S*)-*N*-(1-(1-(4-((3-(((1*H*-Indol-5-yl)methyl)amino)propyl)amino)quinolin-7-yl)-1*H*-1,2,3-triazol-4-yl)-2,5,8,11,14,17,20,23-octaoxapentacosan-25-yl)-2-(4-(4-Chlorophenyl)-2,3,9-trimethyl-6*H*-thieno[3,2-f][1,2,4]triazolo[4,3-a][1,4]diazepin-6-yl)acetamide (**105B**). Compound **5** (10 mg, 1.1 eq) and **2** (19.3 mg, 1 eq) were dissolved in H_2O/THF (1 mL total, 50:50 ratio, 0.18 M). $CuSO_4$•$6H_2O$ (0.6 mg, 0.1 eq) and sodium ascorbate (1 mg, 0.2 eq) were added, and the reaction was stirred at 45 °C for 16 h. The crude reaction mixture was concentrated in vacuo, diluted in MeOH (3 mL), and purified with ACCQPrep HP150 (Teledyne) equipped with a C18 column (Kinetex) (5-100% ACN in H_2O w/0.1% TFA) to afford the TFA salt of **105B** as an off-white powder (16% yield). ^{1}H NMR (700 MHz, , C_2D_6SO, standard residual internal C_2HD_5SO δ 2.50), δ 11.28 (s, 1H), 9.52 (t, *J* = 6.1 Hz, 1H), 9.08 (s, 2H), 9.04 (br, 1H), 8.72 (d, *J* = 9.3 Hz, 1H), 8.65 (d, *J* = 7.1 Hz, 1H), 8.53 (d, *J* = 2.2 Hz, 1H), 8.34 – 8.27 (m, 2H), 7.67 (d, *J* = 1.5 Hz, 1H), 7.51 – 7.35 (m, 6H), 7.19 (dd, *J* = 8.3, 1.6 Hz, 1H), 6.96 (d, *J* = 7.2 Hz, 1H), 6.46 – 6.40 (m, 1H), 4.68 (s, 2H), 4.52 (dd, *J* = 8.2, 5.9 Hz, 1H), 4.23 (t, *J* = 5.6 Hz, 2H), 3.66-3.65 (m, 4H), 3.61 – 3.56 (m, 2H), 3.55 – 3.39 (m, 28H), 3.33 – 3.18 (m, 4H), 3.10 (br, 2H), 2.59 (s, 3H), 2.39 (s, 3H), 1.61 (s, 3H). ^{13}C NMR (850 MHz, C_2D_6SO) δ 169.7, 163.1, 155.5, 145.9, 145.9, 143.5, 139.4, 138.6, 136.74, 136.67, 136.0, 135.4, 135.3, 132.2, 130.9, 130.2, 129.9, 129.7, 128.5, 127.7, 126.5,

126.0, 122.73, 122.68, 122.1, 121.9, 118.2, 116.1, 111.7, 109.7, 101.2, 98.8, 69.9, 69.82, 69.81, 69.77, 69.8, 69.7, 69.31, 69.25, 63.4, 53.8, 51.0, 43.8, 40.5, 38.7, 37.5, 24.4, 14.0, 12.7, 11.3. HRMS $[M+2H]^{2+}$ calcd for $C_{59}H_{73}ClN_{12}O_9S$ 581.2589. Found 581.2562. Purity: 96%.

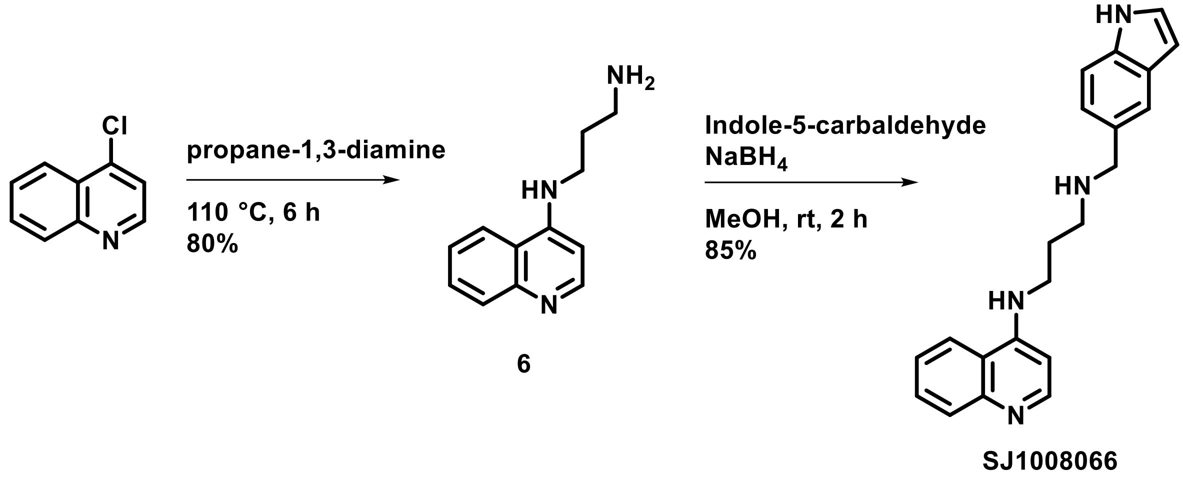

Supplementary Scheme 2: Synthetic route to SJ1008066.

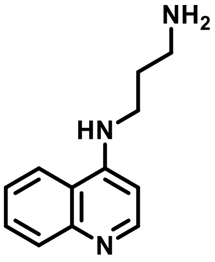

6

***N*[1]-(Quinolin-4-yl)propane-1,3-diamine (6).** 4-Chloroquinoline (1 g, 1 eq) was dissolved in 1,3-propanediamine (3mL, 5.8 eq) and heated to 100 °C for 16 h. The reaction was cooled to room temperature, and water (15 mL) was added, forming a white precipitate. The precipitate was filtered to afford **6** as an off-white powder. The product was used without further characterization, and its identity was confirmed via LCMS (80 % yield). LCMS(ESI): calcd for $C_{12}H_{15}N_3$ 201.3, found 202.1 $[M+H]^+$.

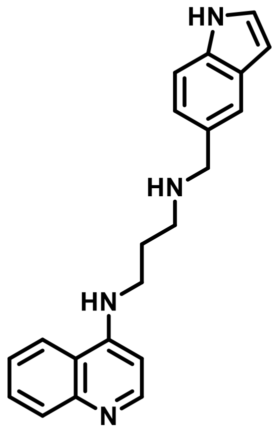

N[1]-((1*H*-Indol-5-yl)methyl)-*N*3-(quinolin-4-yl)propane-1,3-diamine (SJ1008066). Compound **6** (1 g, 1 eq) and indole-5-carbaldehyde (721 mg, 1 eq) were dissolved in MeOH (50 mL, 0.1 M) and stirred for 1 h at rt. Sodium borohydride (94 mg, 0.5 eq) was added, and the reaction was stirred for an additional h. The reaction was quenched with H_2O (10 mL), the MeOH was removed *in vacuo,* and the remaining aqueous layer was lyophilized overnight. The reaction mixture was purified via reverse phase flash column chromatography (C18, 5-100% ACN in water) to afford **5** as an off-white powder (85%). ^{1}H NMR (400 MHz, CD_3OD, standard residual internal CHD_2OD δ 3.31) δ 8.33 (d, *J* = 5.5 Hz, 1H), 7.98 (d, *J* = 8.6 Hz, 1H), 7.79 (d, *J* = 8.5 Hz, 1H), 7.62 (t, *J* = 6.9, 1H) 7.52 (s, 1H), 7.37-7.33 (m, 2H), 7.23 (d, *J* = 3.5 Hz, 1H), 7.10 (d, *J* = 8.2 Hz, 1H), 6.50 (d, J = 5.5 Hz, 1H), 6.39 (d, *J* = 3.8 Hz, 1H), 3.89 (s, 2H), 3.43 (t, *J* = 5.9 Hz, 2H), 2.85 (t, *J* = 6.7 Hz, 2H), 2.08 – 1.87 (m, 2H). ^{13}C NMR (600 MHz, CD_3OD/C_2D_6SO) δ 150.4, 149.2, 146.6, 34.8, 128.5, 128.2, 127.4, 126.7, 124.2, 123.6, 121.3, 120.4, 119.4, 118.11, 110.3, 110.3, 97.1, 52.8, 40.3, 26.4. Note: one ^{1}HNMR peak expected around 4.5 ppm was obscured by the MeOH peak. HRMS $[M+H]^+$ calcd for $C_{21}H_{22}N_4$: 331.1923, found 331.1901. Purity: 97%.

Characterization of PROTAC library

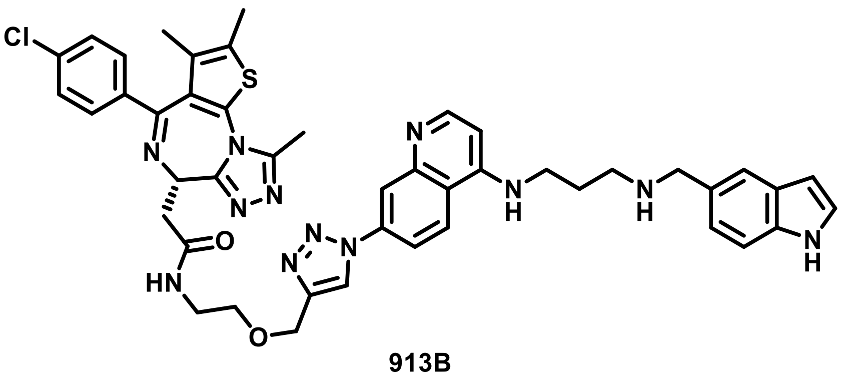

913B

(*S*)-*N*-(2-((1-(4-((3-(((1*H*-Indol-5-yl)methyl)amino)propyl)amino)quinolin-7-yl)-1*H*-1,2,3-triazol-4-yl)methoxy)ethyl)-2-(4-(4-chlorophenyl)-2,3,9-trimethyl-6*H*-thieno[3,2-f][1,2,4]triazolo[4,3-a][1,4]diazepin-6-yl)acetamide (913B). ^{1}H NMR (400 MHz, C_2D_6SO w/0.1% TMS, standard residual internal C_2HD_5SO δ 2.50) δ 11.24 (s, 1H), 9.07 (s, 1H), 8.50 - 8.48(m, 2H), 8.33 (m, 2H) 8.05 (dd, *J* = 9.1, 2.3 Hz, 1H), 7.69 (s, 1H), 7.49 – 7.34 (m, 6H), 7.23 (dd, *J* = 8.4, 1.7 Hz, 1H), 6.60 (d, *J* = 5.7 Hz, 1H), 6.44 (d, *J* = 2.7 Hz, 1H), 4.70 (s, 2H), 4.51 (dd, *J* = 8.1, 6.0 Hz, 1H), 4.20 (s, 2H), 3.59 (t, *J* = 5.8 Hz, 2H), 3.44 (s, 2H, obscured by water), 3.25-3.16 (m, 2H, obscured by water), 3.05 (t, *J* = 7.4 Hz, 3H), 2.58 (s, 3H), 2.38 (s, 3H), 2.00-2.01 (m, 2H), 1.59 (s, 3H), 1.24 (s, 2H). LCMS calcd for $C_{45}H_{45}ClN_{12}O_2S$: 853.4, found: 853.3 $[M]^{+}$. Purity: 98%.

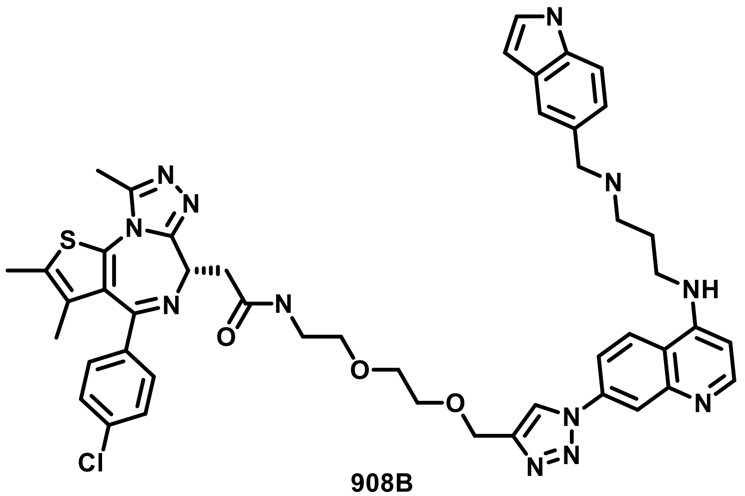

908B

(*S*)-*N*-(2-(2-((1-(4-((3-(((1*H*-Indol-5-yl)methyl)amino)propyl)amino)quinolin-7-yl)-1*H*-1,2,3-triazol-4-yl)methoxy)ethoxy)ethyl)-2-(4-(4-chlorophenyl)-2,3,9-trimethyl-6*H*-thieno[3,2-f][1,2,4]triazolo[4,3-a][1,4]diazepin-6-yl)acetamide (908B). ^{1}H NMR (400 MHz, C_2D_6SO w/0.1% TMS, standard residual internal C_2HD_5SO δ 2.50) δ 11.25 (s, 1H), 9.11 (br, 1H), 9.02 (s, 1H), 8.57 (d, *J* = 9.2 Hz, 1H), 8.50 (d, *J* = 5.8 Hz, 1H), 8.33-8.30 (m, 2H), 8.08 (dd, *J* = 9.2, 2.4 Hz, 2H), 7.71 (s, 1H), 7.52 – 7.31 (m, 6H), 7.30 – 7.18 (m, 1H), 6.65 (d, *J* = 5.9 Hz, 1H), 6.44 (t, *J* = 2.6 Hz, 1H), 4.68 (s, 2H), 4.52 (dd, *J* = 8.0, 6.1 Hz, 1H), 4.20 (s, 2H), 3.74 – 3.55 (m, 4H), 3.51-3.46 (m, 5H), 3.27 – 3.14 (m, 4H, note: obscured by water), 3.04 (t, *J* = 7.3 Hz, 2H), 2.57 (s, 3H), 2.35 (s, 3H), 2.09 (q, *J* = 7.3 Hz, 2H), 1.56 (s, 3H). Note: 4 expected hydrogens obscured by water. LCMS calcd for $C_{47}H_{49}ClN_{12}O_3S$ 897.5, found 897.4 $[M]^+$. Purity: 98%.

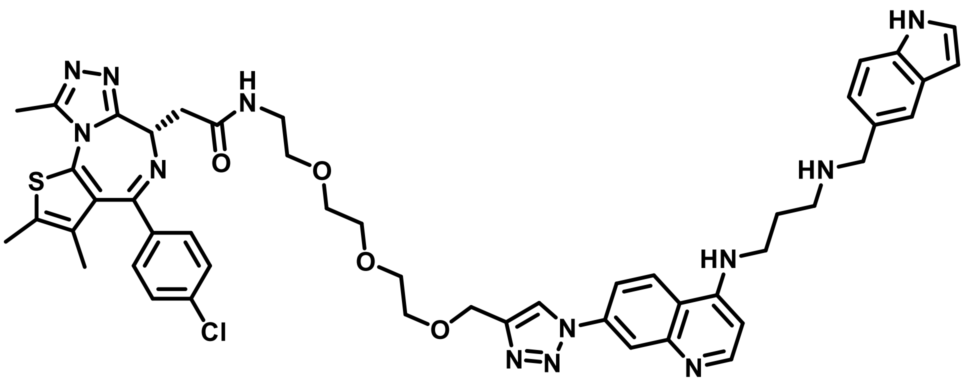

913C

(*S*)-*N*-(2-(2-(2-((1-(4-((3-(((1*H*-Indol-5-yl)methyl)amino)propyl)amino)quinolin-7-yl)-1*H*-1,2,3-triazol-4-yl)methoxy)ethoxy)ethoxy)ethyl)-2-(4-(4-chlorophenyl)-2,3,9-trimethyl-6*H*-thieno[3,2-f][1,2,4]triazolo[4,3-a][1,4]diazepin-6-yl)acetamide (913C). ^{1}H NMR (400 MHz, C_2D_6SO w/0.1% TMS, standard residual internal C_2HD_5SO δ 2.50) δ 11.25 (s, 1H), 9.72 (s, 1H),

9.28 (s, 2H), 9.07 (s, 1H), 8.91 (d, J = 9.2 Hz, 1H), 8.61 (d, J = 7.0 Hz, 1H), 8.53 (d, J = 2.2 Hz, 1H), 8.36 – 8.20 (m, 2H), 7.71 (s, 1H), 7.55 – 7.34 (m, 6H), 7.27 (dd, J = 8.4, 1.7 Hz, 1H), 6.93 (d, J = 7.1 Hz, 1H), 6.52 (s, 1H), 6.43 (s, 1H), 4.68 (s, 2H), 4.49 (dd, J = 8.0, 5.9 Hz, 1H), 4.19 (s, 2H), 3.72 – 3.63 (m, 4H), 3.59 (dd, J = 5.9, 3.3 Hz, 2H), 3.55 (s, 4H), 3.46 (t, J = 5.9 Hz, 3H), 3.33 (s, 16H), 3.29-3.17 (m, 4H, obscured by water), 3.05 (s, 2H), 2.57 (s, 3H), 2.38 (s, 3H), 2.12 (d, J = 7.1 Hz, 2H), 1.59 (s, 3H), 1.23 (s, 1H). Calcd for $C_{49}H_{53}ClN_{12}O_4S$ 941.6, found 941.3 $[M]^+$. Purity: 98%.

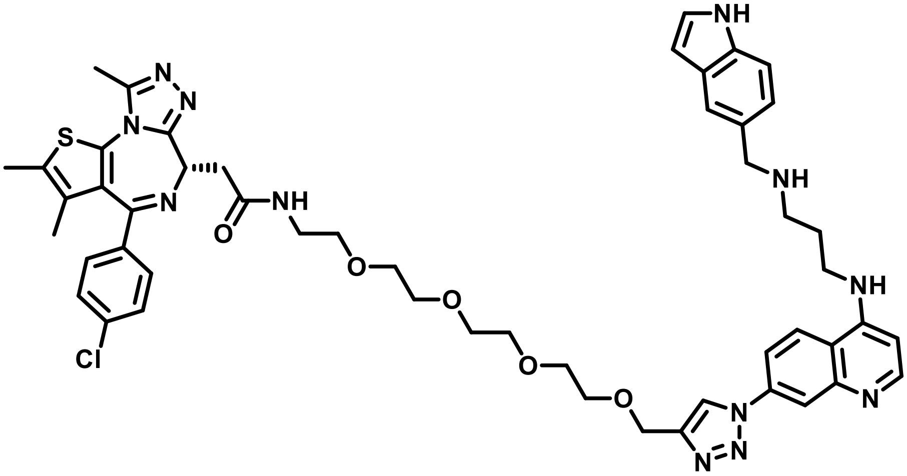

1024F

(*S*)-*N*-(1-(1-(4-((3-(((1*H*-Indol-5-yl)methyl)amino)propyl)amino)quinolin-7-yl)-1*H*-1,2,3-triazol-4-yl)-2,5,8,11-tetraoxatridecan-13-yl)-2-(4-(4-Chlorophenyl)-2,3,9-trimethyl-6*H*-thieno[3,2-f][1,2,4]triazolo[4,3-a][1,4]diazepin-6-yl)acetamide (1024F). ^{1}H NMR (400 MHz, C_2D_6SO w/0.1% TMS, standard residual internal C_2HD_5SO δ 2.50) δ 11.24 (s, 1H), 9.62 (br, 1H), 9.22 (br, 2H), 9.07 (s, 1H), 8.88 (d, J = 9.2 Hz, 1H), 8.61 (d, J = 6.9 Hz, 1H), 8.52 (d, J = 2.3 Hz, 1H), 8.31 – 8.20 (m, 2H), 7.71 (s, 1H), 7.50 – 7.37 (m, 8H), 7.26 (d, J = 8.5 Hz, 1H), 6.92 (d, J = 7.0 Hz, 1H), 6.52 (s, 1H), 6.43 (t, J = 2.5 Hz, 1H), 4.67 (s, 2H), 4.49 (dd, J = 8.1, 6.0 Hz, 1H), 4.20 (s, 2H), 3.69-3.63 (m, 5H, note: integral inflated by water), 3.59 – 3.56 (m, 3H), 3.52 (s, 10H),

3.45 (t, J = 5.9 Hz, 5H; integral inflated by water), 3.25 – 3.17 (m, 3H), 3.05 (s, 3H), 2.58 (s, 3H), 2.39 (s, 3H), 2.11-2.09 (m, 2H), 1.60 (s, 3H). LCMS calcd for $C_{51}H_{57}ClN_{12}O_5S$ 985.6, found 986.4 $[M+H]^+$. Purity: 89%.

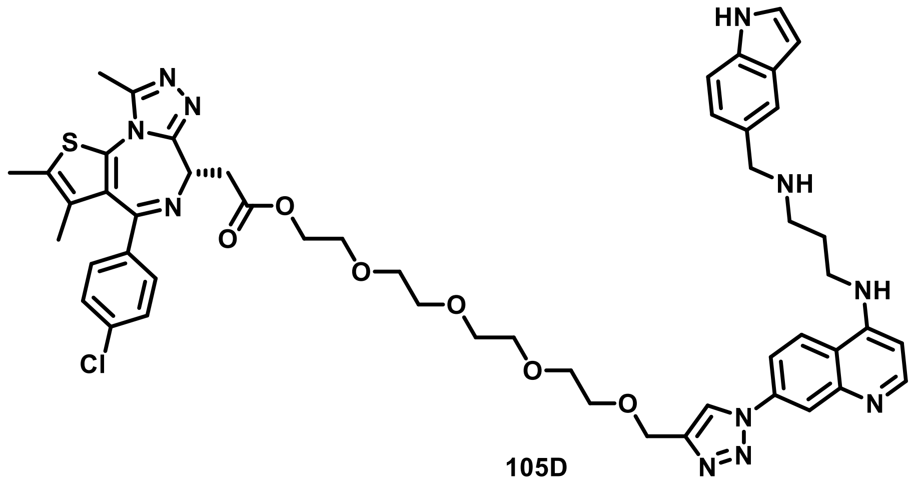

105D

1-(1-(4-((3-(((1*H*-Indol-5-yl)methyl)amino)propyl)amino)quinolin-7-yl)-1*H*-1,2,3-triazol-4-yl)-2,5,8,11-tetraoxatridecan-13-yl (*S*)-2-(4-(4-Chlorophenyl)-2,3,9-trimethyl-6*H*-thieno[3,2-f][1,2,4]triazolo[4,3-a][1,4]diazepin-6-yl)acetate (105D). ^{1}H NMR (400 MHz, C_2D_6SO w/0.1% TMS, standard residual internal C_2HD_5SO δ 2.50) δ 11.24 (s, 1H), 9.79 (br, 1H), 9.26 (br, 1H), 9.07 (s, 1H), 8.91 (d, J = 9.2 Hz, 1H), 8.63 (d, J = 6.9 Hz, 1H), 8.54 (s, 1H), 8.27-8.25 (m, 1H), 7.71 (s, 1H), 7.56 – 7.33 (m, 6H), 7.27 (d, J = 8.4 Hz, 1H), 6.95 (d, J = 7.2 Hz, 1H), 6.52-6.48 (m, 2H), 4.67 (s, 2H), 4.48 (t, J = 7.2 Hz, 1H), 4.23 – 4.16 (m, 3H), 3.66 – 3.45 (m, 18H; integral obscured by water), 3.07-3.03 (m, 2H), 2.59 (s, 3H), 2.41 (s, 3H), 2.13-2.10 (m, 2H), 1.62 (s, 3H), 1.23 (s, 1H). LCMS calcd for $C_{51}H_{56}ClN_{11}O_6S$ 986.6, found 1008.3 $[M + Na]^+$. Purity: 97%.

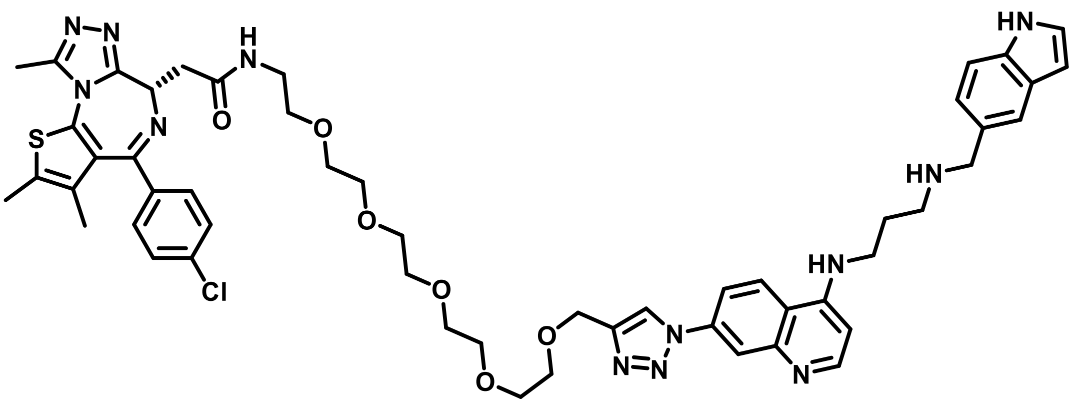

913D

(*S*)-*N*-(1-(1-(4-((3-(((1*H*-Indol-5-yl)methyl)amino)propyl)amino)quinolin-7-yl)-1*H*-1,2,3-triazol-4-yl)-2,5,8,11,14-pentaoxahexadecan-16-yl)-2-(4-(4-Chlorophenyl)-2,3,9-trimethyl-6*H*-thieno[3,2-f][1,2,4]triazolo[4,3-a][1,4]diazepin-6-yl)acetamide (913D). ^{1}H NMR (400 MHz, C_2D_6SO w/0.1% TMS, standard residual internal C_2HD_5SO δ 2.50) δ 11.25 (s, 1H), 9.17 (br, 1H), 9.06 (s, 1H), 8.77 (d, *J* = 9.1 Hz, 1H), 8.57 (d, *J* = 6.5 Hz, 1H), 8.45 (d, *J* = 2.2 Hz, 1H), 8.28 (t, *J* = 5.6 Hz, 1H), 8.19 (dd, *J* = 9.1, 2.3 Hz, 1H), 7.71 (s, 1H), 7.51 – 7.34 (m, 6H), 7.26 (dd, *J* = 8.4, 1.7 Hz, 1H), 6.82 (d, *J* = 6.5 Hz, 1H), 6.43 (t, *J* = 2.5 Hz, 1H), 4.67 (s, 2H), 4.50 (dd, *J* = 8.1, 6.0 Hz, 1H), 4.20 (s, 2H), 3.65 (dd, *J* = 5.8, 3.4 Hz, 2H), 3.63 – 3.55 (m, 4H), 3.55 – 3.48 (m, 12H), 3.44 (t, *J* = 5.9 Hz, 2H; obscured by water), 3.26 – 3.20 (m, 4H: obscured by water), 3.06-3.03 (m, 2H), 2.58 (s, 3H), 2.39 (s, 3H), 2.10 (m, 2H), 1.60 (s, 3H). LCMS calcd for $C_{53}H_{61}ClN_{12}O_6S$ 1029.6, found 1029.4 $[M]^+$. Purity: 97%.

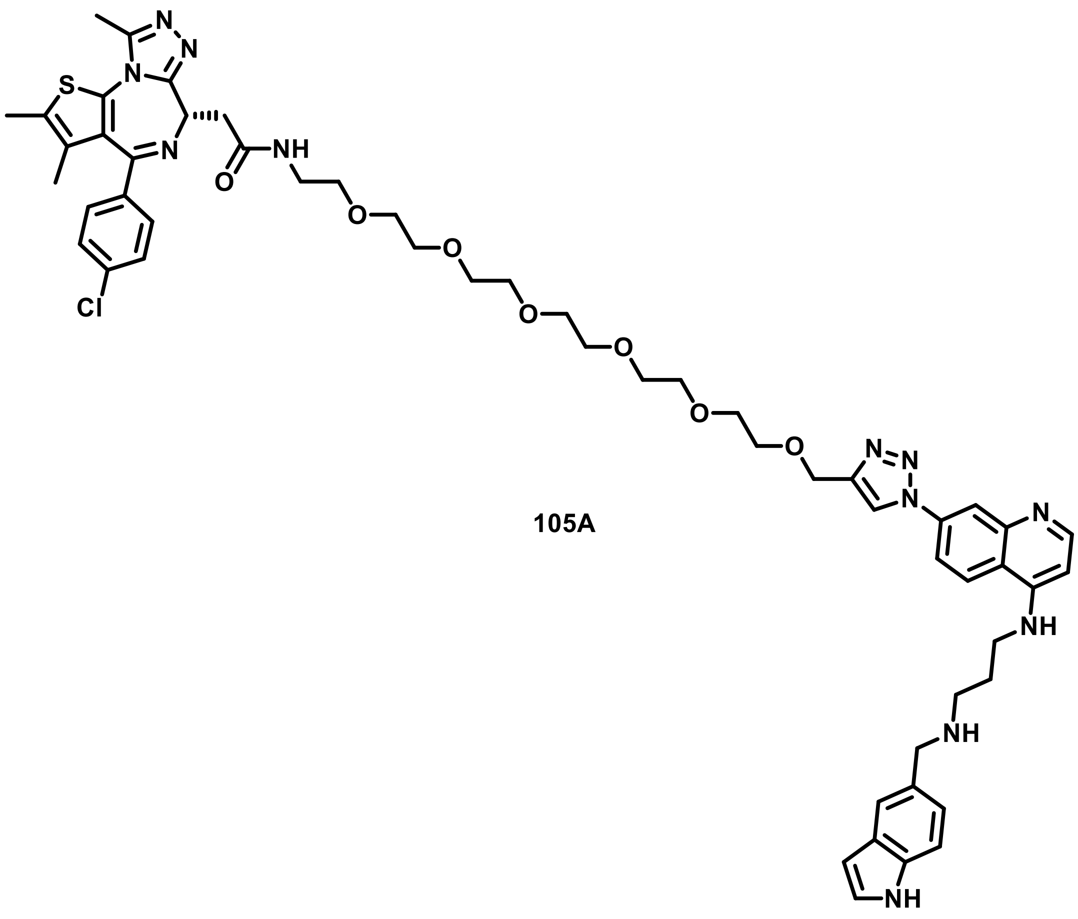

105A

(*S*)-*N*-(1-(1-(4-((3-(((1*H*-Indol-5-yl)methyl)amino)propyl)amino)quinolin-7-yl)-1*H*-1,2,3-triazol-4-yl)-2,5,8,11,14,17-hexaoxanonadecan-19-yl)-2-(4-(4-Chlorophenyl)-2,3,9-trimethyl-6*H*-thieno[3,2-f][1,2,4]triazolo[4,3-a][1,4]diazepin-6-yl)acetamide (105A). ^{1}H NMR (400 MHz, C_2D_6SO w/0.1% TMS, standard residual internal C_2HD_5SO δ 2.50) δ 11.25 (s, 1H), 9.82 (br, 1H), 9.27 (br, 1H), 9.08 (s, 1H), 8.93 (d, *J* = 9.2 Hz, 1H), 8.69 – 8.41 (m, 2H), 8.30-8.26 (m, 2H), 7.57 – 7.35 (m, 6H), 7.28 (d, *J* = 8.5 Hz, 1H), 6.95 (d, *J* = 7.1 Hz, 1H), 6.48 (m, 1H), 4.68 (s, 2H), 4.50 (t, *J* = 7.2 Hz, 1H), 4.20 (s, 2H), 3.71 – 3.62 (m, 4H), 3.60 – 3.55 (m, 3H), 3.54 – 3.45 (m, 16H), 3.44 (m, 5H, note: obscured by water), 3.06 (m, 2H), 2.59 (s, 3H), 2.40 (s, 3H),

2.12 (m, 2H), 1.61 (s, 3H), 1.24 (s, 2H). LCMS calcd for $C_{55}H_{65}ClN_{12}O_7S$ 1073.7, found 1095.3 $[M+Na]^+$. Purity: 96%.

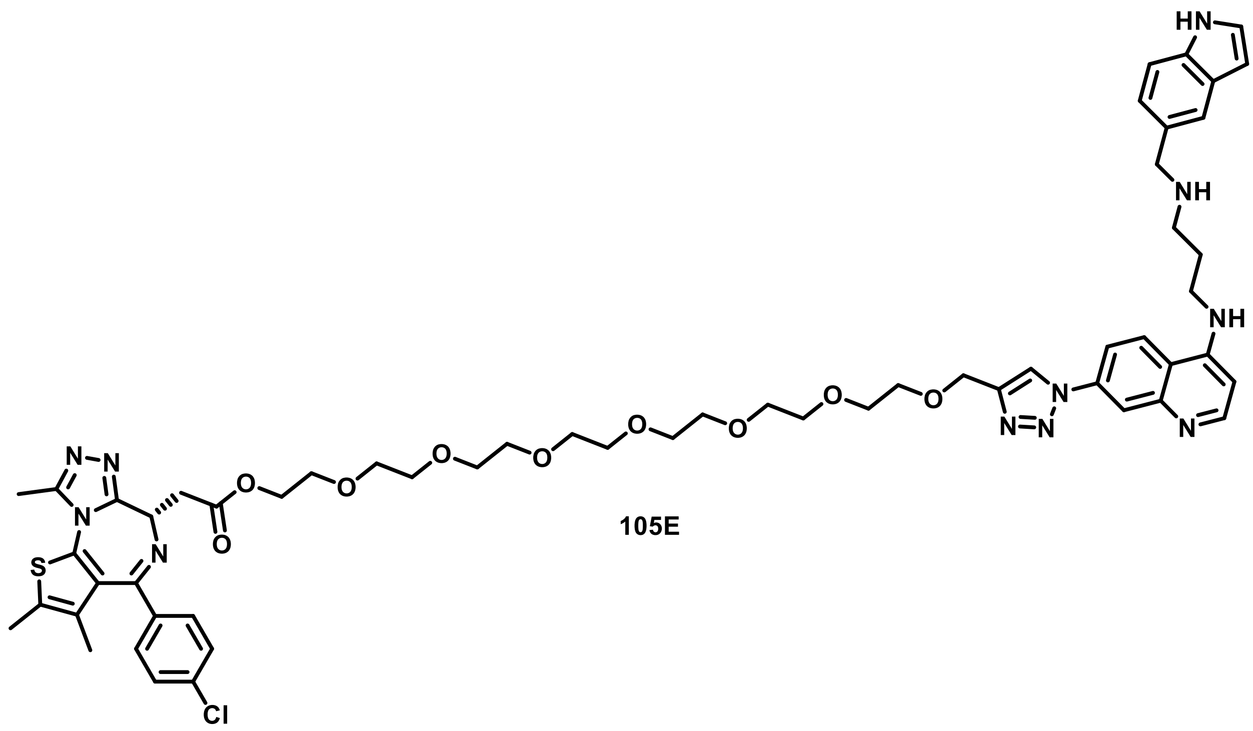

105E

1-(1-(4-((3-(((1*H*-Indol-5-yl)methyl)amino)propyl)amino)quinolin-7-yl)-1*H*-1,2,3-triazol-4-yl)-2,5,8,11,14,17,20-heptaoxadocosan-22-yl (*S*)-2-(4-(4-Chlorophenyl)-2,3,9-trimethyl-6*H*-thieno[3,2-f][1,2,4]triazolo[4,3-a][1,4]diazepin-6-yl)acetate (105E). ^{1}H NMR (400 MHz, C_2D_6SO w/0.1% TMS, standard residual internal C_2HD_5SO δ 2.50) δ 11.24 (s, 1H), 9.66 (br 1H), 9.15 (s, 1H), 9.07 (s, 1H), 8.87 (d, *J* = 9.1 Hz, 1H), 8.63 (d, *J* = 6.8 Hz, 1H), 8.51 (d, *J* = 2.1 Hz, 1H), 8.27 (d, *J* = 8.8 Hz, 1H), 7.71 (s, 1H), 7.56 – 7.33 (m, 6H), 7.32 – 7.20 (m, 1H), 6.94 (d, *J* = 7.0 Hz, 1H), 6.52 (s, 1H), 4.68 (s, 2H), 4.48 (t, *J* = 7.1 Hz, 1H), 4.26 – 4.13 (m, 4H), 3.69 – 3.54 (m, 10H), 3.50-3.47 (m, 21H, note: integral inflated by water), 3.06 (br, 2H), 2.59 (s, 3H), 2.41 (ts, 3H), 2.10 (br, 2H), 1.62 (s, 3H), 1.23 (s, 2H). LCMS calcd for $C_{57}H_{68}ClN_{11}O_9S$ 1118.75, found 1140.3 $[M + Na]^+$. Purity: 91%.

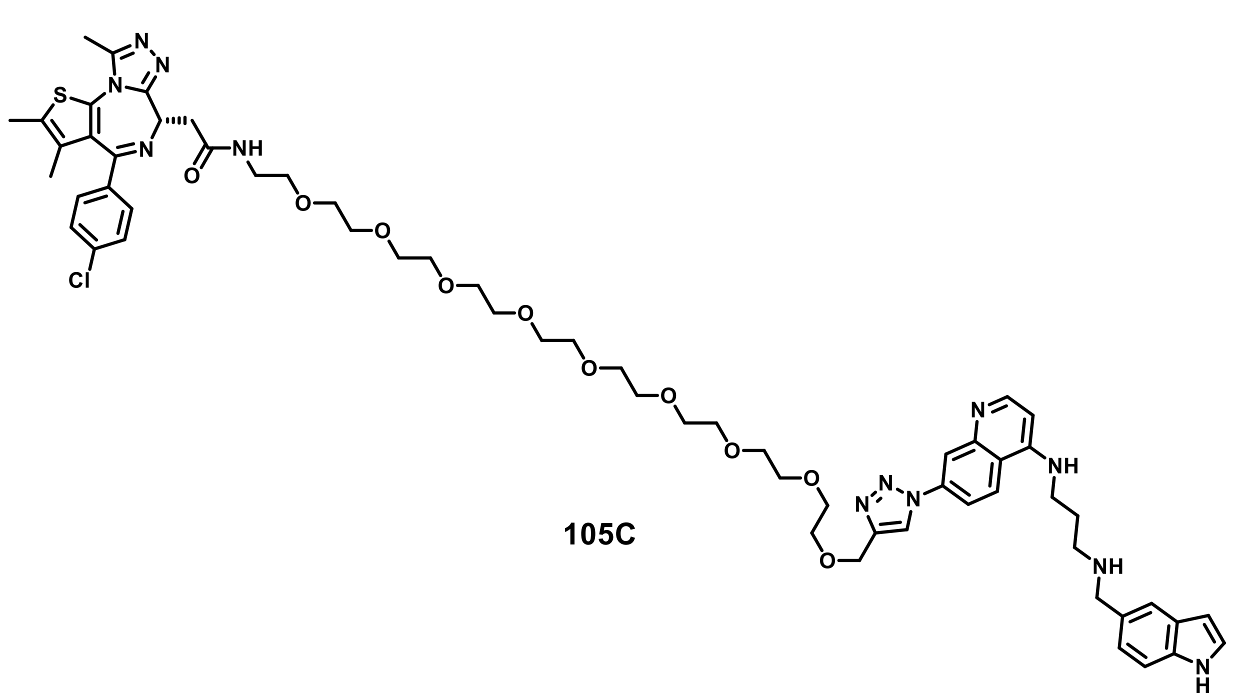

105C

(*S*)-*N*-(1-(1-(4-((3-(((1*H*-Indol-5-yl)methyl)amino)propyl)amino)quinolin-7-yl)-1*H*-1,2,3-triazol-4-yl)-2,5,8,11,14,17,20,23,26-nonaoxaoctacosan-28-yl)-2-(4-(4-Chlorophenyl)-2,3,9-trimethyl-6*H*-thieno[3,2-f][1,2,4]triazolo[4,3-a][1,4]diazepin-6-yl)acetamide (105C). ^{1}H NMR (400 MHz, C_2D_6SO w/0.1% TMS, standard residual internal C_2HD_5SO δ 2.50) δ 11.26 (s, 1H), 9.90 (br, 1H), 9.38 (br, 1H), 9.02 (m, 2H), 8.65 – 8.43 (m, 2H), 8.29 (t, *J* = 6.6 Hz, 1H), 8.15 (br, 1H), 7.56 – 7.27 (m, 7H), 6.95 (dd, *J* = 7.3, 3.2 Hz, 1H), 6.47 (d, *J* = 39.7 Hz, 1H), 6.92 (s, 1H), 6.42 (s, 1H), 4.67 (d, *J* = 7.3 Hz, 2H), 4.50 (t, *J* = 7.0 Hz, 1H), 4.20 (s, 2H), 3.68 – 3.63 (m, 3H), 3.55 – 3.41 (m, 32H), 3.05 (m, 3H), 2.58 (s, 3H), 2.40 (d, *J* = 5.2 Hz, 3H), 2.13 (s, 3H), 1.61 (d, *J* = 5.1 Hz, 3H), 1.30 – 1.16 (m, 1H). Note: 8 hydrogens obscured by water. LCMS calcd for $C_{61}H_{77}ClN_{12}O_{10}S$ 1205.9, found 1205.5 $[M]^+$. Purity: 96%.

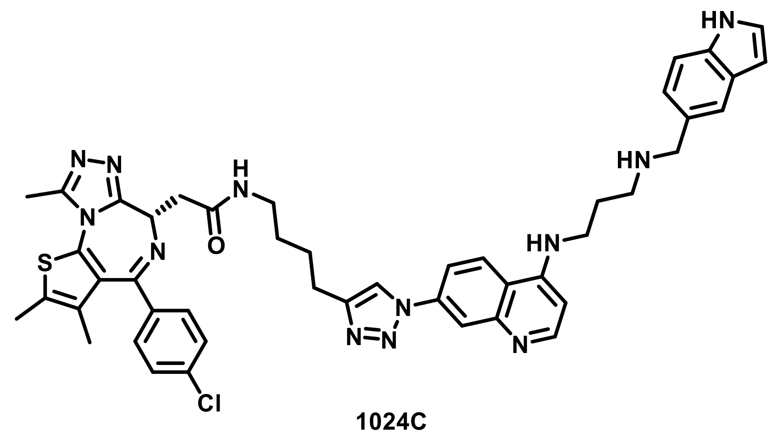

1024C

(*S*)-*N*-(4-(1-(4-((3-(((1*H*-Indol-5-yl)methyl)amino)propyl)amino)quinolin-7-yl)-1*H*-1,2,3-triazol-4-yl)butyl)-2-(4-(4-Chlorophenyl)-2,3,9-trimethyl-6*H*-thieno[3,2-f][1,2,4]triazolo[4,3-a][1,4]diazepin-6-yl)acetamide (1024C). ^{1}H NMR (400 MHz, C_2D_6SO w/0.1% TMS, standard residual internal C_2HD_5SO δ 2.50) δ 11.16 (1H, s), 8.74 (1H, s), 8.42-8.36 (2H, m) 8.19-8.14 (2H, m)f, 7.93- 7.91 (1H, dd), 7.61 (1H, s), 7.41-7.31 (5H, m) 7.17- 7.15 (1H, dd), 6.48-6.47 (1H, d) 6.36 (1H, s), 4.47-4.44 (1H, t), 4.09 (2H, s), 3.21 (appx. 5H, obscured by water), 2.92 (2H, t), 2.70 (2H, t), 2.53 (3H, s) 2.30 (3H, s) 2.01- 1.94 (2H, m), 1.84- 1.65 (2H, m), 1.54-1.47 (1H, m), 1.50 (3H, s). ^{13}C NMR (600 MHz, C_2D_6SO w/0.1% TMS, standard residual internal C_2HD_5SO) δ 169.9, 163.5, 155.6, 152.5, 150.4, 150.3, 148.7, 137.3, 137.2, 136.3, 135.7, 132.7, 131.1, 130.6, 130.3, 130.0, 128.9, 128.0, 126.8, 124.7, 123.2, 122.3, 120.8, 118.6, 116.2, 112.0, 101.6, 54.4, 51.8, 40.6, 40.4, 40.2, 40.0, 39.8, 39.6, 39.4, 38.7, 38.2, 29.2, 26.5, 25.2, 14.5, 13.1, 11.8. LCMS calcd for $C_{46}H_{47}ClN_{12}OS$ 851.5, found 851.3 $[M]^{+}$. Purity: 97%.

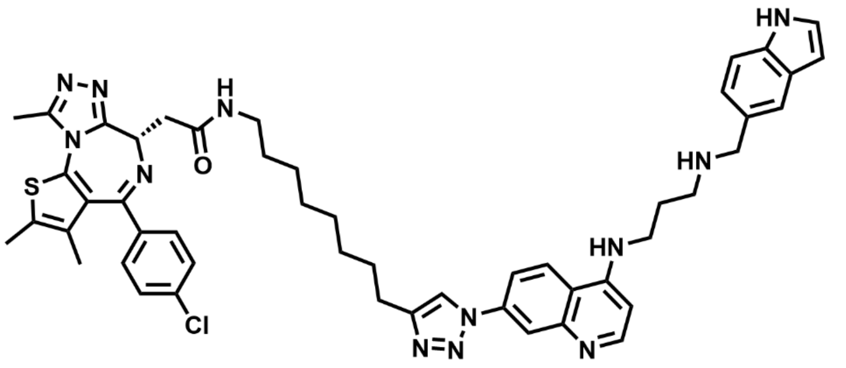

1024B

(*S*)-*N*-(8-(1-(4-((3-(((1*H*-Indol-5-yl)methyl)amino)propyl)amino)quinolin-7-yl)-1*H*-1,2,3-triazol-4-yl)octyl)-2-(4-(4-Chlorophenyl)-2,3,9-trimethyl-6*H*-thieno[3,2-f][1,2,4]triazolo[4,3-a][1,4]diazepin-6-yl)acetamide (1024B). ^{1}H NMR (400 MHz, C_2D_6SO w/0.1% TMS, standard residual internal C_2HD_5SO δ 2.50) δ 11.24 (s, 1H), 9.10 (br, 2H), 8.81 (s, 1H), 8.70 (d, *J* = 9.1 Hz, 1H), 8.56 (d, *J* = 6.3 Hz, 1H), 8.39 (d, *J* = 2.2 Hz, 1H), 8.16 (m, 2H), 7.70 (s, 1H), 7.56 – 7.34 (m, 6H), 7.25 (dd, *J* = 8.3, 1.7 Hz, 1H), 6.77 (d, *J* = 6.4 Hz, 1H), 6.44 (t, *J* = 2.5 Hz, 1H), 4.50 (dd, *J* = 8.1, 5.9 Hz, 1H), 4.20 (s, 2H), 3.56 (s, 2H), 3.24 – 3.01 (m, 6H obscured by water), 2.73 (t, *J* = 7.5 Hz, 2H), 2.58 (s, 3H), 2.39 (s, 3H), 2.09 (m, 2H), 1.68 (t, *J* = 7.4 Hz, 2H), 1.60 (s, 3H), 1.45 (m, 2H), 1.31 (m, 9H). LCMS calcd for $C_{50}H_{55}ClN_{12}OS$ 907.6, found 907.4 $[M]^{+}$. Purity: 98%

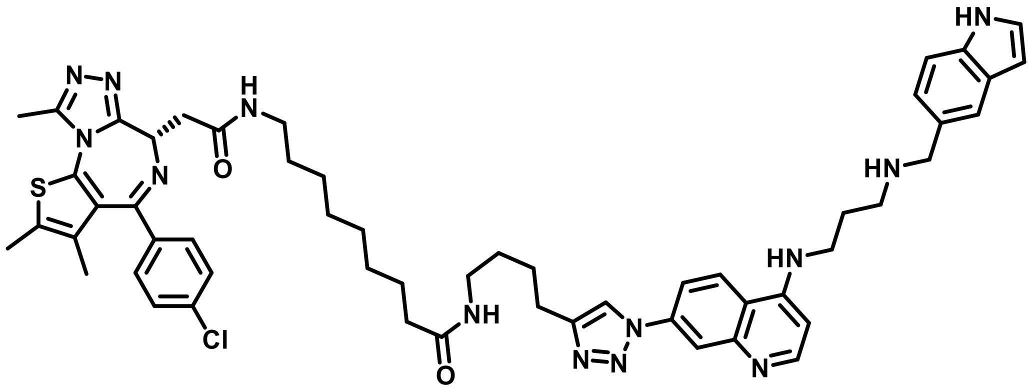

1024E

(*S*)-*N*-(4-(1-(4-((3-(((1*H*-Indol-5-yl)methyl)amino)propyl)amino)quinolin-7-yl)-1*H*-1,2,3-triazol-4-yl)butyl)-9-(2-(4-(4-Chlorophenyl)-2,3,9-trimethyl-6*H*-thieno[3,2-f][1,2,4]triazolo[4,3-a][1,4]diazepin-6-yl)acetamido)nonanamide (1024E). ^{1}H NMR (400 MHz, C_2D_6SO w/0.1% TMS, standard residual internal C_2HD_5SO δ 2.50) δ 11.24 (s, 1H), 9.17 (br, 2H), 8.82 (d, *J* = 11.0 Hz, 2H), 8.59 (d, *J* = 6.7 Hz, 1H), 8.46 (s, 1H), 8.19 (mi, 2H), 7.77 (d, *J* = 5.8 Hz, 1H), 7.71 (s, 1H), 7.49 – 7.37 (m, 6H), 7.26 (d, *J* = 8.4 Hz, 1H), 6.87 (d, *J* = 6.8 Hz, 1H), 6.43 (d, *J* = 3.0 Hz, 1H), 4.49 (dd, *J* = 8.1, 5.9 Hz, 1H), 4.2920(s, 2H), 3.63 (m, 2H), 3.18 – 2.97 (m, 7H), 2.75 (t, *J* = 7.4 Hz, 2H), 2.58 (s, 3H), 2.40 (s, 3H), 2.14 – 2.00 (m, 4H), 1.74 – 1.64 (m, 2H), 1.60 (s, 3H), 1.54 – 1.35 (m, 6H), 1.22 (d, *J* = 6.3 Hz, 8H). ^{13}C NMR (600 MHz, C_2D_6SO w/0.1% TMS) δ 172.4, 169.8, 163.4, 155.6, 150.3, 149.1, 139.3, 137.2, 136.4, 135.7, 132.7, 131.2, 130.6, 130.3, 130.0, 128.9, 128.0, 126.9, 126.4, 123.4, 122.7, 122.4, 121.0, 117.9, 116.9, 112.0, 101.6, 99.2, 54.4, 51.3, 44.2, 40.6, 40.4, 40.2, 40.0, 39.8, 39.6, 39.4, 38.9, 38.5, 38.1, 35.9, 35.9, 29.7, 29.1, 26.8, 26.6, 25.8, 25.1, 24.7, 14.5, 13.2, 11.8. LCMS calcd for $C_{55}H_{64}ClN_{13}O_2S$ 1006.7, found 1006.5 $[M]^+$. Purity: 97%.

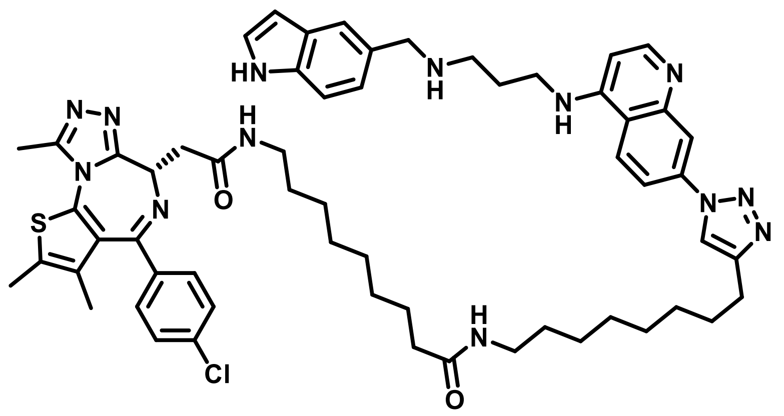

1024D

(*S*)-*N*-(8-(1-(4-((3-(((1*H*-Indol-5-yl)methyl)amino)propyl)amino)quinolin-7-yl)-1*H*-1,2,3-triazol-4-yl)octyl)-9-(2-(4-(4-Chlorophenyl)-2,3,9-trimethyl-6*H*-thieno[3,2-f][1,2,4]triazolo[4,3-a][1,4]diazepin-6-yl)acetamido)nonanamide (1024D). ^{1}H NMR (400 MHz, C_2D_6SO w/0.1% TMS, standard residual internal C_2HD_5SO δ 2.50) δ 11.26 (s, 1H), 9.18 (br, 2H), 8.81 (s, 1H), 8.69 (d, *J* = 9.2 Hz, 1H), 8.53 (d, *J* = 6.2 Hz, 1H), 8.37 (d, *J* = 2.3 Hz, 1H), 8.26 – 8.01 (m, 2H), 7.72 (m, 2H), 7.55 – 7.36 (m, 6H), 7.27 (dd, *J* = 8.4, 1.7 Hz, 1H), 6.73 (d, *J* = 6.3 Hz, 1H), 6.44 (t, *J* = 2.4 Hz, 1H), 4.50 (dd, *J* = 8.1, 5.9 Hz, 1H), 4.20 (s, 2H), 3.55 (d, *J* = 7.0 Hz, 2H), 3.03 (m, predicted 5H obscured by water), 2.72 (t, *J* = 7.5 Hz, 2H), 2.58 (s, 3H), 2.39 (s, 3H), 2.09 (t, *J* = 7.3 Hz, 2H), 2.01 (t, *J* = 7.3 Hz, 2H), 1.67 (q, *J* = 7.4 Hz, 2H), 1.60 (s, 3H), 1.50 – 1.12 (m, 24H). LCMS calcd for $C_{59}H_{72}ClN_{13}O_2S$ 1062.8, found 1062.6 $[M]^+$. Purity: 96%.

References

(1) Jiang, J.; Liang, T.; Solberg, J.; Chan, A.; Kalra, P.; Shi, R.; Pomerantz, W. C. K.; Hawkinson, J. E.; Schönbrunn, E.; Georg, G. I. Macrocyclic dihydropyridine analogs as pan-BET BD2-preferred inhibitors. *Eur. J. Med. Chem.* **2025**, *290*, 117504. DOI: 10.1016/j.ejmech.2025.117504.

NMR Spectra

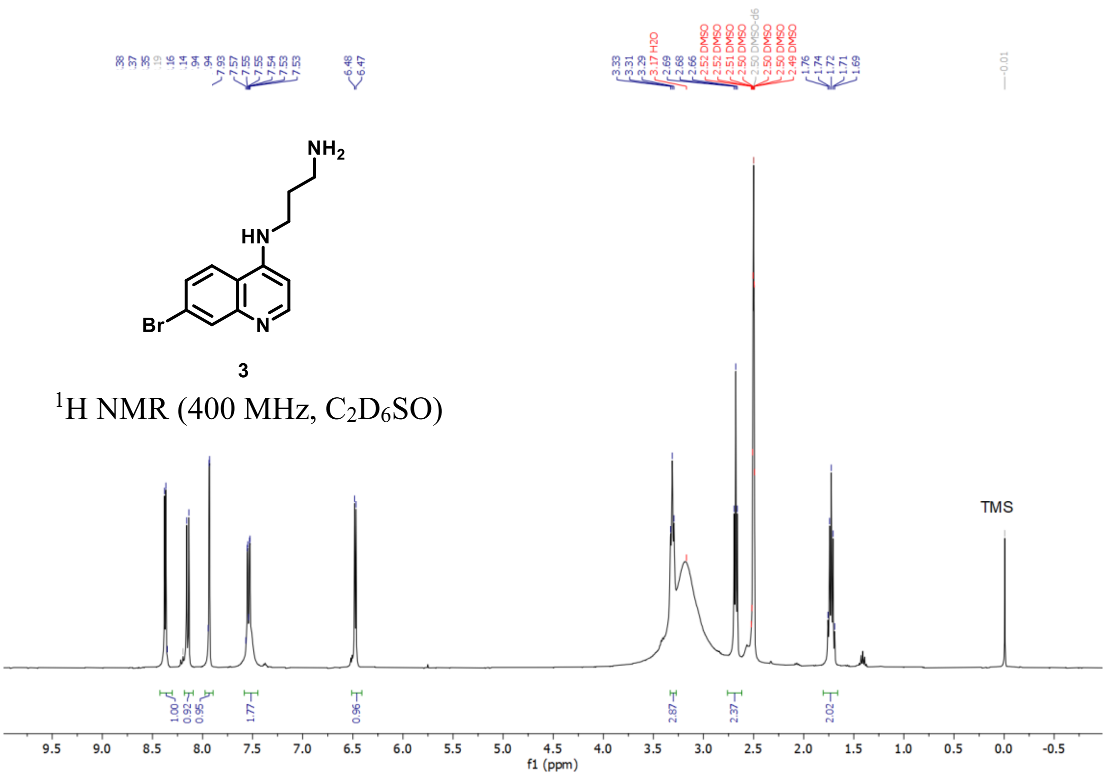

3

^{1}H NMR (400 MHz, C_2D_6SO)

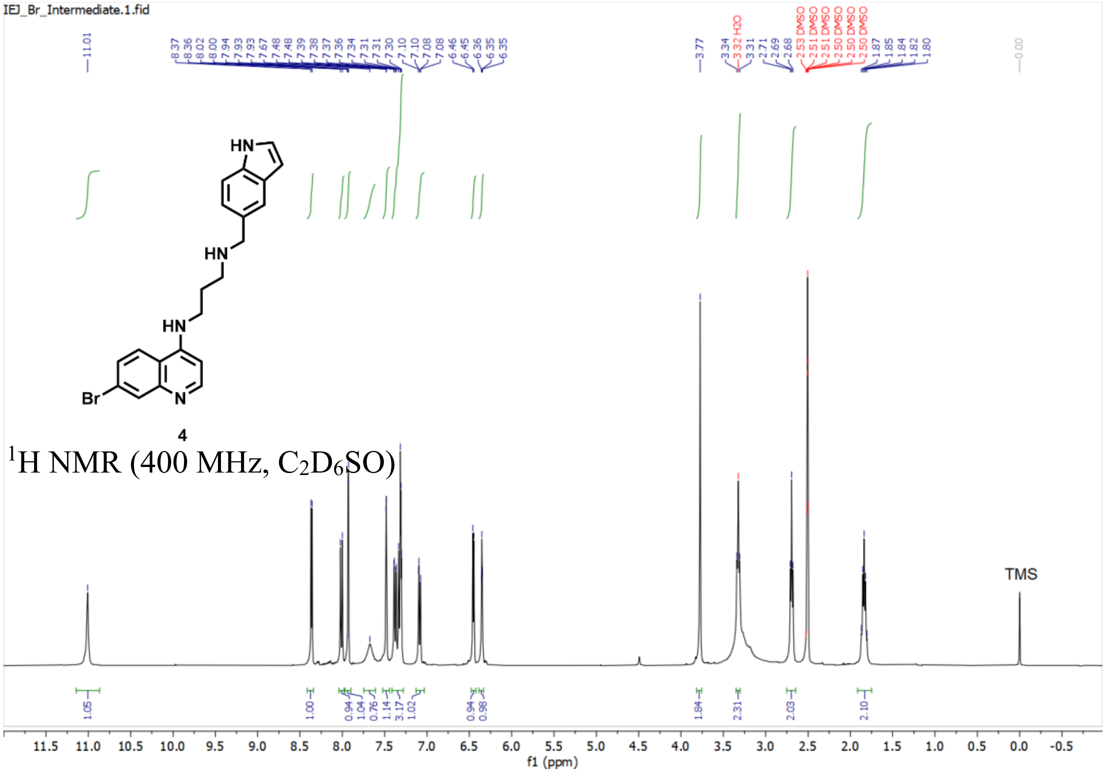

4

^{1}H NMR (400 MHz, C_2D_6SO)

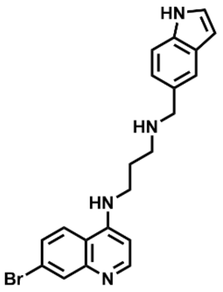

4

^{13}C NMR (400 MHz, C_2D_6SO)

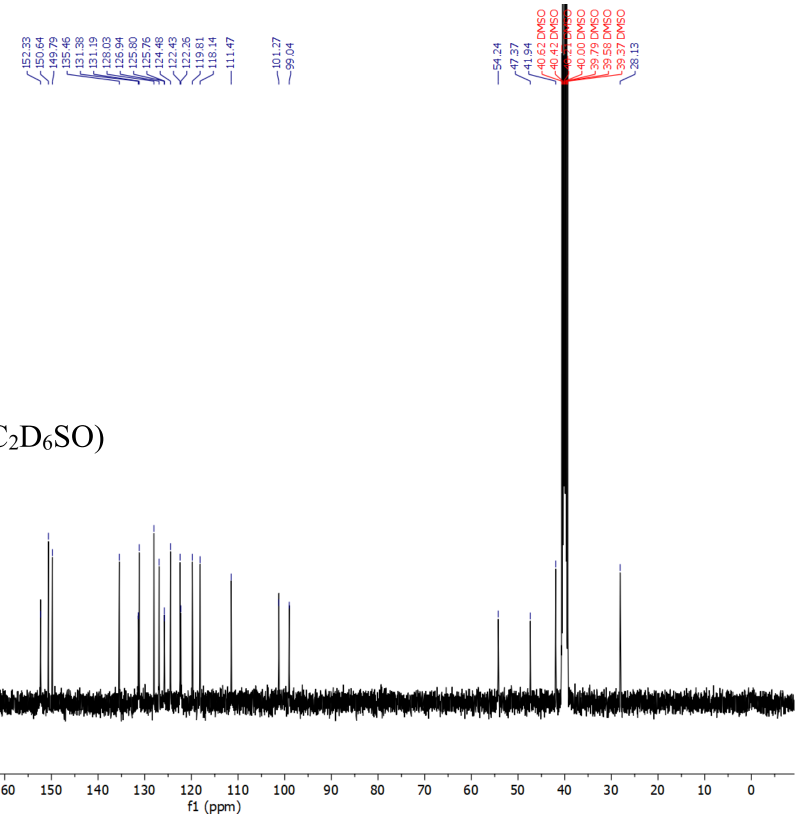

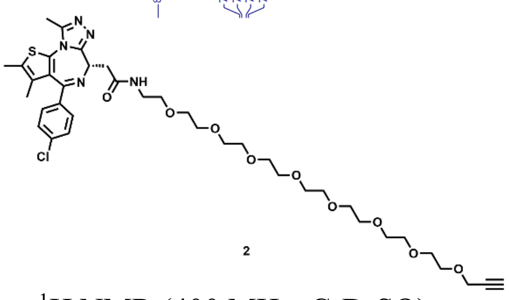

2

^{1}H NMR (400 MHz, C_2D_6SO)

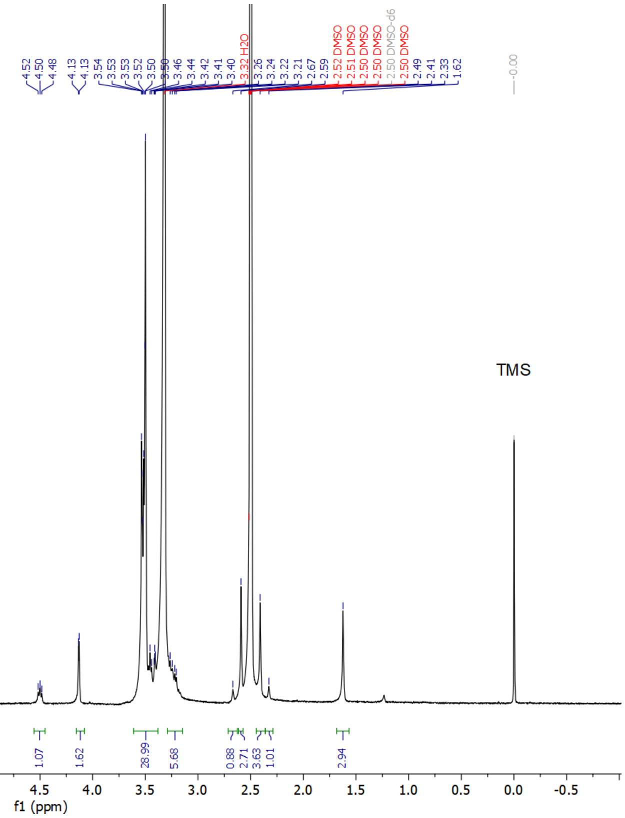

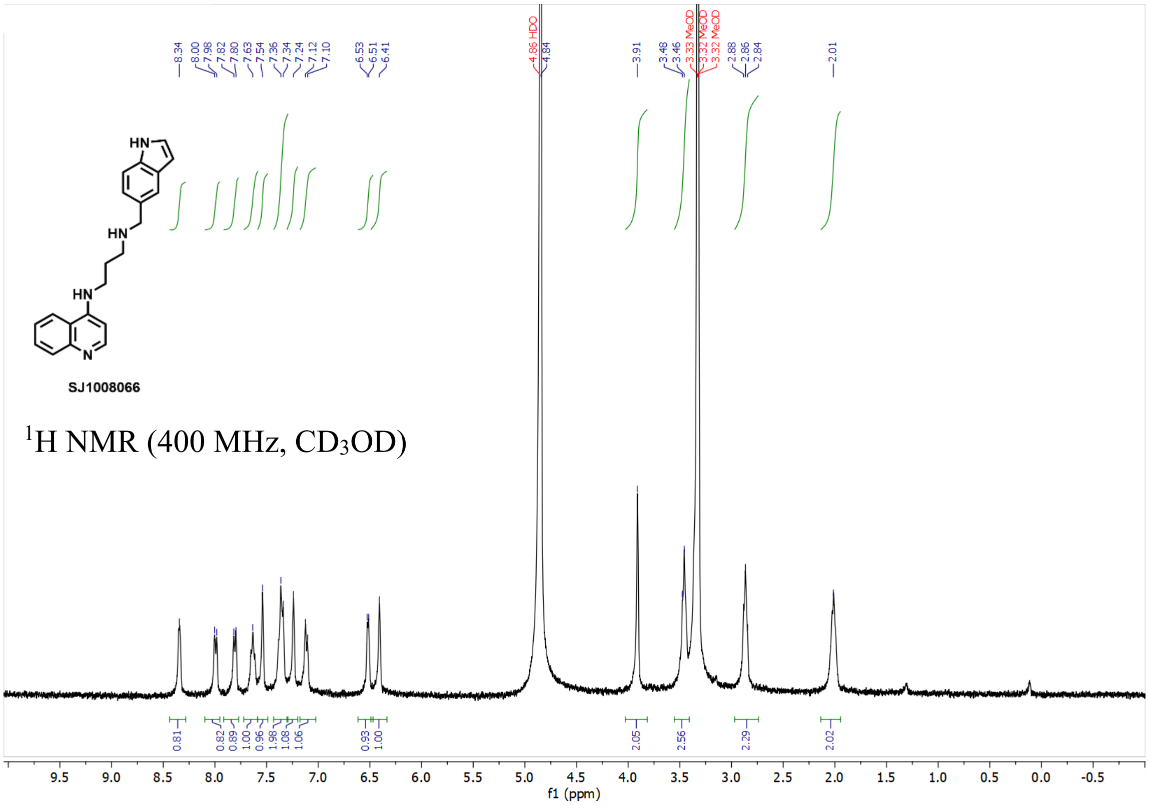
SJ1008066
^{1}H NMR (400 MHz, CD_3OD)
f1 (ppm)

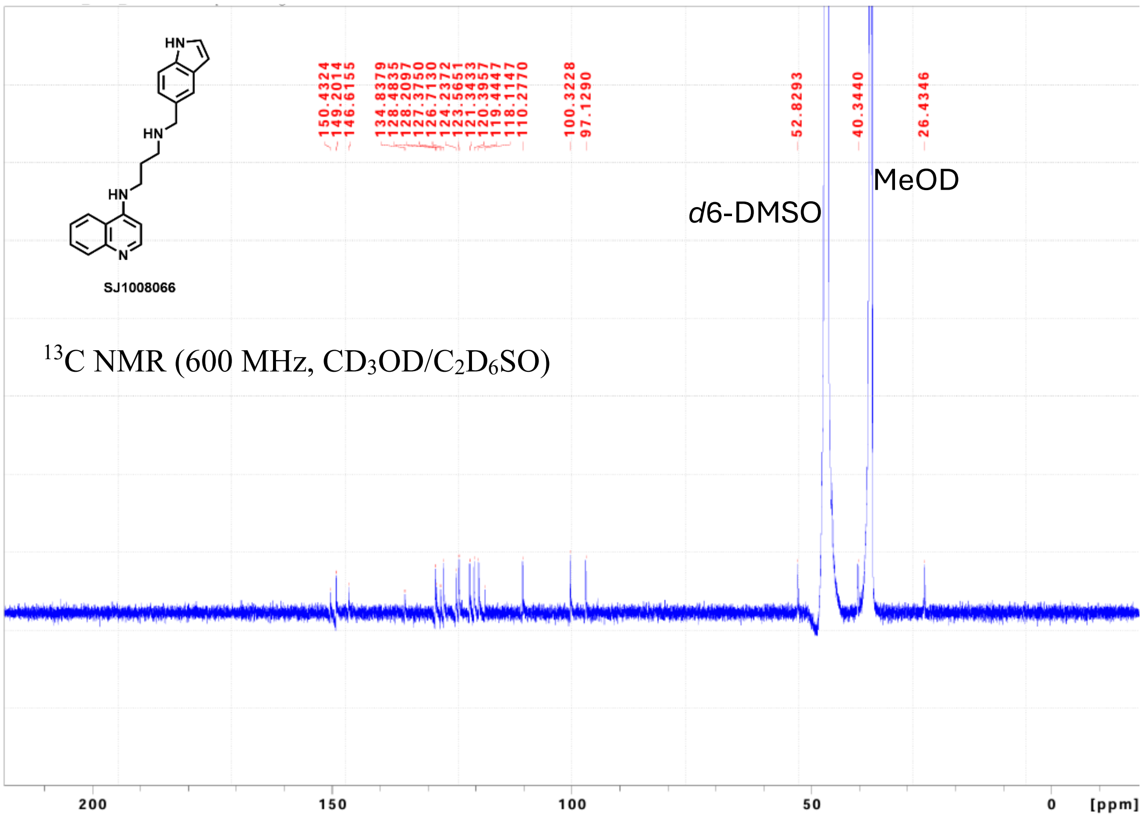
SJ1008066
^{13}C NMR (600 MHz, CD_3OD/C_2D_6SO)
d6-DMSO
MeOD
[ppm]

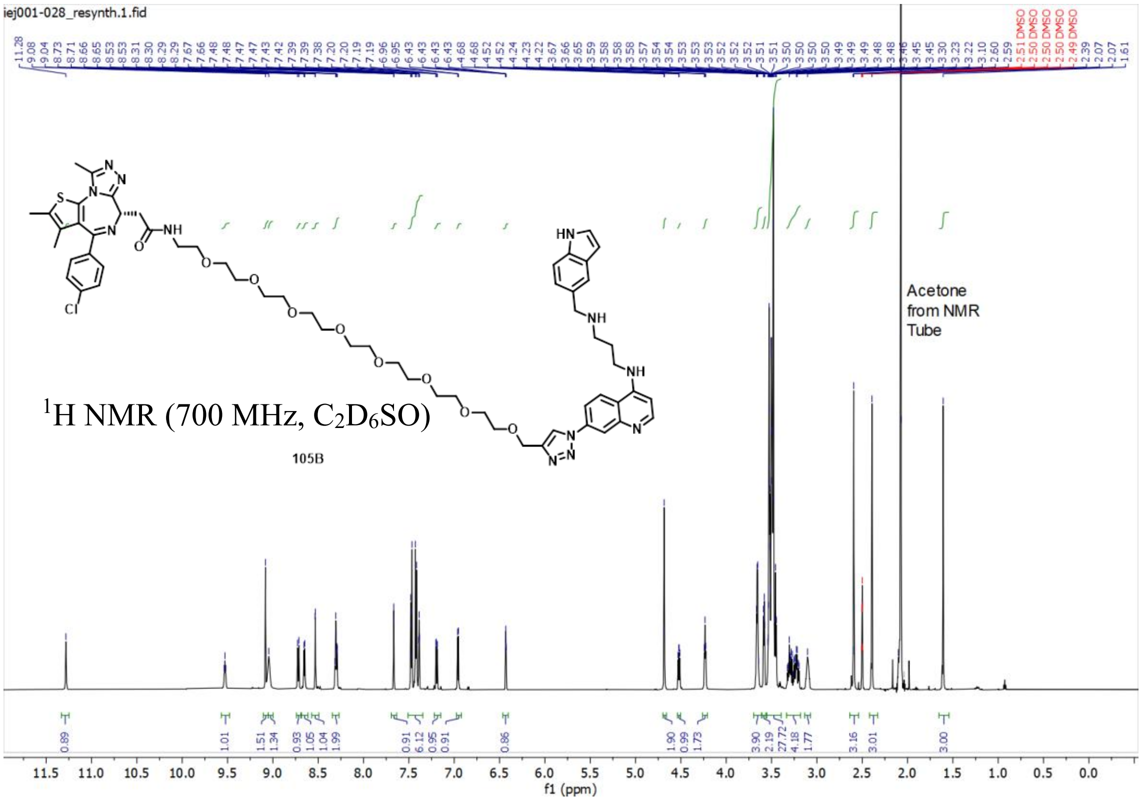
iej001-028_resynth.1.fid
Acetone from NMR Tube
^{1}H NMR (700 MHz, C_2D_6SO)
105B
f1 (ppm)

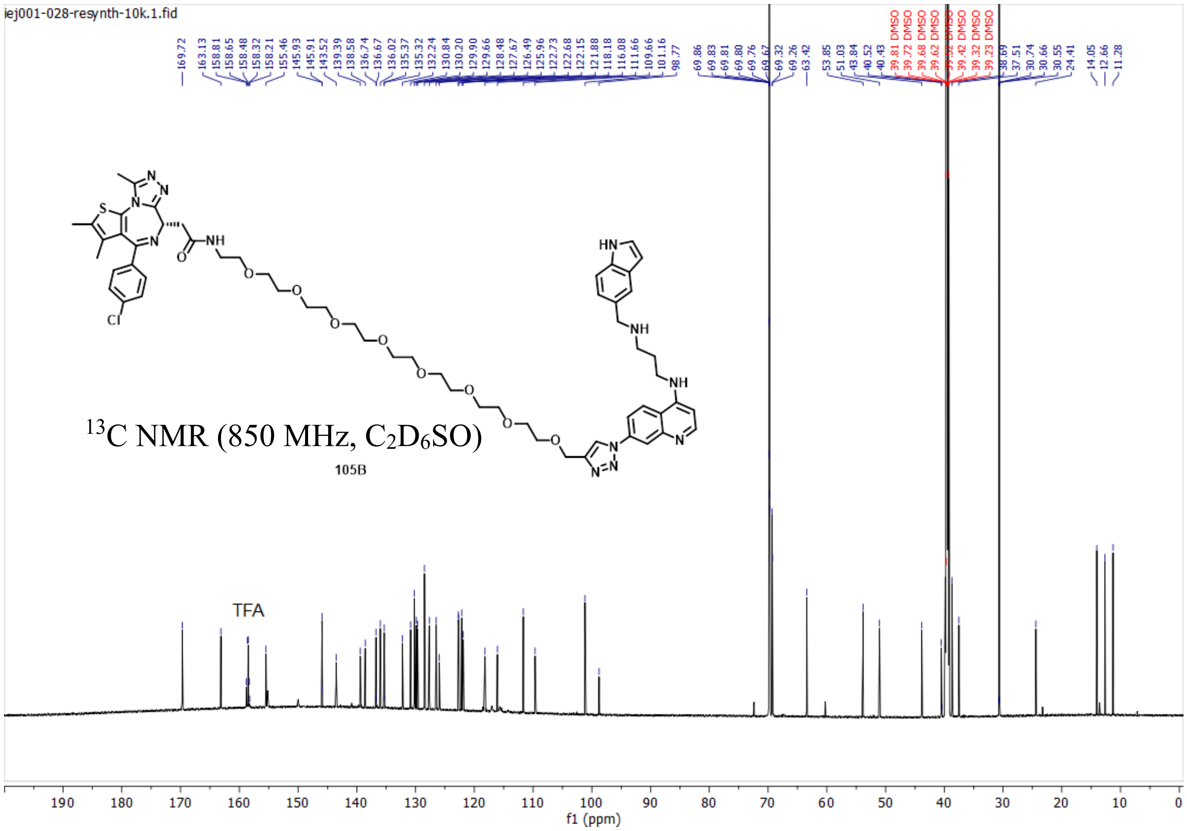
iej001-028-resynth-10k.1.fid
TFA
^{13}C NMR (850 MHz, C_2D_6SO)
105B
f1 (ppm)

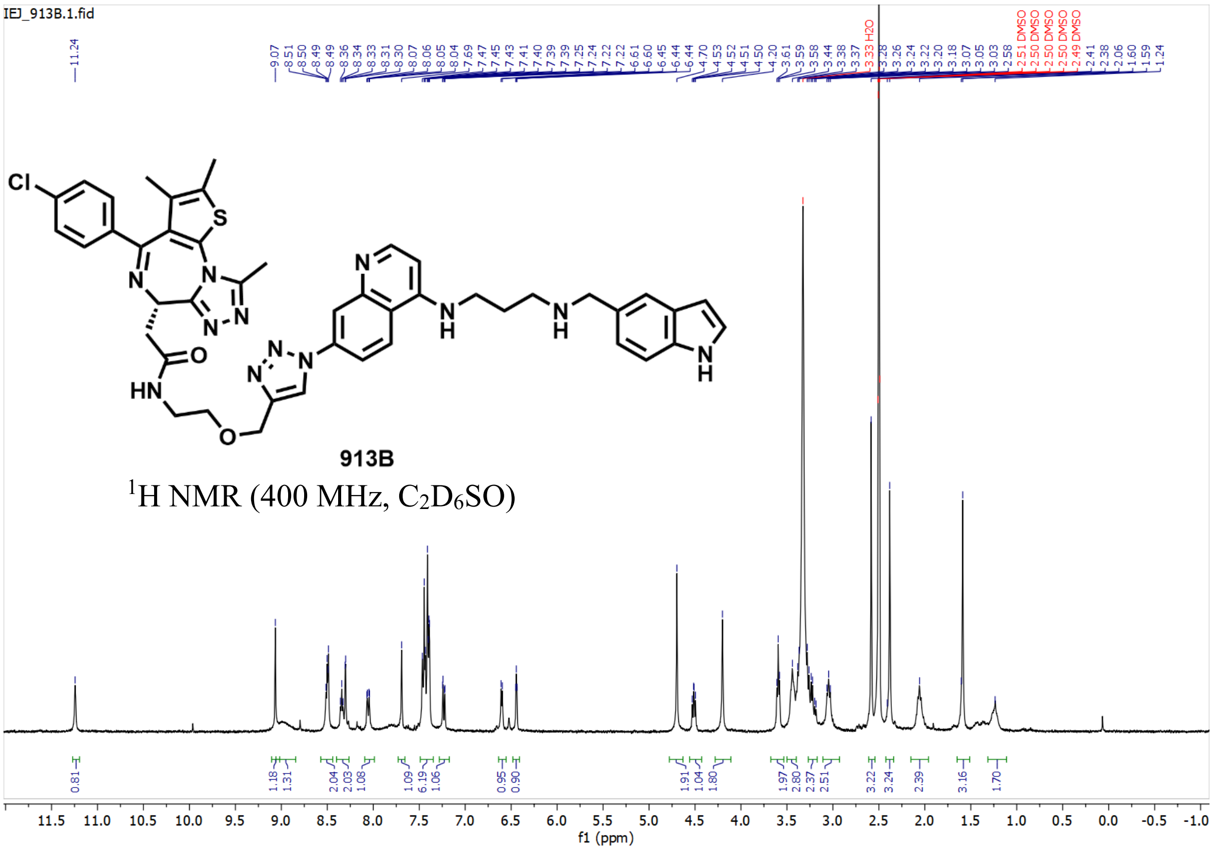
IEJ_913B.1.fid
913B
^{1}H NMR (400 MHz, C_2D_6SO)
f1 (ppm)

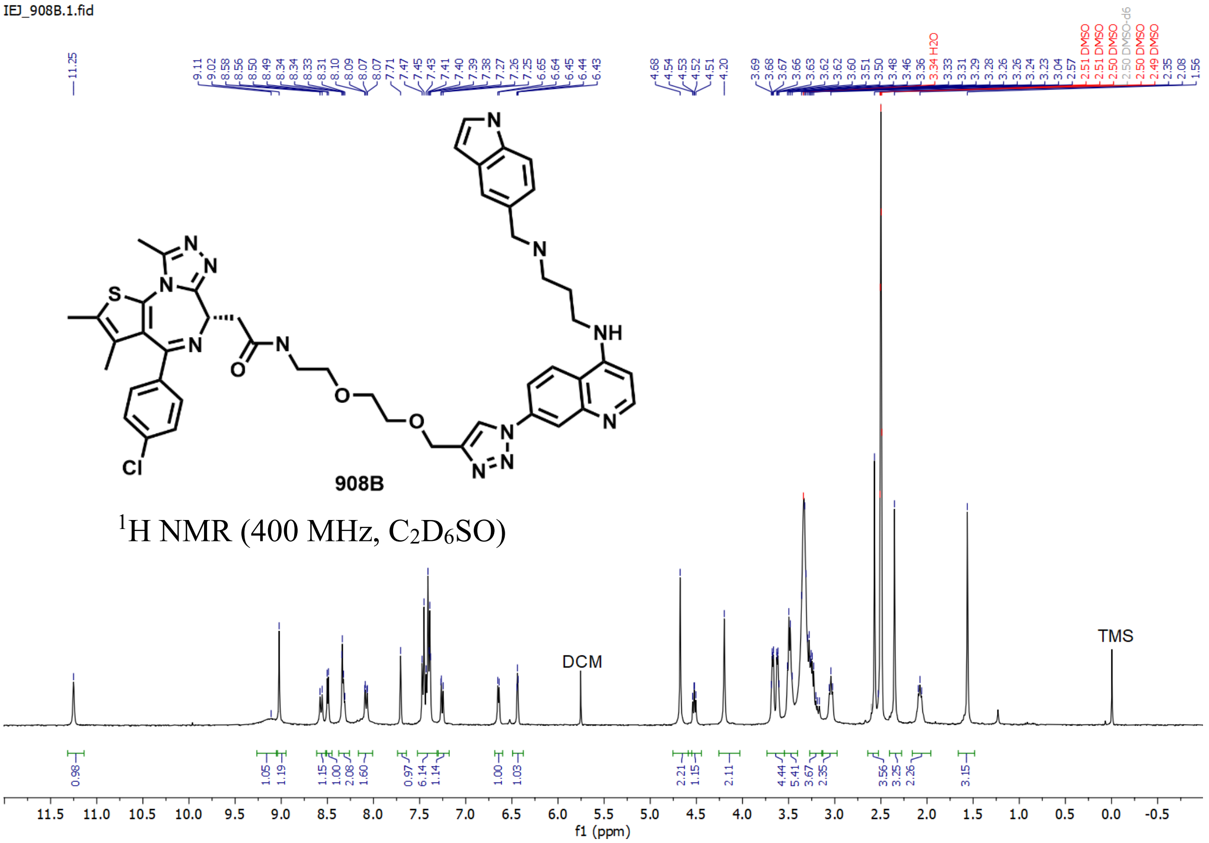
IEJ_908B.1.fid
908B
^{1}H NMR (400 MHz, C_2D_6SO)
DCM
TMS
f1 (ppm)

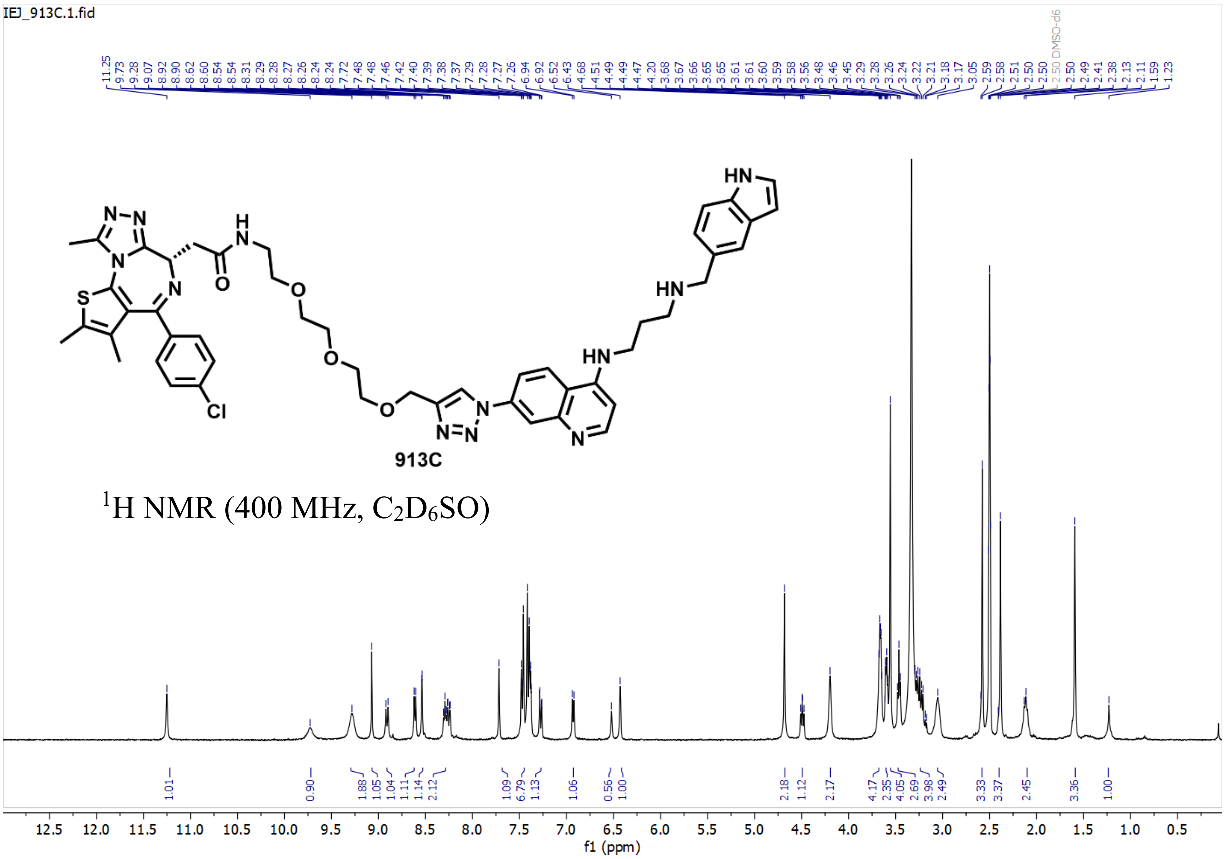
IEJ_913C.1.fid
913C
^{1}H NMR (400 MHz, C_2D_6SO)
f1 (ppm)

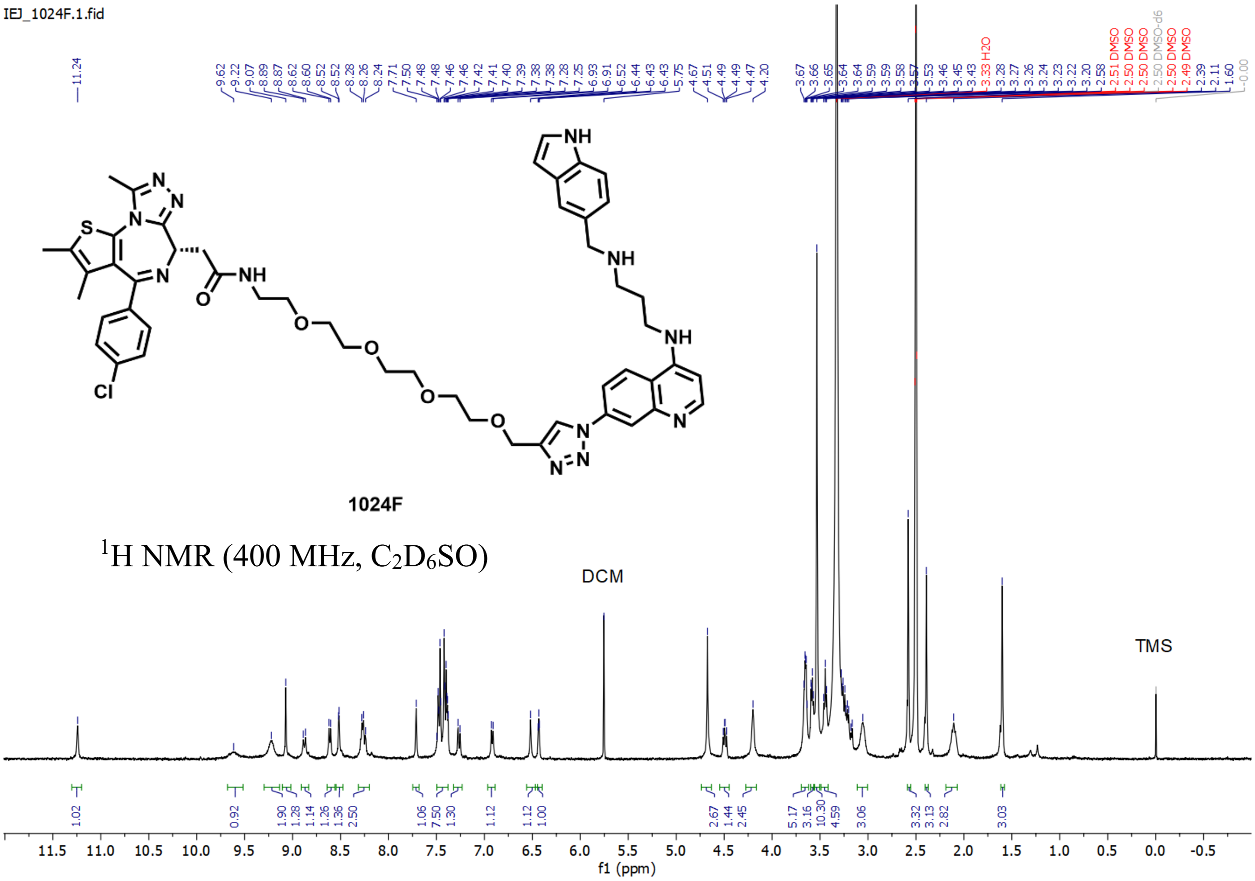
IEJ_1024F.1.fid
1024F
^{1}H NMR (400 MHz, C_2D_6SO)
DCM
TMS
f1 (ppm)

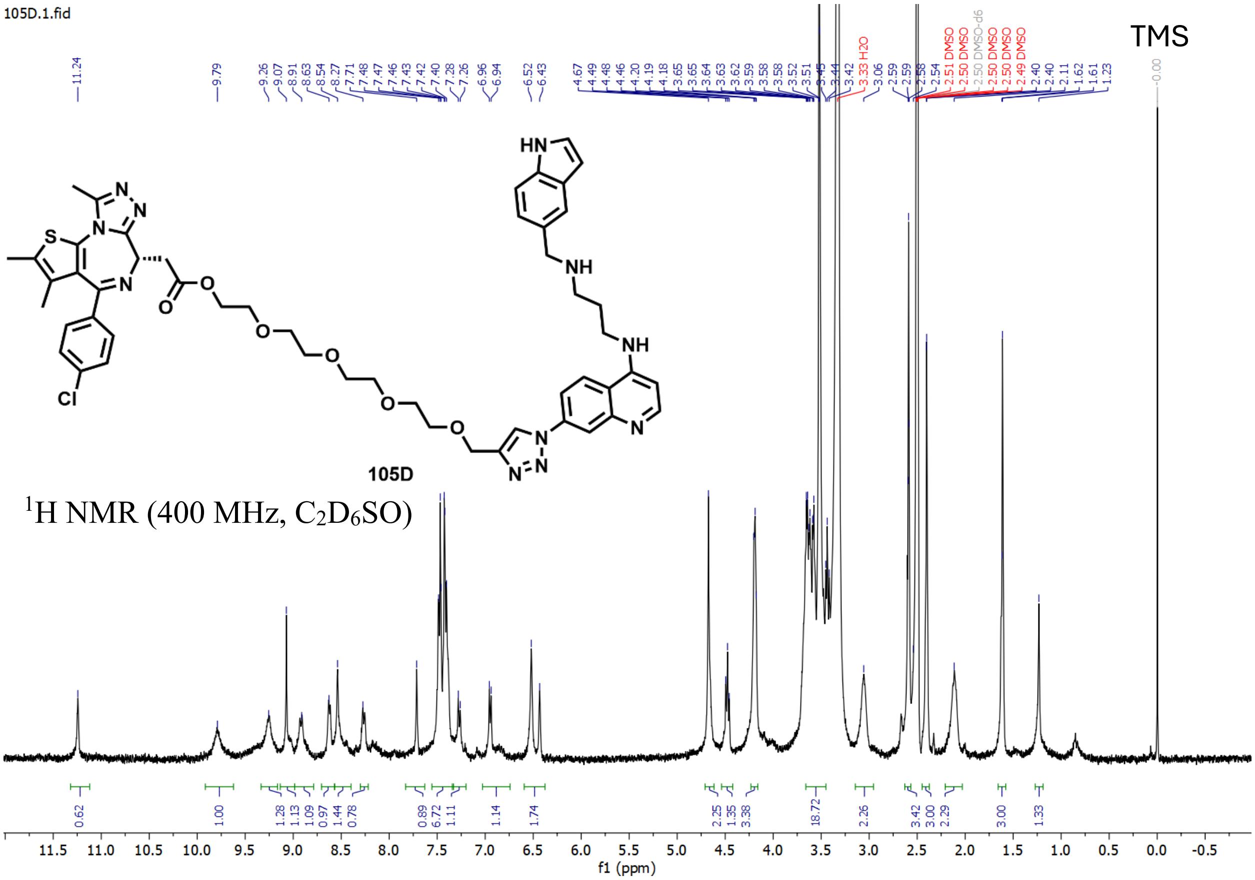
105D.1.fid
TMS
105D
^{1}H NMR (400 MHz, C_2D_6SO)
f1 (ppm)

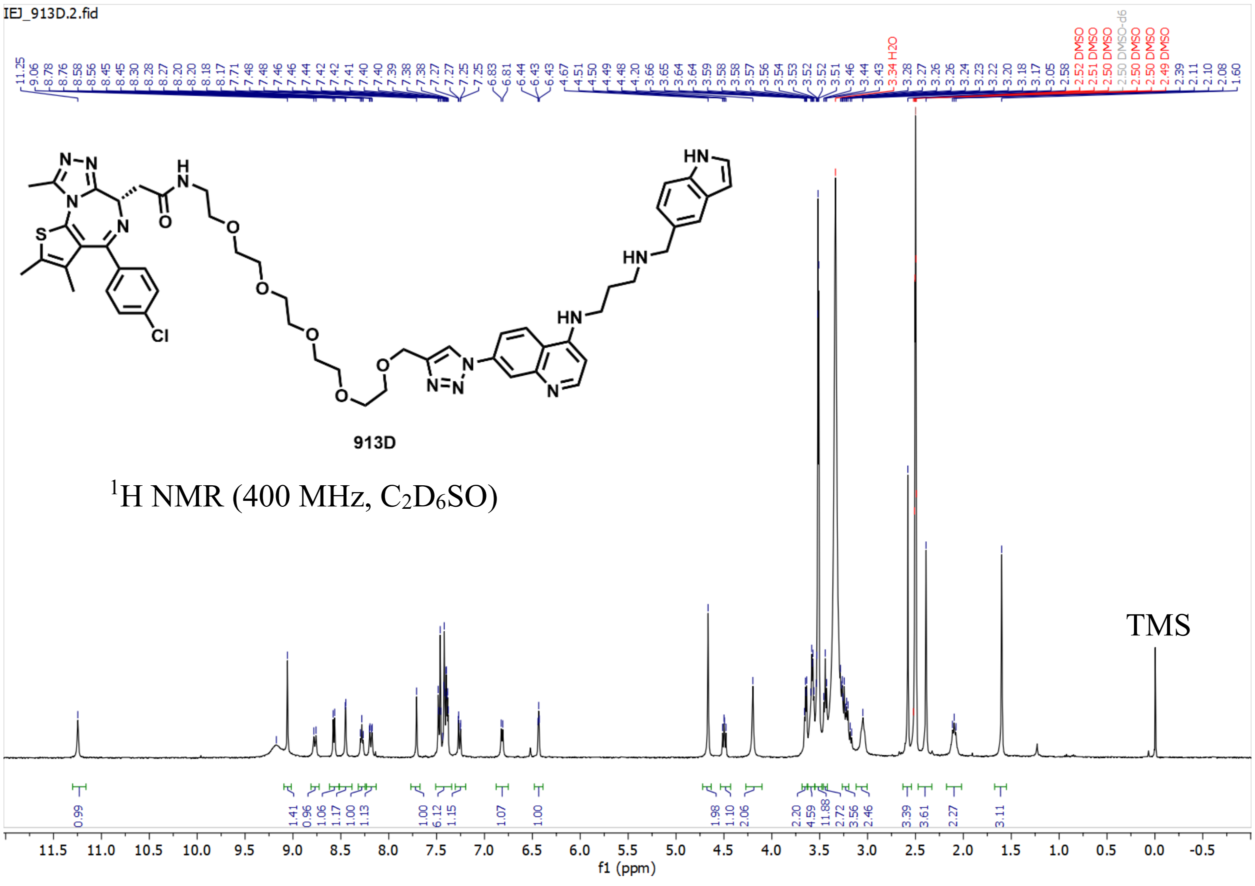
IEJ_913D.2.fid
913D
^{1}H NMR (400 MHz, C_2D_6SO)
TMS
f1 (ppm)

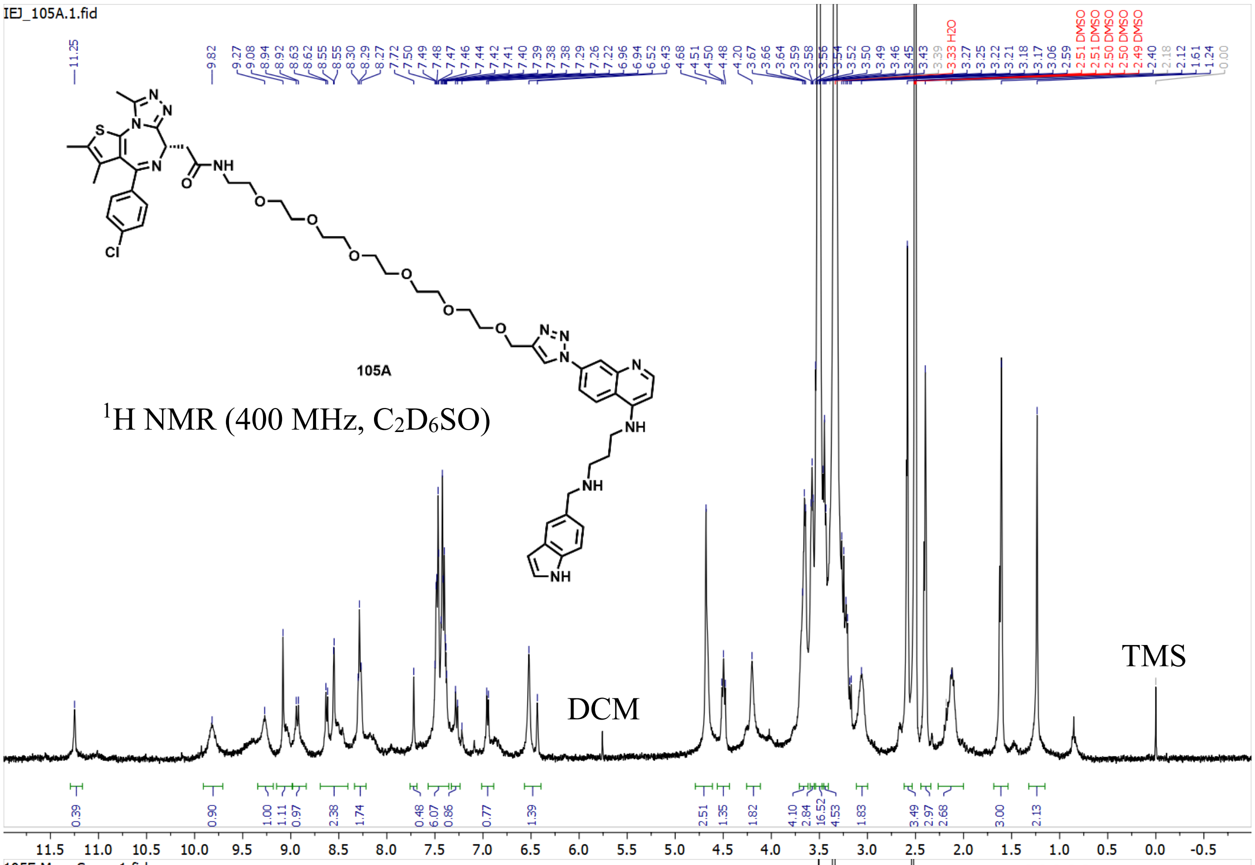
IEJ_105A.1.fid
105A
1H NMR (400 MHz, C2D6SO)
DCM
TMS
f1 (ppm)

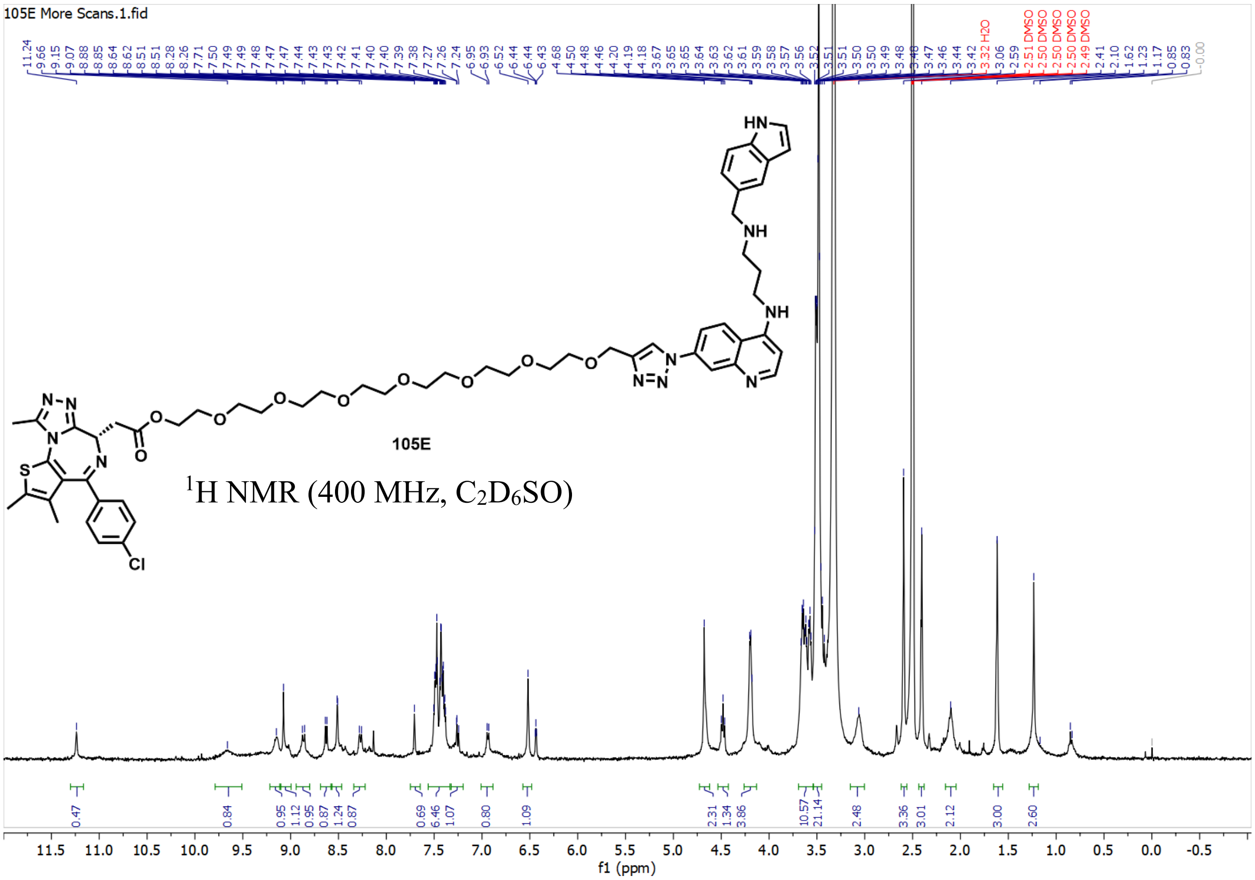
105E More Scans.1.fid
105E
1H NMR (400 MHz, C2D6SO)
f1 (ppm)

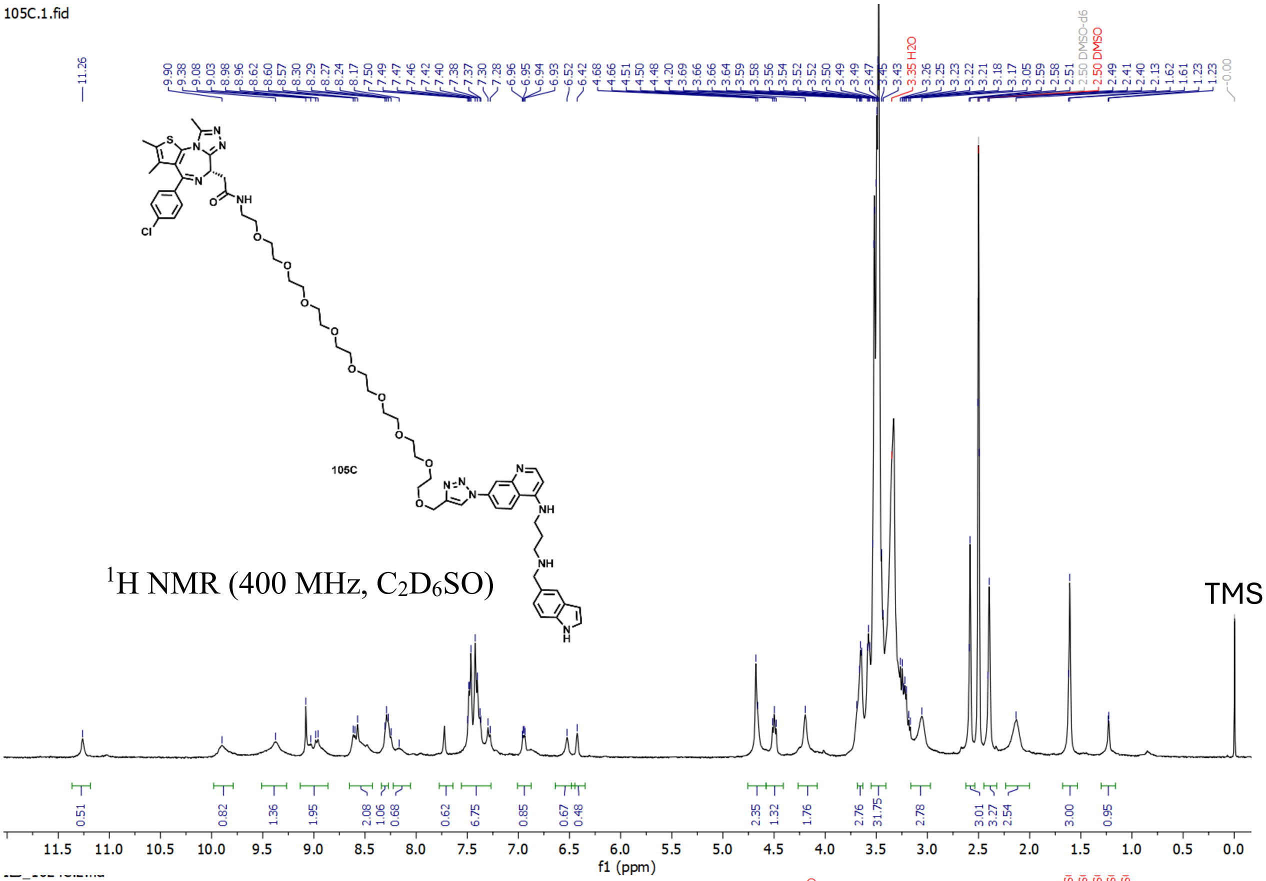
105C.1.fid
105C
1H NMR (400 MHz, C2D6SO)
TMS
f1 (ppm)

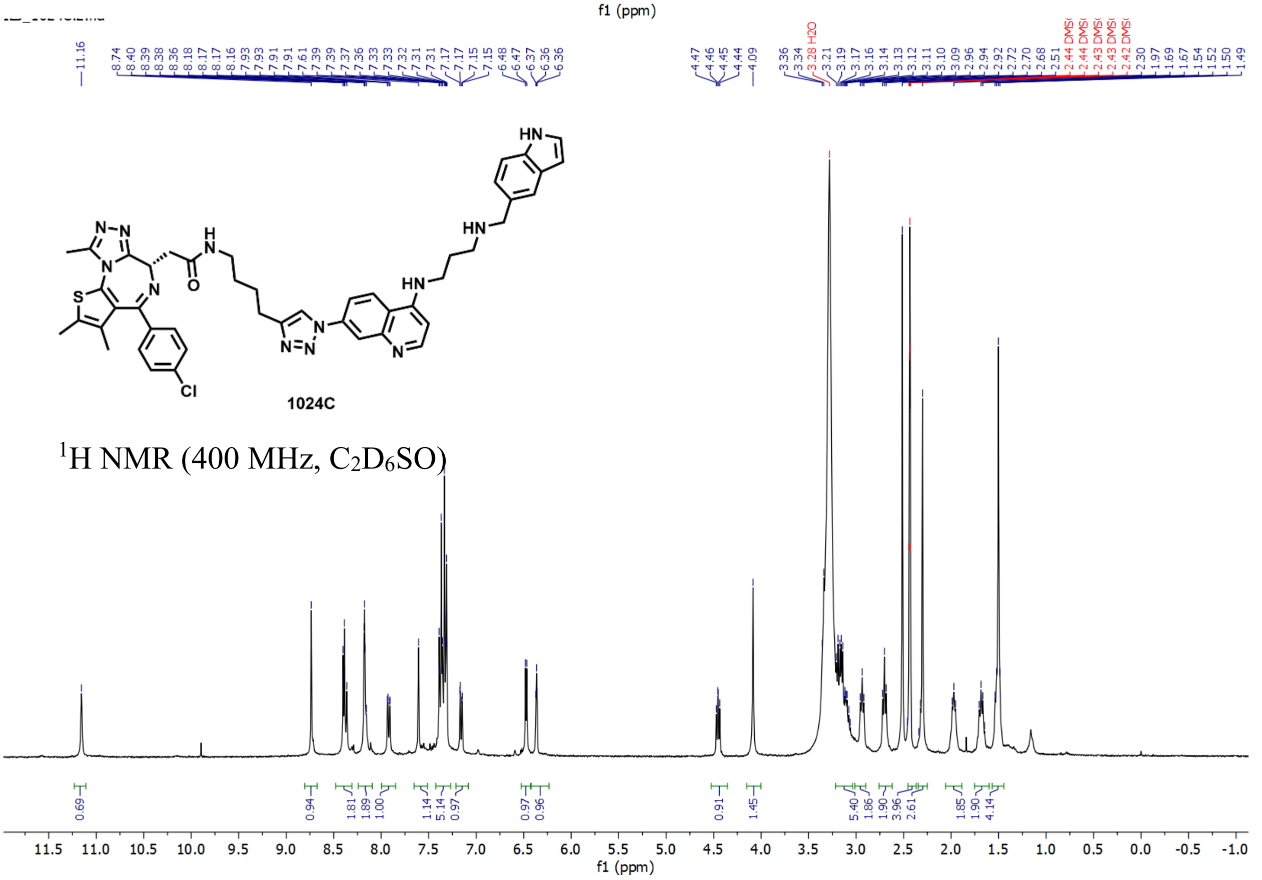
1024C
1H NMR (400 MHz, C2D6SO)
f1 (ppm)

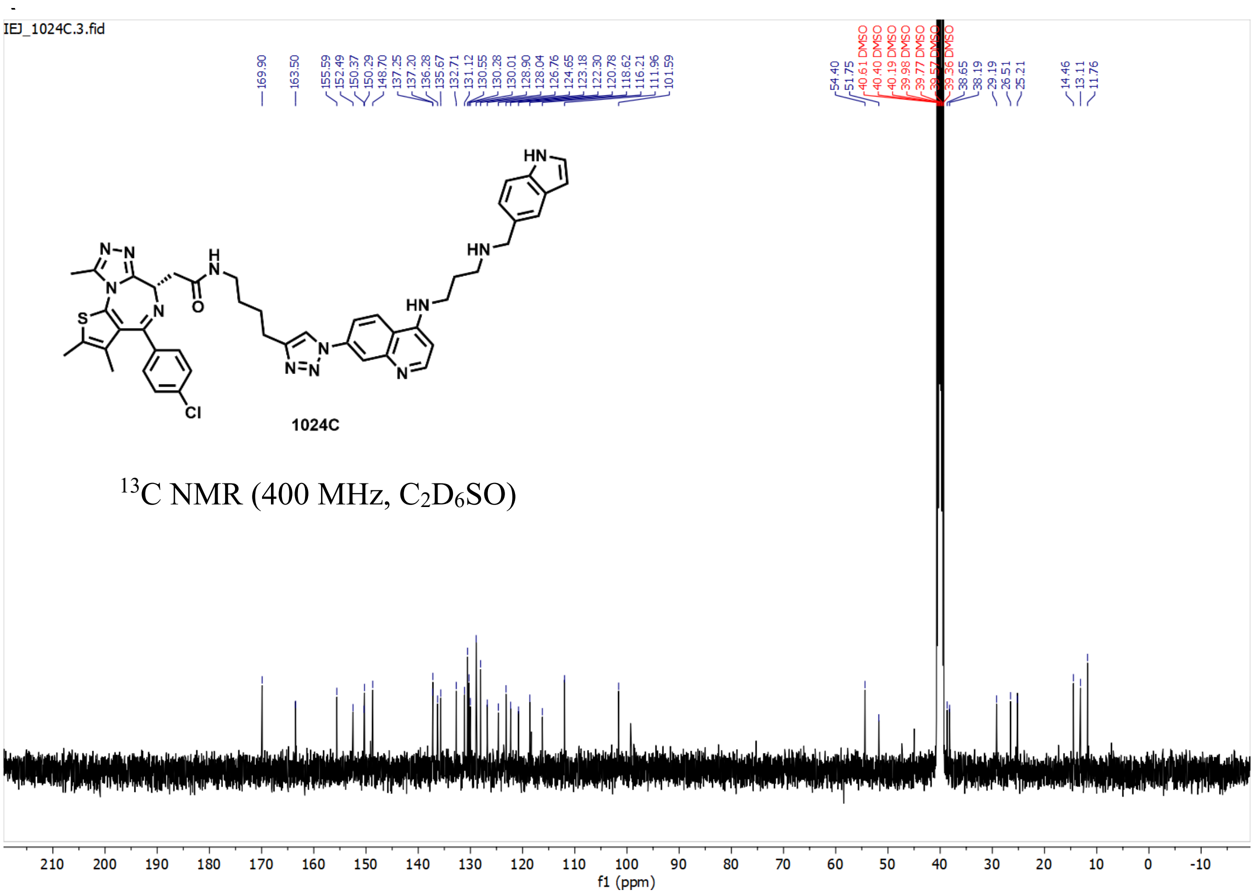

1024C

^{13}C NMR (400 MHz, C_2D_6SO)

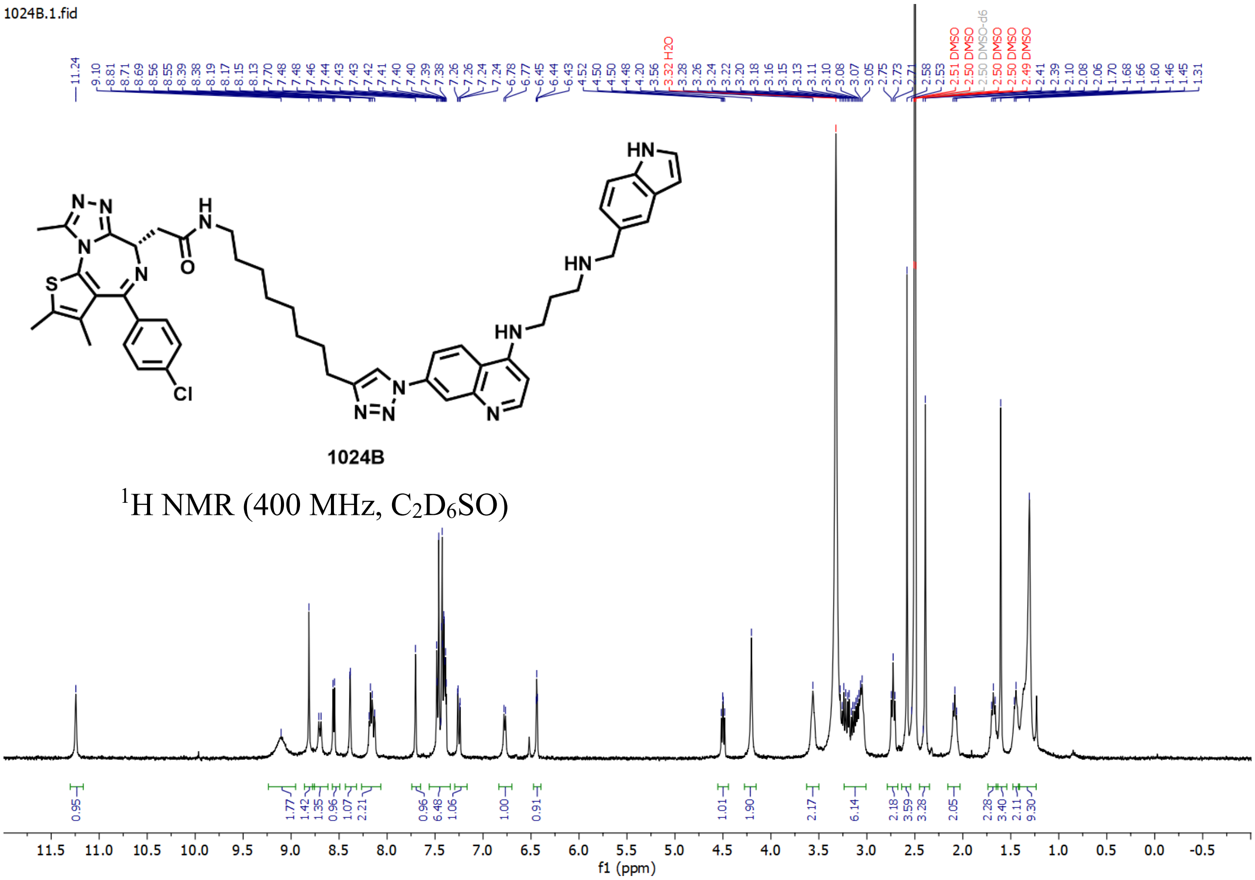

1024B

^{1}H NMR (400 MHz, C_2D_6SO)

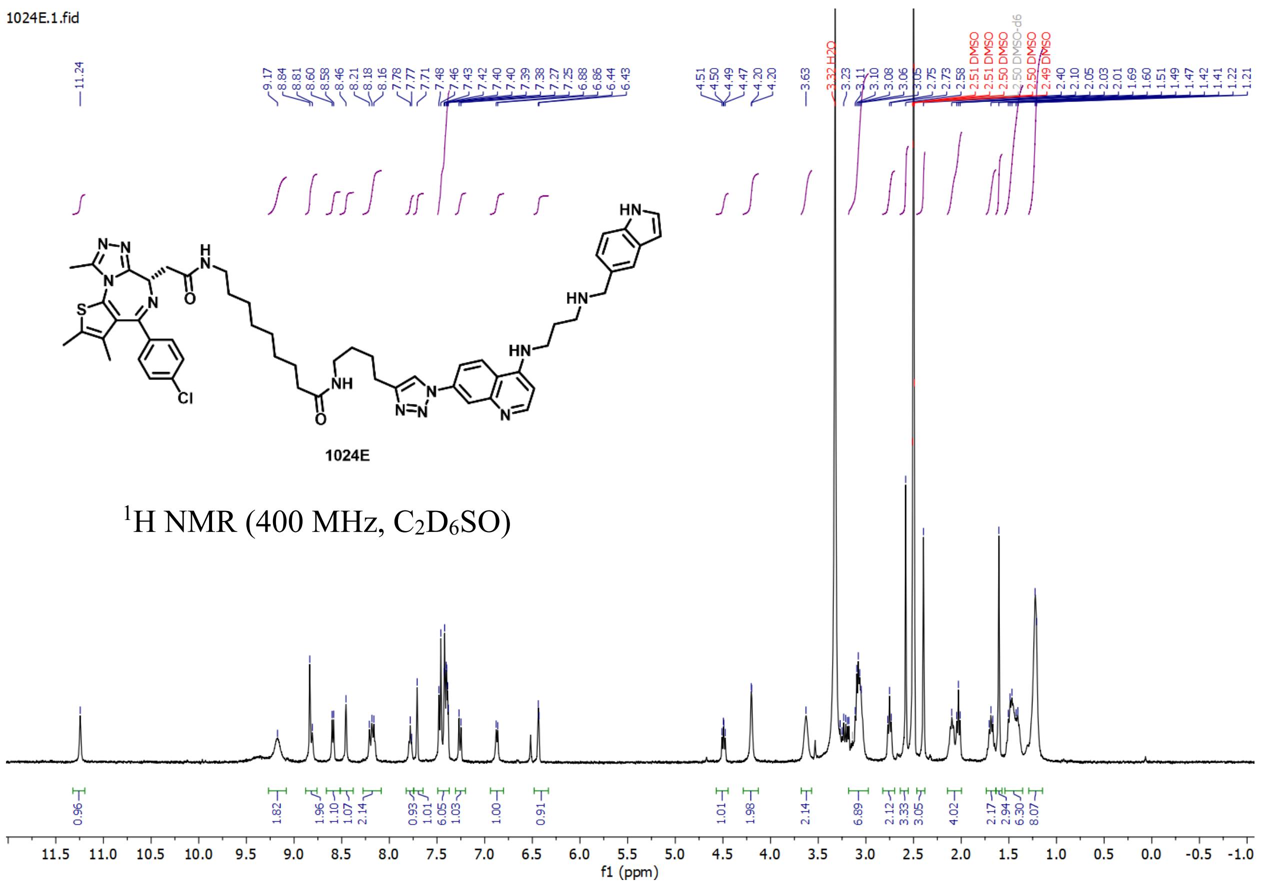
1024E.1.fid
1024E
^{1}H NMR (400 MHz, C_2D_6SO)
f1 (ppm)

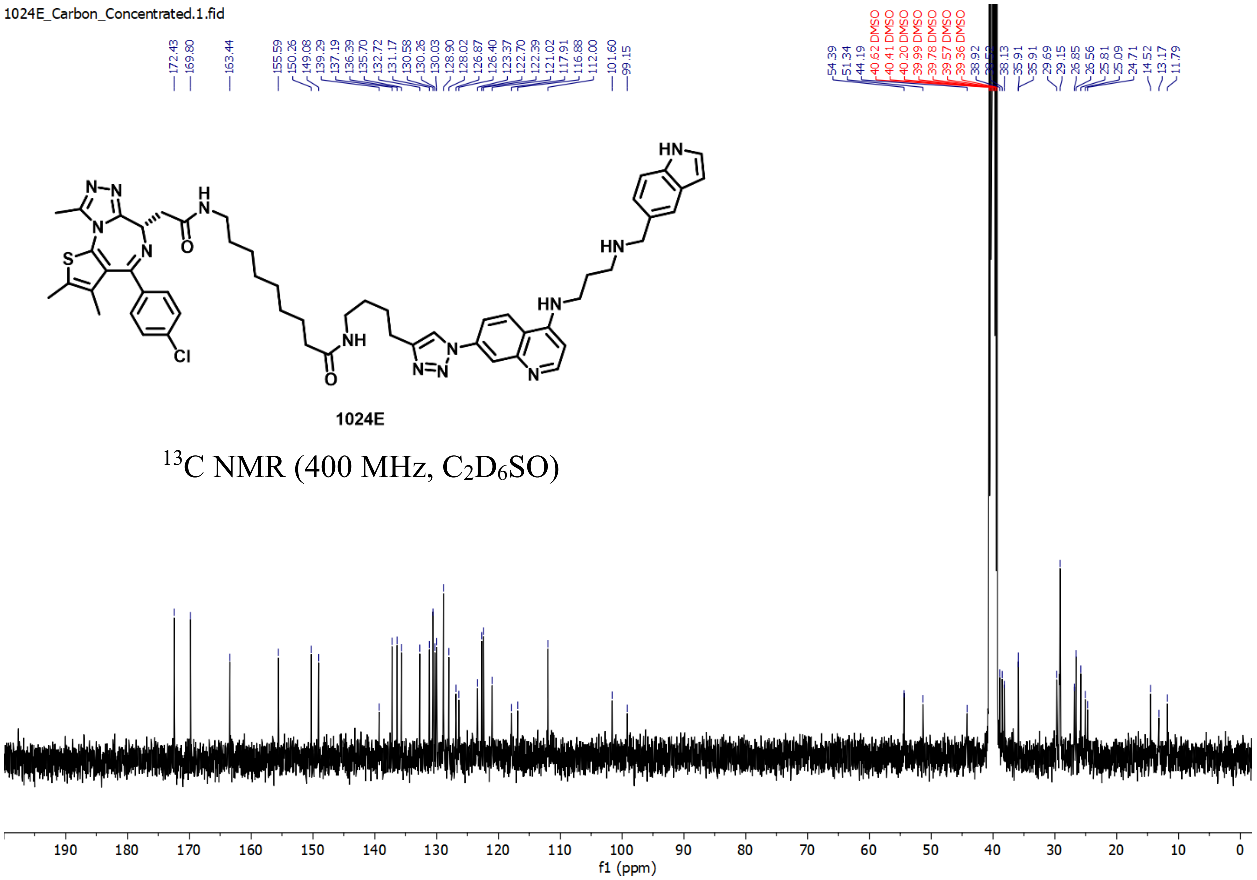
1024E_Carbon_Concentrated.1.fid
1024E
^{13}C NMR (400 MHz, C_2D_6SO)
f1 (ppm)

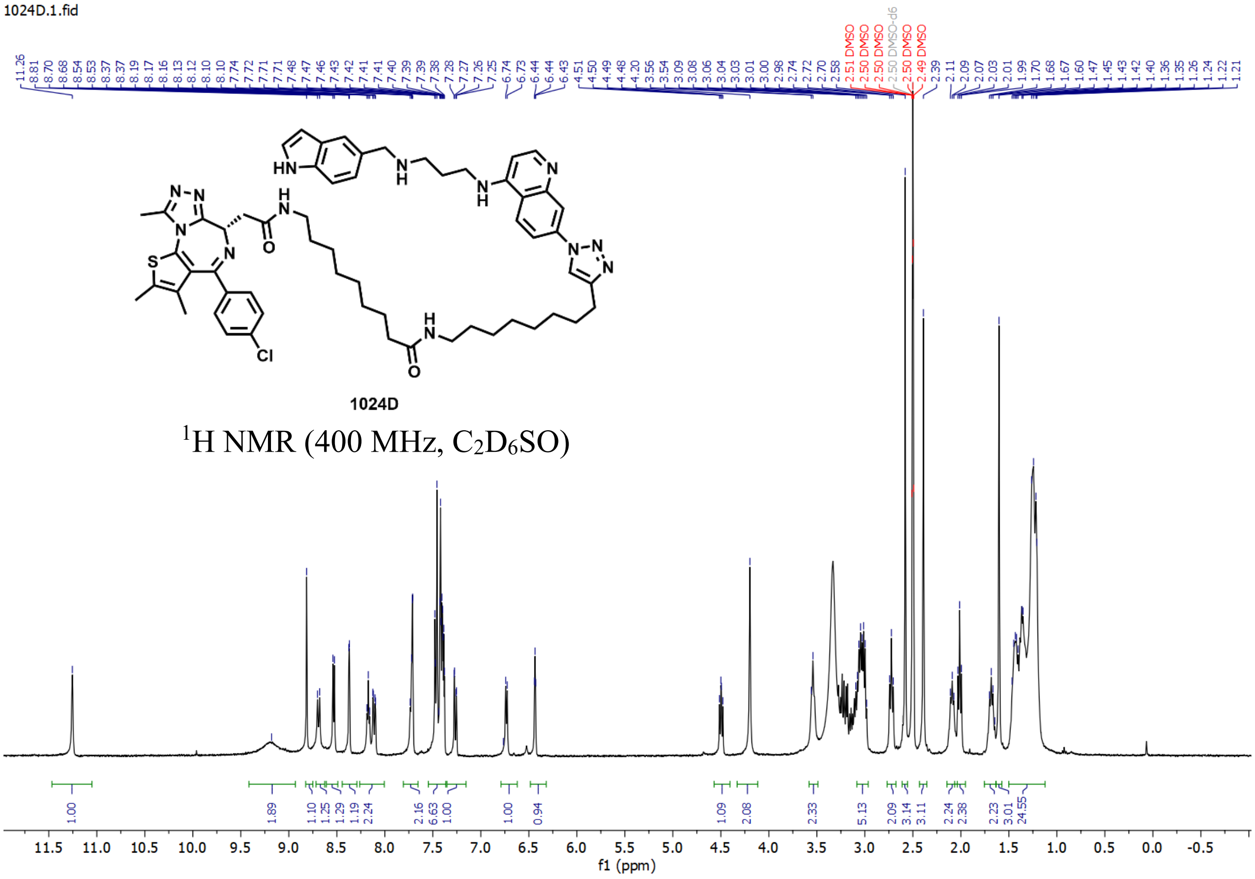
1024D.1.fid
1024D
^{1}H NMR (400 MHz, C_2D_6SO)
f1 (ppm)

HPLC

105B

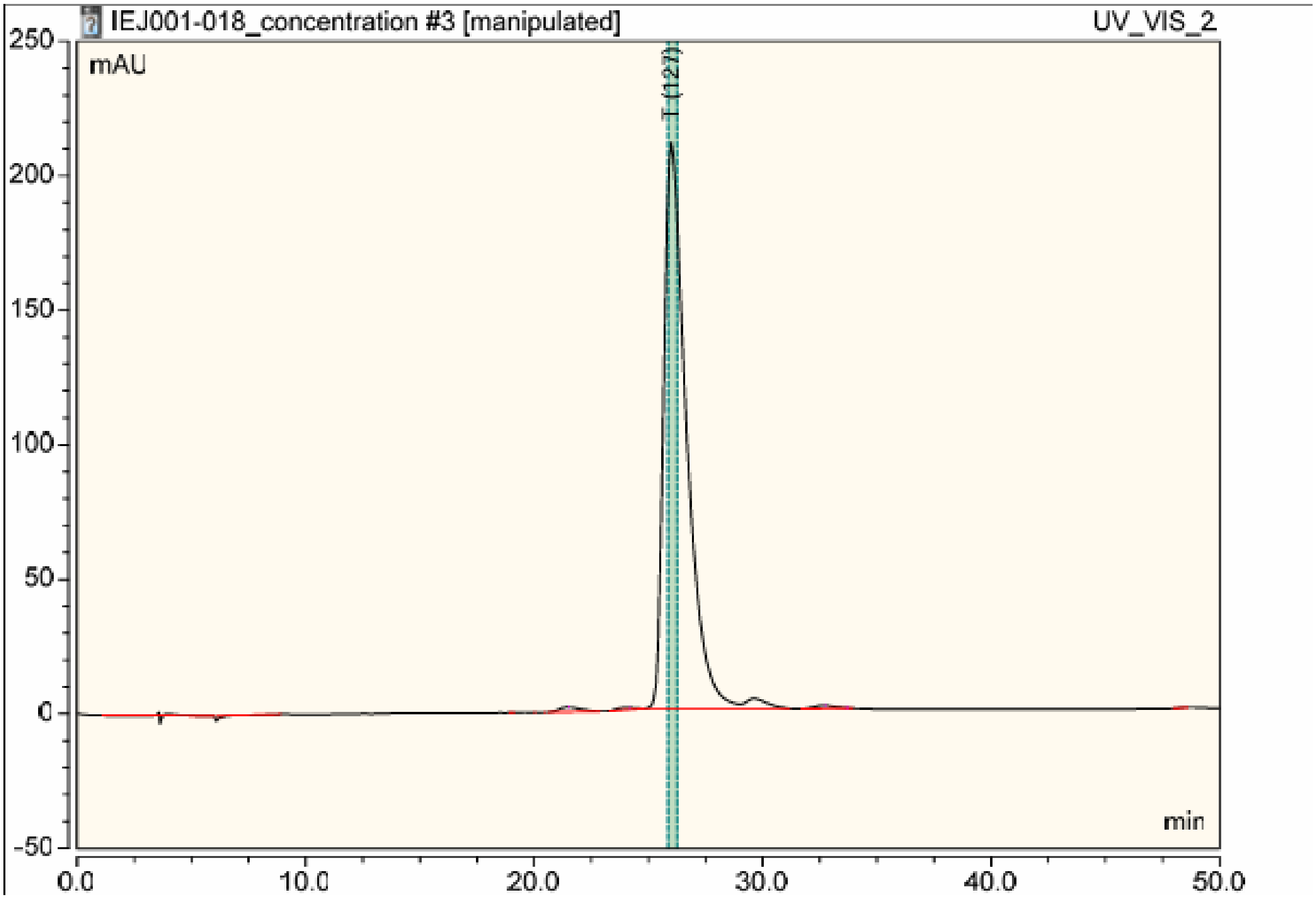

No.	Ret.Time min	Peak Name	Height mAU	Area mAU*min	Rel.Area %	Amount n.a.	Type
1	3.39		0.056	0.182	0.07	n.a.	BMb*
2	3.58		1.210	0.057	0.02	n.a.	bM *
3	3.90		0.456	0.040	0.02	n.a.	MB*
4	8.91		0.000	0.299	0.12	n.a.	MB*
5	19.06		0.031	0.005	0.00	n.a.	BMB
6	20.00		0.019	0.001	0.00	n.a.	BMB
7	20.50		0.013	0.001	0.00	n.a.	BMB
8	21.44		1.739	1.881	0.75	n.a.	BMB
9	21.48		0.010	0.000	0.00	n.a.	Rd
10	24.15		0.738	0.748	0.30	n.a.	BMb
11	24.18		0.008	0.000	0.00	n.a.	Rd
12	26.00		210.476	241.666	96.52	n.a.	bM
13	29.64		3.808	4.142	1.65	n.a.	MB
14	32.64		1.181	1.329	0.53	n.a.	BMB
15	32.71		0.011	0.000	0.00	n.a.	Rd
16	33.69		0.014	0.001	0.00	n.a.	Rd
17	48.59		0.035	0.030	0.01	n.a.	BMB
Total:			219.805	250.383	100.00	0.000	

SJ1008066

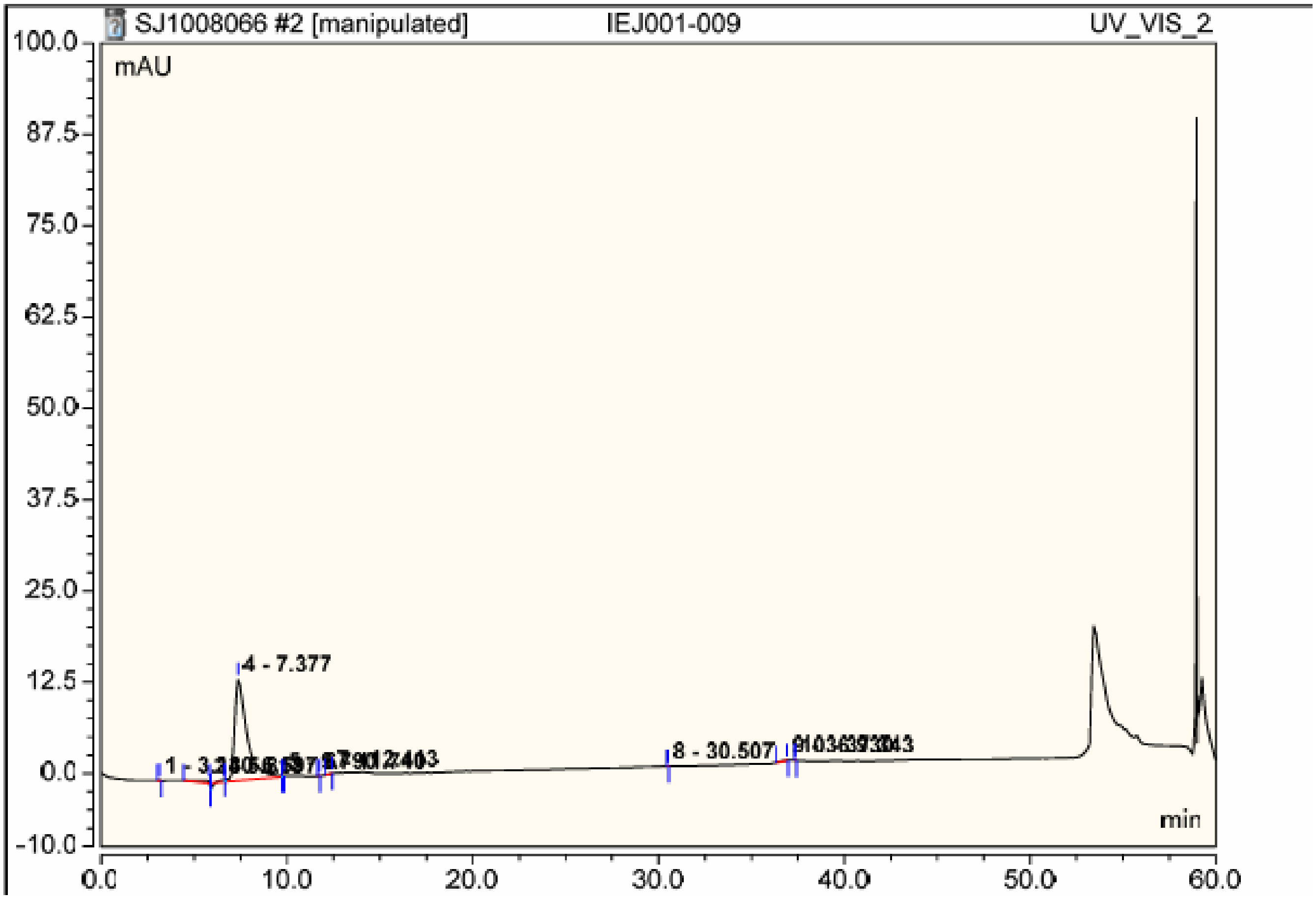

No.	Ret.Time min	Peak Name	Height mAU	Area mAU*min	Rel.Area %	Amount n.a.	Type
1	3.14		0.093	0.010	0.09	n.a.	BMB
2	5.81		0.417	0.227	2.05	n.a.	BM *
3	6.60		0.259	0.009	0.08	n.a.	M *
4	7.38		13.916	10.771	97.39	n.a.	M
5	9.79		0.107	0.011	0.10	n.a.	M *
6	11.74		0.015	0.001	0.01	n.a.	BMB
7	12.41		0.013	0.006	0.06	n.a.	BMB
8	30.51		0.017	0.001	0.01	n.a.	BMB
9	36.93		0.023	0.023	0.21	n.a.	BMB
10	37.34		0.018	0.001	0.01	n.a.	BMB
Total:			14.879	11.060	100.00	0.000	

908B

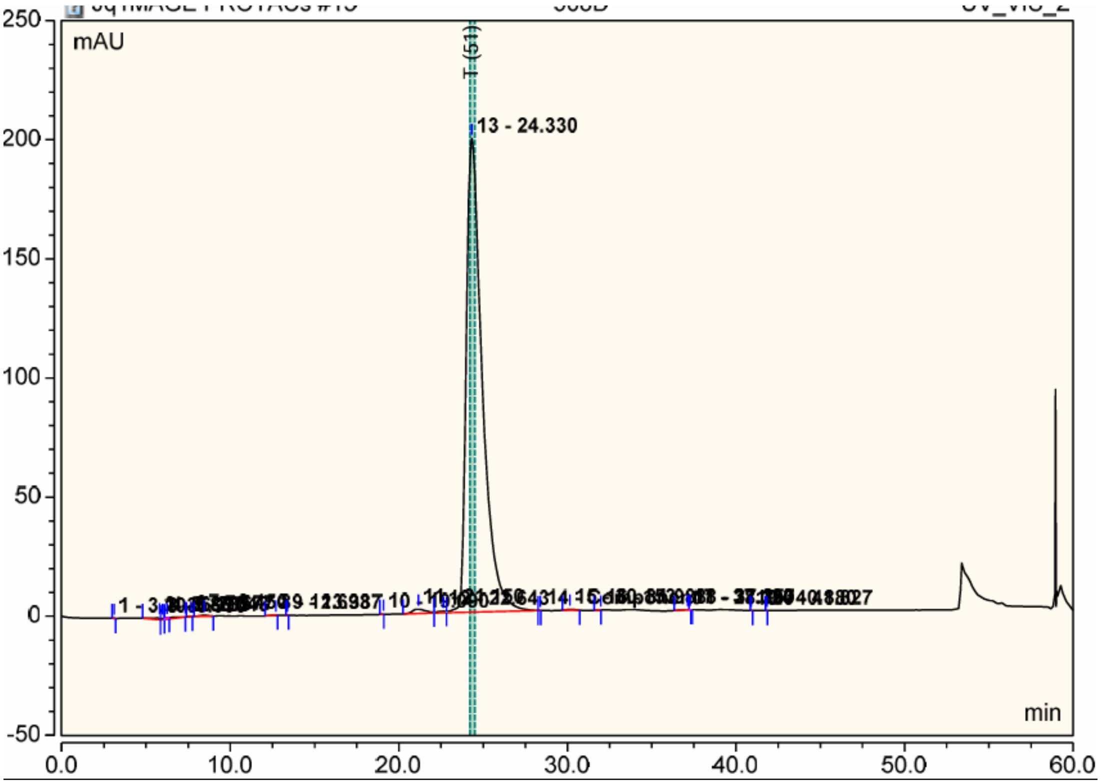

No.	Ret.Time min	Peak Name	Height mAU	Area mAU*min	Rel.Area %	Amount micromol	Type
1	3.10		0.091	0.010	0.00	n.a.	BMB
2	5.81		0.950	0.409	0.18	n.a.	BMB
3	5.99		0.567	0.098	0.04	n.a.	BM
4	6.23		0.197	0.036	0.02	n.a.	Ru
5	7.34		0.000	0.429	0.19	n.a.	MB
6	7.68		0.046	0.018	0.01	n.a.	BMB
7	8.45		0.457	0.236	0.11	n.a.	BMB
8	12.69		0.083	0.046	0.02	n.a.	BMB
9	13.39		0.017	0.001	0.00	n.a.	BMB
10	19.09		0.010	0.003	0.00	n.a.	BMB
11	21.15		1.809	1.703	0.77	n.a.	BM
12	22.64		0.649	0.406	0.18	n.a.	M
13	24.33		198.867	217.530	98.29	n.a.	M
14	28.39	Component 1	0.018	0.003	0.00	n.a.	MB
15	30.15		0.411	0.242	0.11	n.a.	BMB
16	31.98		0.011	0.005	0.00	n.a.	BMB
17	37.13		0.148	0.125	0.06	n.a.	BMB
18	37.26		0.020	0.001	0.00	n.a.	Rd
19	40.88		0.019	0.001	0.00	n.a.	BMB
20	41.83		0.018	0.001	0.00	n.a.	BMB
Total:			204.388	221.303	100.00	0.000	

913B

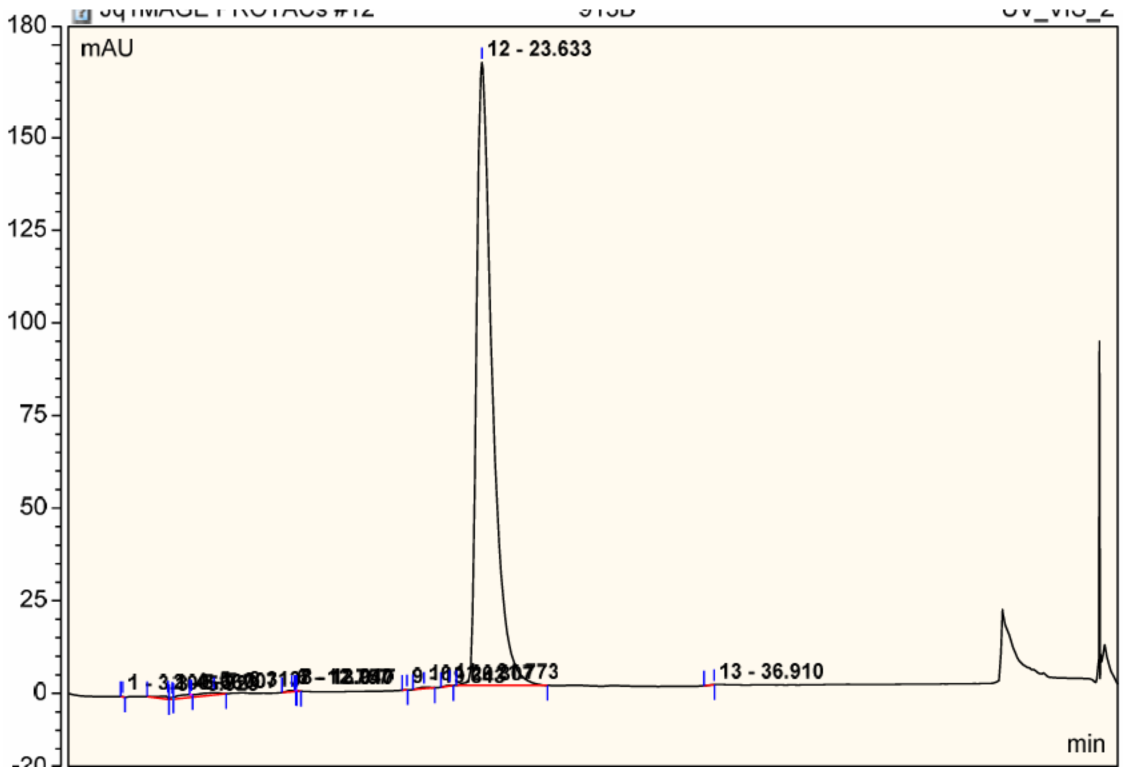

No.	Ret.Time min	Peak Name	Height mAU	Area mAU*min	Rel.Area %	Amount n.a.	Type
1	3.10		0.101	0.012	0.01	n.a.	BMB
2	5.67		0.917	0.525	0.29	n.a.	BMB
3	5.92		0.532	0.098	0.05	n.a.	BM
4	7.01		0.021	0.002	0.00	n.a.	Ru
5	8.31		0.629	1.860	1.03	n.a.	MB
6	12.76		0.409	0.257	0.14	n.a.	BMB
7	12.94		0.010	0.000	0.00	n.a.	Rd
8	13.02		0.011	0.000	0.00	n.a.	Rd
9	19.34		0.019	0.005	0.00	n.a.	BMB
10	20.31		0.406	0.289	0.16	n.a.	BMB
11	21.77		0.160	0.084	0.05	n.a.	BMB
12	23.63		168.153	177.247	98.26	n.a.	BMB
13	36.91		0.017	0.013	0.01	n.a.	BMB
Total:			171.385	180.393	100.00	0.000	

913C

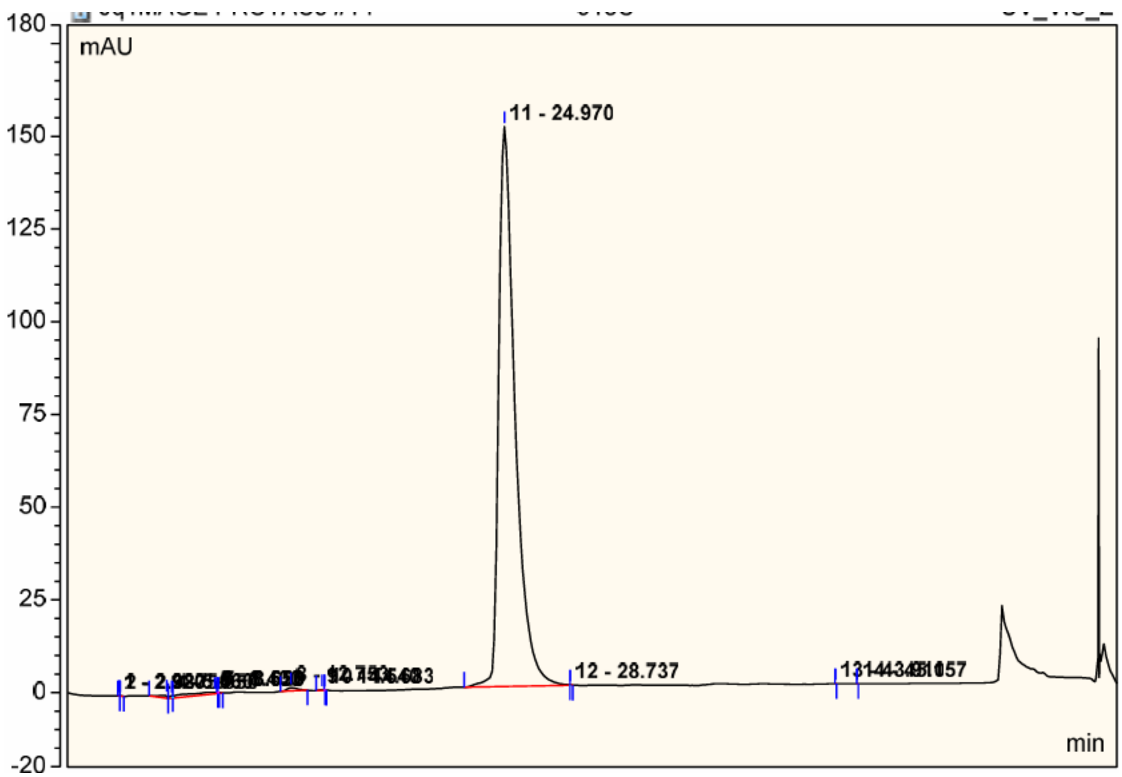

No.	Ret.Time min	Peak Name	Height mAU	Area mAU*min	Rel.Area %	Amount n.a.	Type
1	2.92		0.031	0.002	0.00	n.a.	BMb
2	2.99		0.013	0.012	0.01	n.a.	bMB
3	5.66		0.831	0.411	0.25	n.a.	BMB
4	5.95		0.582	0.125	0.07	n.a.	BM
5	8.40		0.398	1.496	0.89	n.a.	MB
6	8.55		0.017	0.000	0.00	n.a.	Rd
7	8.62		0.017	0.001	0.00	n.a.	Rd
8	12.75		0.847	0.615	0.37	n.a.	BMB
9	14.54		0.188	0.066	0.04	n.a.	BMB
10	14.68		0.015	0.000	0.00	n.a.	Rd
11	24.97		150.937	164.923	98.37	n.a.	BM
12	28.74		0.076	0.007	0.00	n.a.	MB
13	43.91		0.031	0.001	0.00	n.a.	BMB
14	45.16		0.027	0.001	0.00	n.a.	BMB
Total:			154.009	167.662	100.00	0.000	

1024F

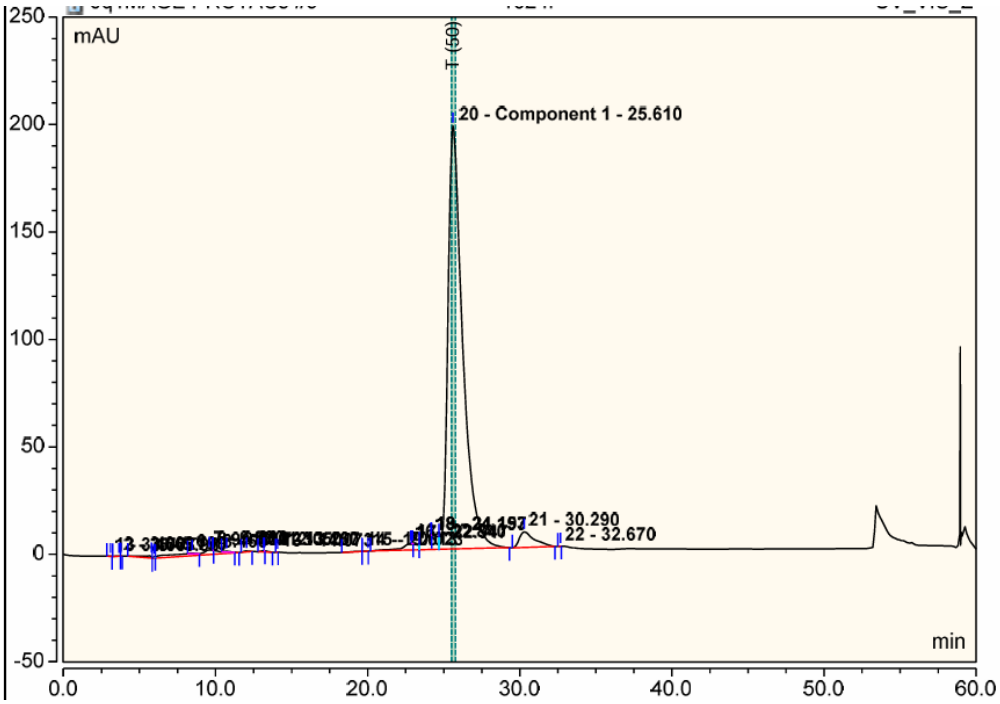

No.	Ret.Time min	Peak Name	Height mAU	Area mAU*min	Rel.Area %	Amount micromol	Type
1	3.10		0.157	0.030	0.01	n.a.	BMB
2	3.66		0.096	0.031	0.01	n.a.	BM
3	3.78		0.055	0.003	0.00	n.a.	MB
4	5.79		1.115	0.832	0.34	n.a.	BMB
5	5.97		0.652	0.085	0.03	n.a.	BM
6	8.46		0.319	0.125	0.05	n.a.	Ru
7	9.59		2.137	5.414	2.21	n.a.	MB
8	9.83		0.008	0.000	0.00	n.a.	Rd
9	10.73		0.359	0.160	0.07	n.a.	Rd
10	12.05		0.432	0.176	0.07	n.a.	BMB
11	13.16		0.504	0.258	0.11	n.a.	BMB
12	13.21		0.011	0.001	0.00	n.a.	Rd
13	14.07		0.021	0.002	0.00	n.a.	BMB
14	19.61		0.046	0.086	0.04	n.a.	BM
15	20.02		0.009	0.002	0.00	n.a.	MB
16	22.84		3.030	3.618	1.48	n.a.	BM
17	22.95		0.022	0.002	0.00	n.a.	Rd
18	24.15		6.631	6.736	2.75	n.a.	M
19	24.20		0.007	0.000	0.00	n.a.	Rd
20	25.61	Component 1	196.857	218.698	89.42	n.a.	MB
21	30.29		7.269	8.319	3.40	n.a.	BMB
22	32.67		0.040	0.006	0.00	n.a.	BMB
Total:			219.778	244.584	100.00	0.000	

105D

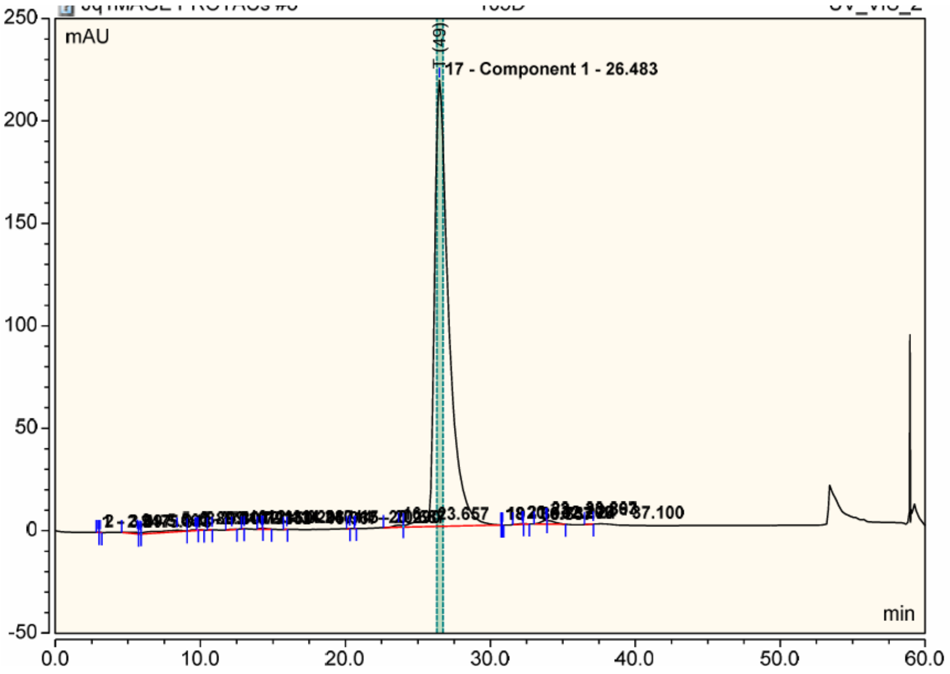

No.	Ret.Time min	Peak Name	Height mAU	Area mAU*min	Rel.Area %	Amount micromol	Type
1	2.95		0.028	0.002	0.00	n.a.	BMb
2	3.10		0.088	0.010	0.00	n.a.	bMB
3	5.66		0.906	0.482	0.19	n.a.	BMB
4	5.84		0.496	0.061	0.02	n.a.	BM
5	8.37		0.809	2.226	0.88	n.a.	M
6	9.64		0.468	0.377	0.15	n.a.	MB
7	9.81		0.015	0.001	0.00	n.a.	Rd
8	10.79		0.032	0.012	0.00	n.a.	BMB
9	12.15		0.202	0.086	0.03	n.a.	BMB
10	13.02		0.000	0.002	0.00	n.a.	BMB
11	14.24		0.497	0.270	0.11	n.a.	BMB
12	14.29		0.004	0.000	0.00	n.a.	Rd
13	15.97		0.030	0.007	0.00	n.a.	BMB
14	20.29		0.017	0.003	0.00	n.a.	BMB
15	20.69		0.023	0.001	0.00	n.a.	BMB
16	23.66		1.057	0.749	0.30	n.a.	BM
17	26.48	Component 1	217.727	247.478	97.47	n.a.	M
18	30.76		0.069	0.004	0.00	n.a.	M
19	30.84		0.046	0.003	0.00	n.a.	MB
20	32.20		0.309	0.211	0.08	n.a.	BMB
21	32.27		0.006	0.000	0.00	n.a.	Rd
22	33.87		1.817	1.886	0.74	n.a.	BMB
23	33.89		0.009	0.000	0.00	n.a.	Rd
24	37.10		0.013	0.020	0.01	n.a.	BMB
**Total:**			224.670	253.891	100.00	0.000	

913D

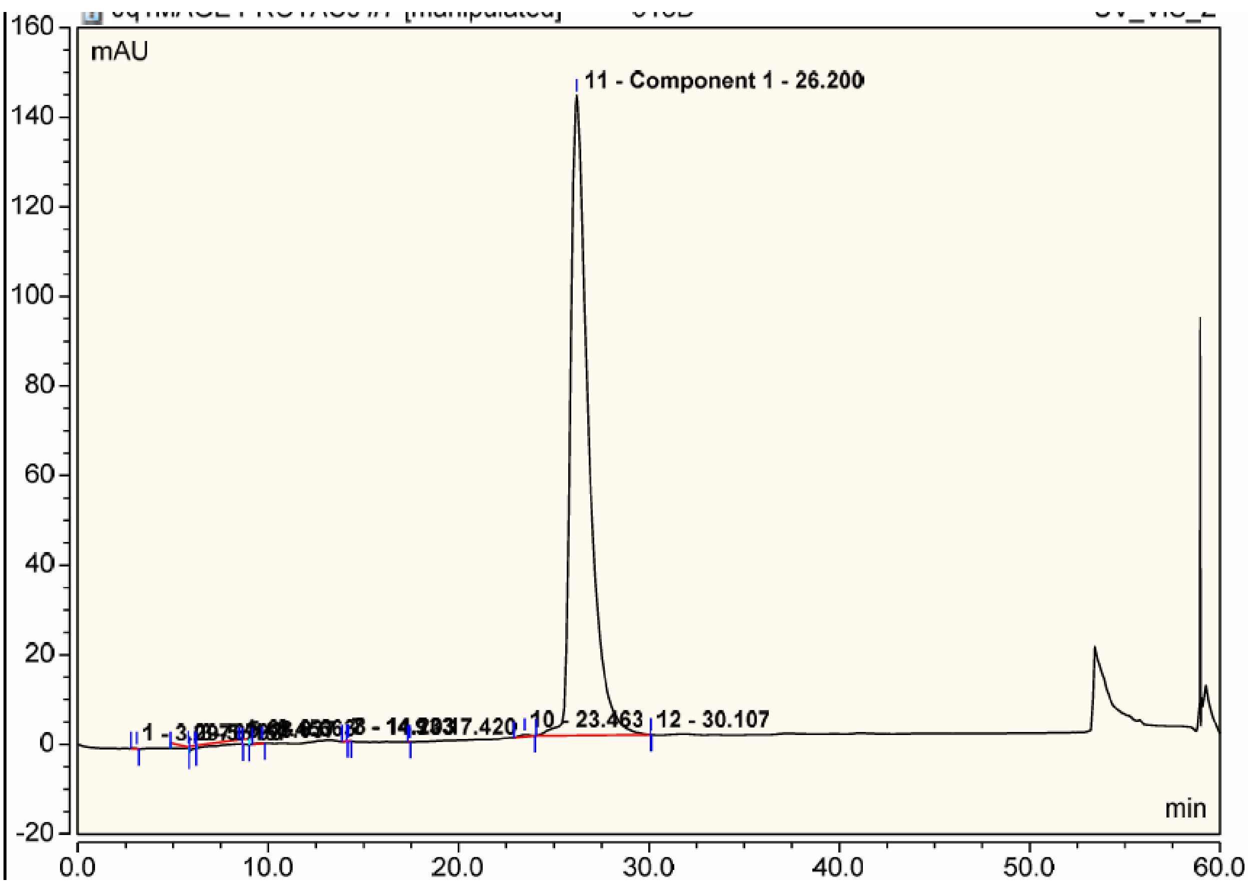

No.	Ret.Time min	Peak Name	Height mAU	Area mAU*min	Rel.Area %	Amount micromol	Type
1	3.10		0.151	0.034	0.02	n.a.	BMB
2	5.79		0.141	0.718	0.45	n.a.	M *
3	6.16		0.497	0.262	0.16	n.a.	M *
4	8.45		0.875	2.086	1.30	n.a.	M *
5	8.64		0.015	0.001	0.00	n.a.	Rd
6	9.66		0.081	0.048	0.03	n.a.	BMB
7	14.13		0.010	0.000	0.00	n.a.	Ru
8	14.20		0.083	0.021	0.01	n.a.	BMB
9	17.42		0.023	0.002	0.00	n.a.	BMB
10	23.46		0.445	0.268	0.17	n.a.	BMB
11	26.20	Component 1	142.813	156.517	97.85	n.a.	BM
12	30.11		0.030	0.001	0.00	n.a.	MB
Total:			145.164	159.958	100.00	0.000	

105A

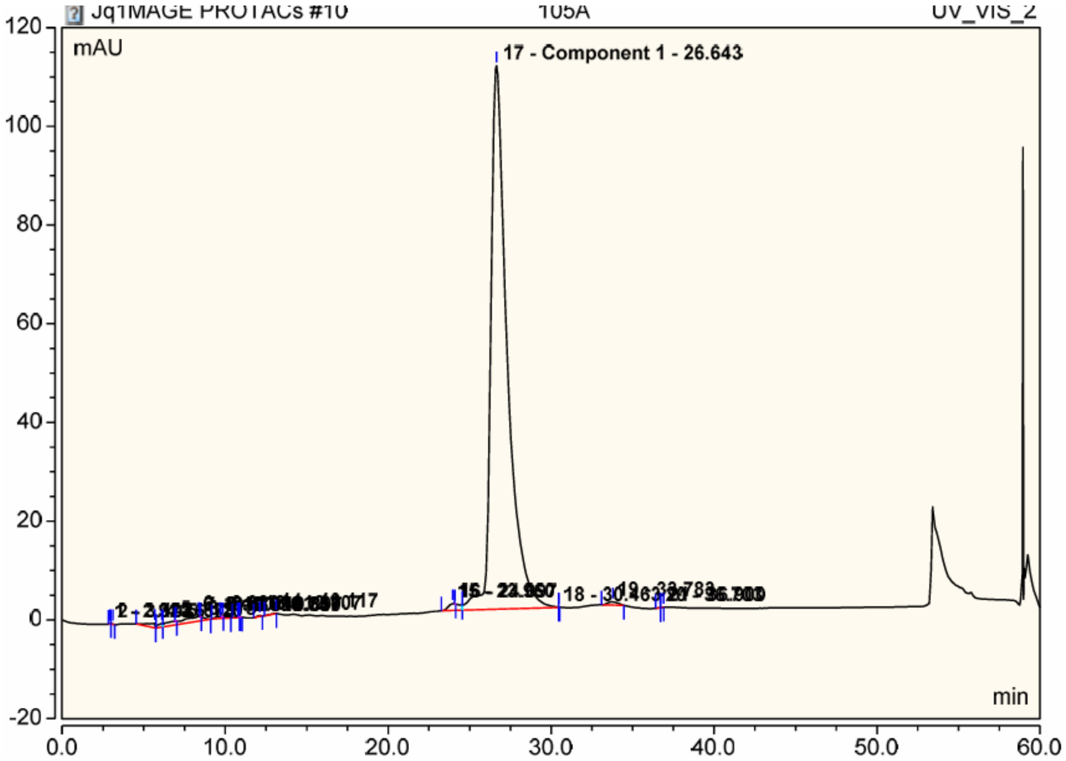

No.	Ret.Time min	Peak Name	Height mAU	Area mAU*min	Rel.Area %	Amount micromol	Type
1	2.92		0.045	0.003	0.00	n.a.	BMb
2	3.10		0.118	0.013	0.01	n.a.	bMB
3	5.67		0.906	0.508	0.38	n.a.	BMB
4	6.12		0.746	0.238	0.18	n.a.	BM
5	6.97		0.016	0.001	0.00	n.a.	Ru
6	8.38		0.915	1.943	1.46	n.a.	MB
7	8.47		0.021	0.001	0.00	n.a.	Rd
8	9.69		0.377	0.273	0.21	n.a.	BMB
9	9.84		0.021	0.002	0.00	n.a.	Rd
10	10.76		0.168	0.056	0.04	n.a.	BMB
11	10.84		0.018	0.001	0.00	n.a.	Rd
12	10.90		0.013	0.000	0.00	n.a.	Rd
13	12.11		0.179	0.062	0.05	n.a.	BMB
14	13.12		0.035	0.041	0.03	n.a.	BMB
15	23.96		1.392	1.126	0.84	n.a.	BM
16	24.10		0.027	0.001	0.00	n.a.	Rd
17	26.64	Component 1	110.114	128.567	96.44	n.a.	M
18	30.46		0.028	0.001	0.00	n.a.	MB
19	33.78		0.641	0.469	0.35	n.a.	BMB
20	36.70		0.013	0.004	0.00	n.a.	BM
21	36.90		0.016	0.002	0.00	n.a.	MB
Total:			115.811	133.313	100.00	0.000	

105E

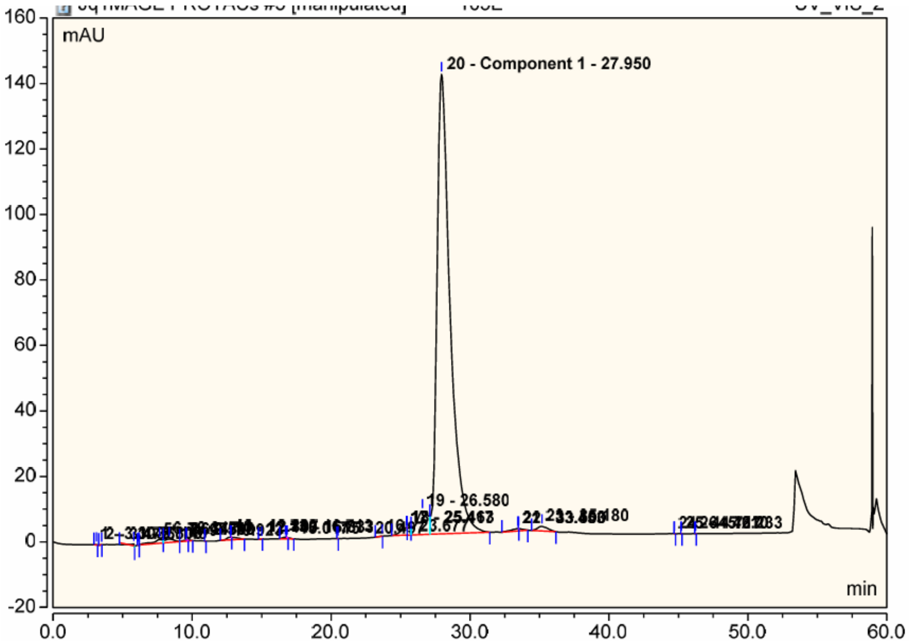

No.	Ret.Time min	Peak Name	Height mAU	Area mAU*min	Rel.Area %	Amount micromol	Type
1	3.10		0.172	0.021	0.01	n.a.	BMB
2	3.48		0.011	0.004	0.00	n.a.	BMB
3	5.80		0.539	0.101	0.06	n.a.	M *
4	6.14		0.194	0.011	0.01	n.a.	BM *
5	7.65		1.158	0.997	0.60	n.a.	M
6	8.35		1.055	0.830	0.50	n.a.	M
7	9.54		0.317	0.198	0.12	n.a.	MB
8	9.59		0.019	0.002	0.00	n.a.	Rd
9	10.92		0.023	0.001	0.00	n.a.	BMB
10	12.73		0.836	0.691	0.41	n.a.	BMB
11	12.81		0.015	0.001	0.00	n.a.	Rd
12	15.02		0.041	0.010	0.01	n.a.	BMB
13	16.71		0.459	0.241	0.14	n.a.	BMB
14	16.83		0.027	0.003	0.00	n.a.	Rd
15	20.50		0.018	0.001	0.00	n.a.	BMB
16	23.68		0.020	0.024	0.01	n.a.	BMB
17	25.42		2.212	1.763	1.05	n.a.	BM
18	25.46		0.012	0.000	0.00	n.a.	Rd
19	26.58		7.195	6.795	4.06	n.a.	M
20	27.95	Component 1	140.362	153.554	91.85	n.a.	MB
21	33.45		0.749	0.715	0.43	n.a.	BMB
22	33.50		0.004	0.000	0.00	n.a.	Rd
23	35.18		1.400	1.218	0.73	n.a.	BMB
24	44.72		0.020	0.001	0.00	n.a.	BMB
25	45.21		0.022	0.001	0.00	n.a.	BMB
26	46.23		0.020	0.001	0.00	n.a.	BMB
**Total:**			156.899	167.185	100.00	0.000	

105B

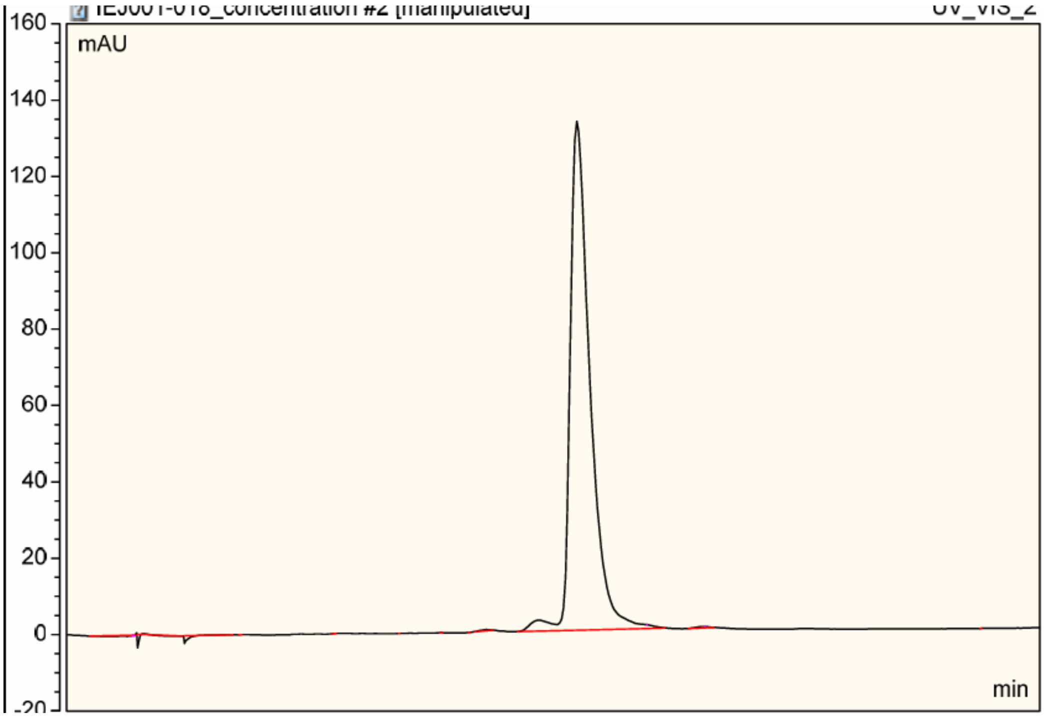

No.	Ret.Time min	Peak Name	Height mAU	Area mAU*min	Rel.Area %	Amount n.a.	Type
1	3.40		0.095	0.010	0.01	n.a.	Ru
2	3.58		0.848	0.447	0.28	n.a.	BM *
3	3.89		0.187	0.335	0.21	n.a.	MB*
4	8.16		0.105	0.281	0.17	n.a.	M *
5	8.42		0.049	0.004	0.00	n.a.	MB*
6	8.99		0.013	0.002	0.00	n.a.	BMB
7	13.79		0.036	0.006	0.00	n.a.	BMB
8	17.07		0.019	0.001	0.00	n.a.	BMB
9	19.29		0.019	0.004	0.00	n.a.	BMB
10	20.71		0.012	0.002	0.00	n.a.	BMB
11	21.54		0.345	0.197	0.12	n.a.	BMB
12	24.30		2.848	3.543	2.21	n.a.	BM
13	26.20		133.290	154.698	96.45	n.a.	M
14	29.67		1.084	0.572	0.36	n.a.	MB
15	29.80		0.015	0.001	0.00	n.a.	Rd
16	32.68		0.380	0.290	0.18	n.a.	BMB
17	32.78		0.011	0.000	0.00	n.a.	Rd
18	46.97		0.018	0.001	0.00	n.a.	BMB
Total:			139.374	160.391	100.00	0.000	

105C

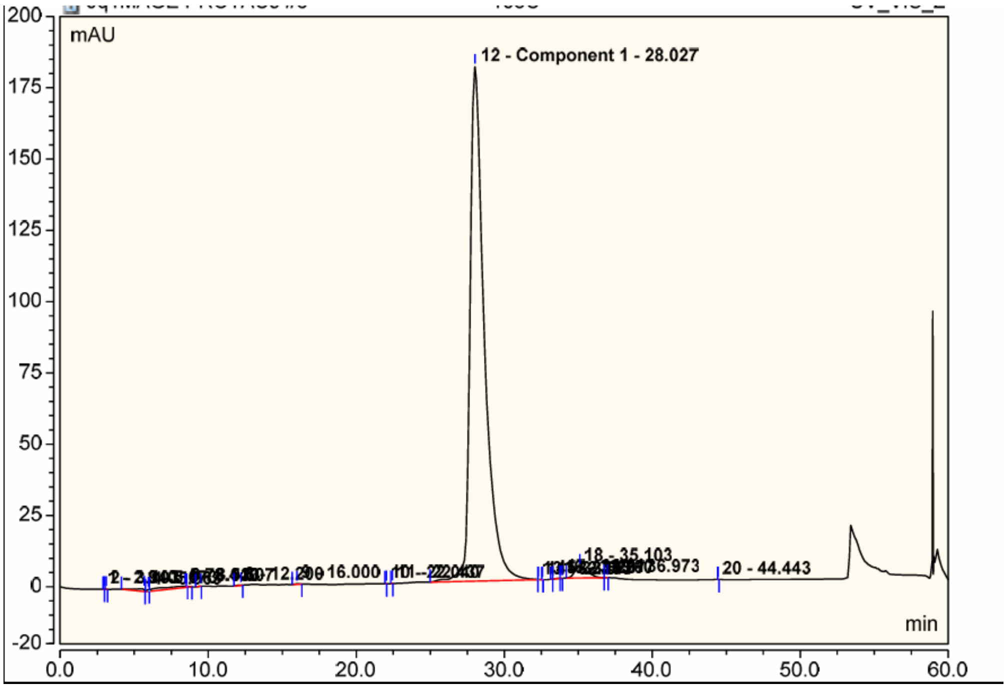

No.	Ret.Time min	Peak Name	Height mAU	Area mAU*min	Rel.Area %	Amount micromol	Type
1	2.94		0.027	0.001	0.00	n.a.	BMB
2	3.10		0.085	0.009	0.00	n.a.	BMB
3	5.67		0.988	0.730	0.35	n.a.	BMB
4	5.96		0.590	0.117	0.06	n.a.	BM
5	8.44		0.480	1.707	0.82	n.a.	MB
6	8.53		0.020	0.001	0.00	n.a.	Rd
7	9.51		0.022	0.008	0.00	n.a.	BMB
8	12.20		0.097	0.050	0.02	n.a.	BMB
9	16.00		0.215	0.083	0.04	n.a.	BMB
10	22.04		0.021	0.001	0.00	n.a.	BMB
11	22.44		0.020	0.002	0.00	n.a.	BMB
12	28.03	Component 1	180.393	202.740	96.80	n.a.	BM
13	32.29		0.118	0.019	0.01	n.a.	MB
14	32.58		0.018	0.001	0.00	n.a.	Rd
15	33.27		0.046	0.003	0.00	n.a.	BM
16	33.76		0.077	0.039	0.02	n.a.	M
17	33.92		0.026	0.006	0.00	n.a.	MB
18	35.10		3.905	3.928	1.88	n.a.	BM
19	36.97		0.013	0.002	0.00	n.a.	MB
20	44.44		0.020	0.001	0.00	n.a.	BMB
Total:			187.181	209.448	100.00	0.000	

1024C

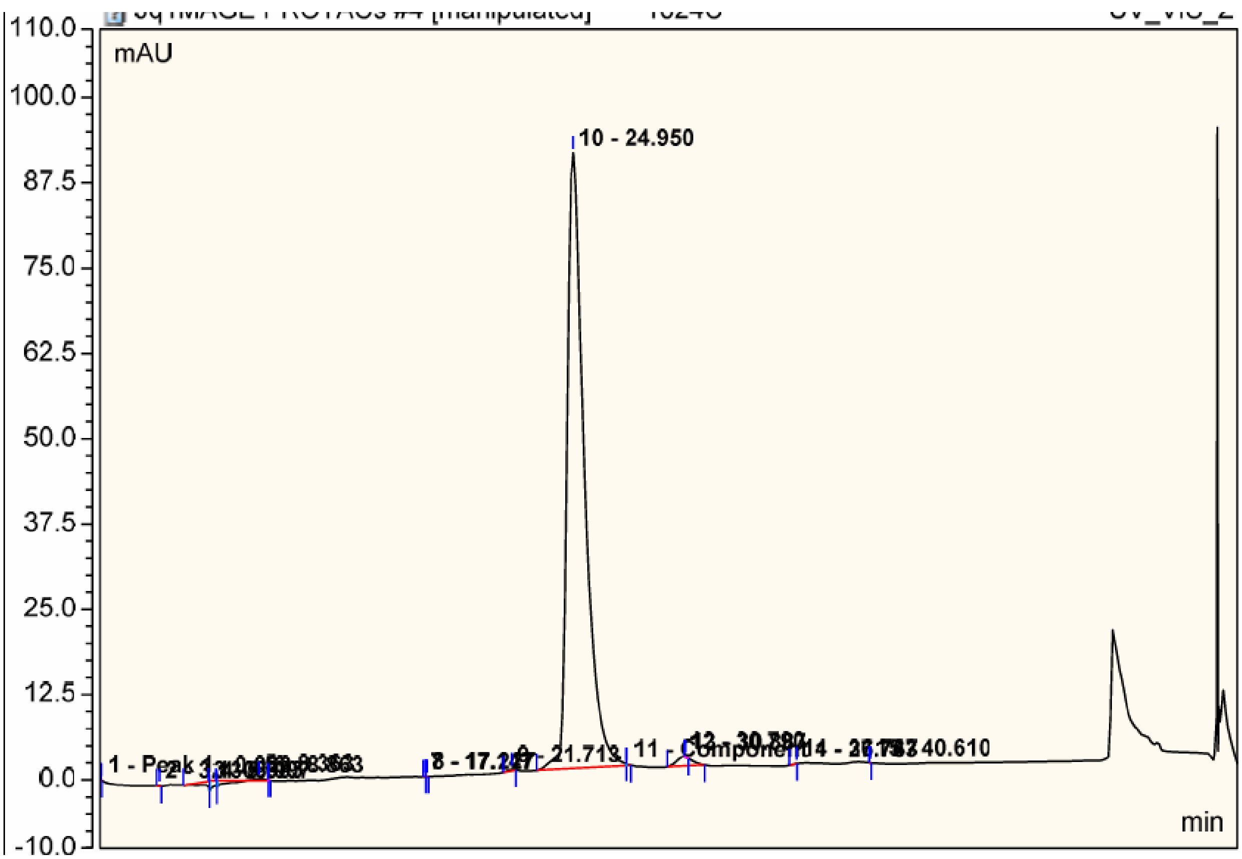

No.	Ret.Time min	Peak Name	Height mAU	Area mAU*min	Rel.Area %	Amount micromol	Type
1	0.06	Peak 1	0.057	0.002	0.00	n.a.	BMB
2	3.13		0.093	0.010	0.01	n.a.	BMB
3	5.68		0.517	0.413	0.42	n.a.	BM *
4	6.10		0.703	0.394	0.40	n.a.	M *
5	8.36		0.294	0.358	0.37	n.a.	MB
6	8.86		0.010	0.000	0.00	n.a.	Rd
7	17.15		0.037	0.003	0.00	n.a.	BM
8	17.23		0.032	0.003	0.00	n.a.	MB
9	21.71		0.167	0.071	0.07	n.a.	BMB
10	24.95		90.223	95.551	97.36	n.a.	BM
11	27.76	Component 1	0.232	0.031	0.03	n.a.	MB
12	30.79		1.398	1.285	1.31	n.a.	BMB
13	30.89		0.053	0.008	0.01	n.a.	Rd
14	36.75		0.017	0.006	0.01	n.a.	BMB
15	40.61		0.019	0.001	0.00	n.a.	BMB
Total:			93.851	98.137	100.00	0.000	

1024B

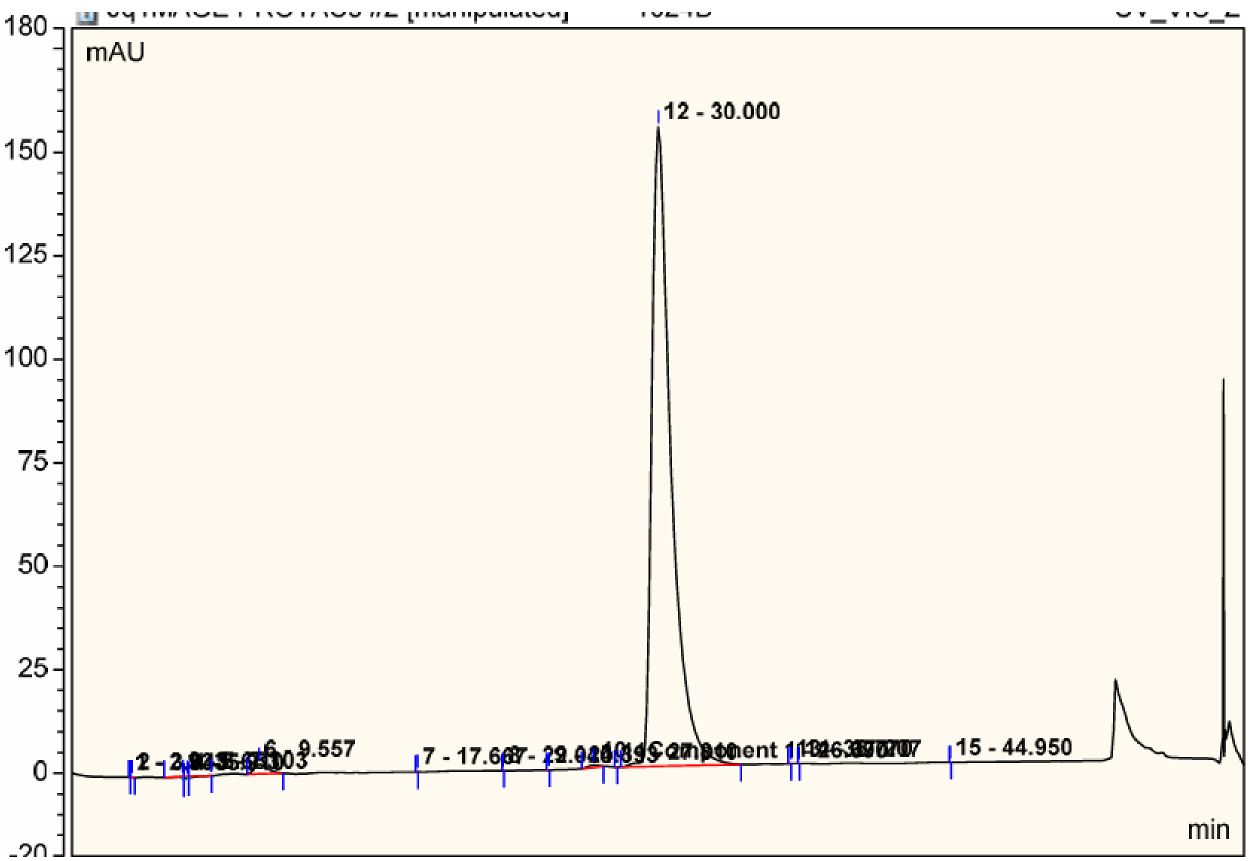

No.	Ret.Time min	Peak Name	Height mAU	Area mAU*min	Rel.Area %	Amount micromol	Type
1	2.93		0.027	0.001	0.00	n.a.	BMb
2	3.04		0.064	0.013	0.01	n.a.	bMB
3	5.65		0.025	0.066	0.04	n.a.	BM *
4	5.91		0.285	0.112	0.06	n.a.	M *
5	7.10		0.002	0.079	0.04	n.a.	MB
6	9.56		2.328	1.880	1.04	n.a.	BMB
7	17.67		0.020	0.001	0.00	n.a.	BMB
8	22.04		0.021	0.001	0.00	n.a.	BMB
9	24.39		0.019	0.001	0.00	n.a.	BMB
10	26.69	Component 1	0.468	0.298	0.16	n.a.	BMB
11	27.81		0.015	0.001	0.00	n.a.	BMB
12	30.00		154.402	178.751	98.64	n.a.	BMB
13	36.77		0.014	0.001	0.00	n.a.	BMB
14	37.21		0.022	0.001	0.00	n.a.	BMB
15	44.95		0.021	0.001	0.00	n.a.	BMB
Total:			157.732	181.209	100.00	0.000	

1024E

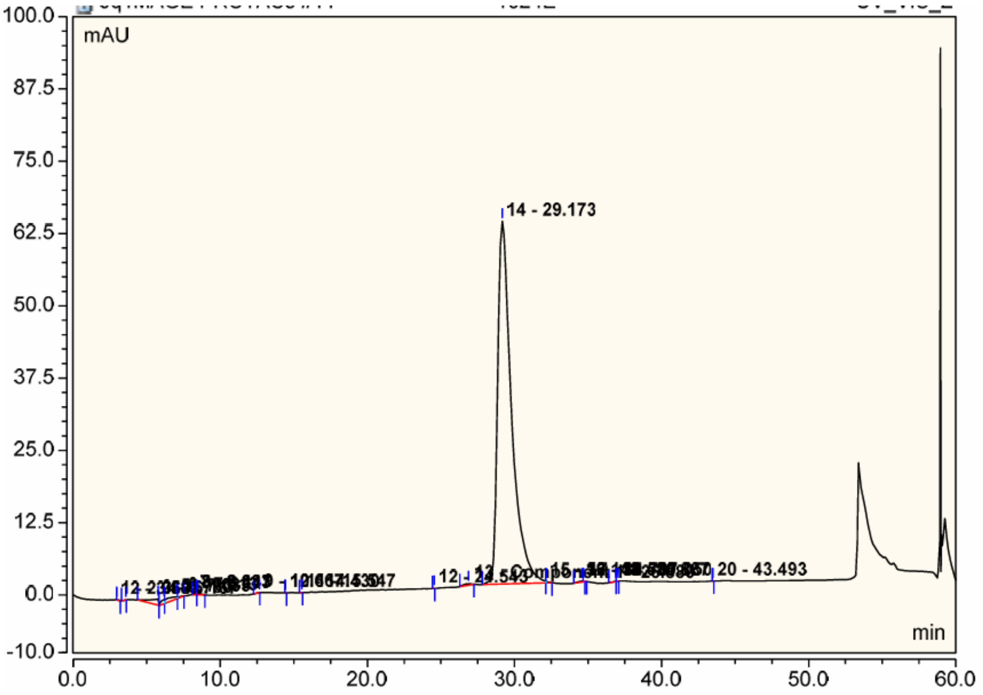

No.	Ret.Time min	Peak Name	Height mAU	Area mAU*min	Rel.Area %	Amount micromol	Type
1	2.96		0.009	0.013	0.02	n.a.	BMB
2	3.58		0.016	0.005	0.01	n.a.	BMB
3	5.78		1.107	0.721	1.02	n.a.	BMB
4	6.17		0.759	0.210	0.30	n.a.	BM
5	7.03		0.308	0.443	0.63	n.a.	M
6	7.51		0.023	0.053	0.07	n.a.	MB
7	8.38		0.012	0.000	0.00	n.a.	Ru
8	8.45		0.264	0.120	0.17	n.a.	BMB
9	12.67		0.023	0.013	0.02	n.a.	BMB
10	14.43		0.018	0.001	0.00	n.a.	BMB
11	15.55		0.022	0.003	0.00	n.a.	BMB
12	24.54		0.016	0.002	0.00	n.a.	BMB
13	26.88	Component 1	0.235	0.162	0.23	n.a.	BMB
14	29.17		62.740	68.471	97.27	n.a.	BM
15	32.14		0.276	0.054	0.08	n.a.	MB
16	34.58		0.199	0.100	0.14	n.a.	BMB
17	34.71		0.016	0.001	0.00	n.a.	Rd
18	36.89		0.050	0.015	0.02	n.a.	BM
19	37.05		0.028	0.005	0.01	n.a.	MB
20	43.49		0.023	0.001	0.00	n.a.	BMB
Total:			66.143	70.394	100.00	0.000	

1024D

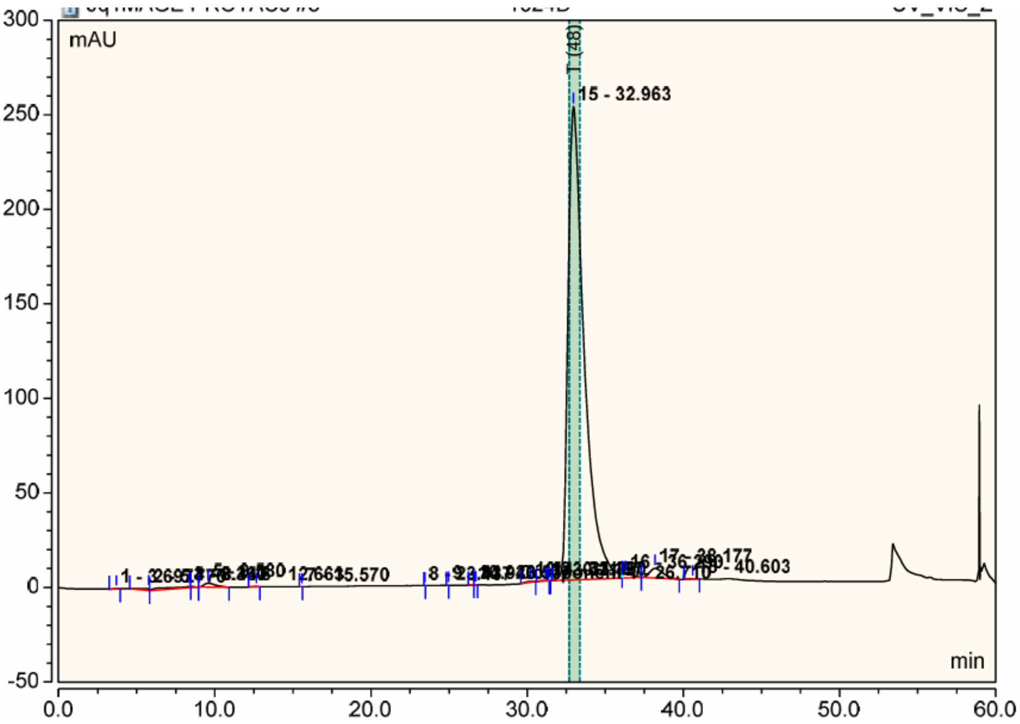

No.	Ret.Time min	Peak Name	Height mAU	Area mAU*min	Rel.Area %	Amount micromol	Type
1	3.70		0.122	0.046	0.01	n.a.	BMB
2	5.77		1.148	0.682	0.22	n.a.	BMB
3	8.36		0.747	2.078	0.67	n.a.	BMb
4	8.45		0.010	0.000	0.00	n.a.	Rd
5	9.58		2.119	1.794	0.58	n.a.	bMB
6	12.66		0.120	0.058	0.02	n.a.	BMB
7	15.57		0.017	0.002	0.00	n.a.	BMB
8	23.44		0.023	0.001	0.00	n.a.	BMB
9	24.94		0.015	0.002	0.00	n.a.	BMB
10	26.58		0.076	0.019	0.01	n.a.	BM
11	26.71	Component 1	0.065	0.010	0.00	n.a.	MB
12	30.22		0.412	0.272	0.09	n.a.	BMB
13	31.20		0.214	0.140	0.05	n.a.	BMB
14	31.36		0.013	0.000	0.00	n.a.	Rd
15	32.96		250.490	298.299	96.14	n.a.	BM
16	36.29		2.050	1.550	0.50	n.a.	Mb
17	38.18		5.191	5.155	1.66	n.a.	bMB
18	40.60		0.257	0.160	0.05	n.a.	BMB
Total:			263.090	310.269	100.00	0.000	

HRMS/LCMS

105B

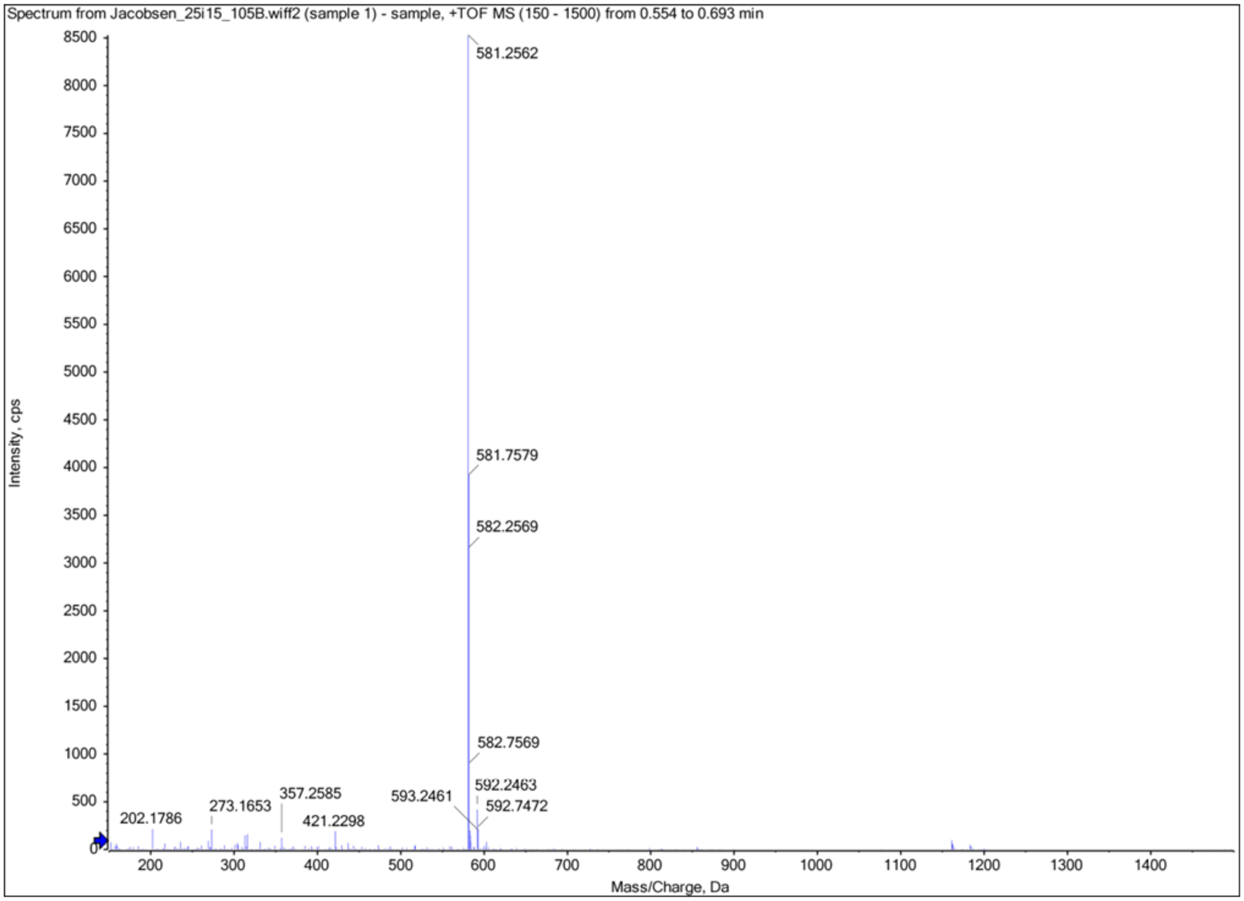

SJ1008066

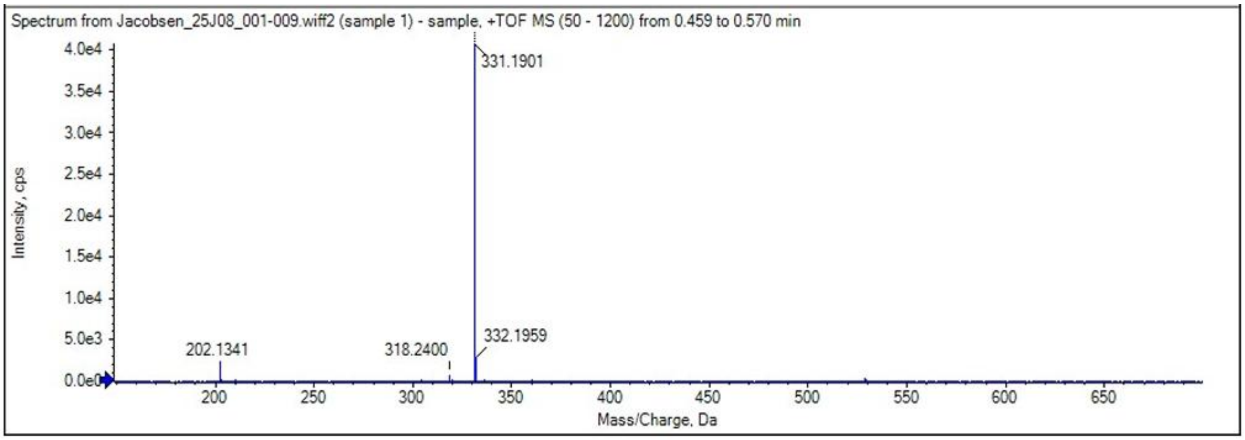

913B

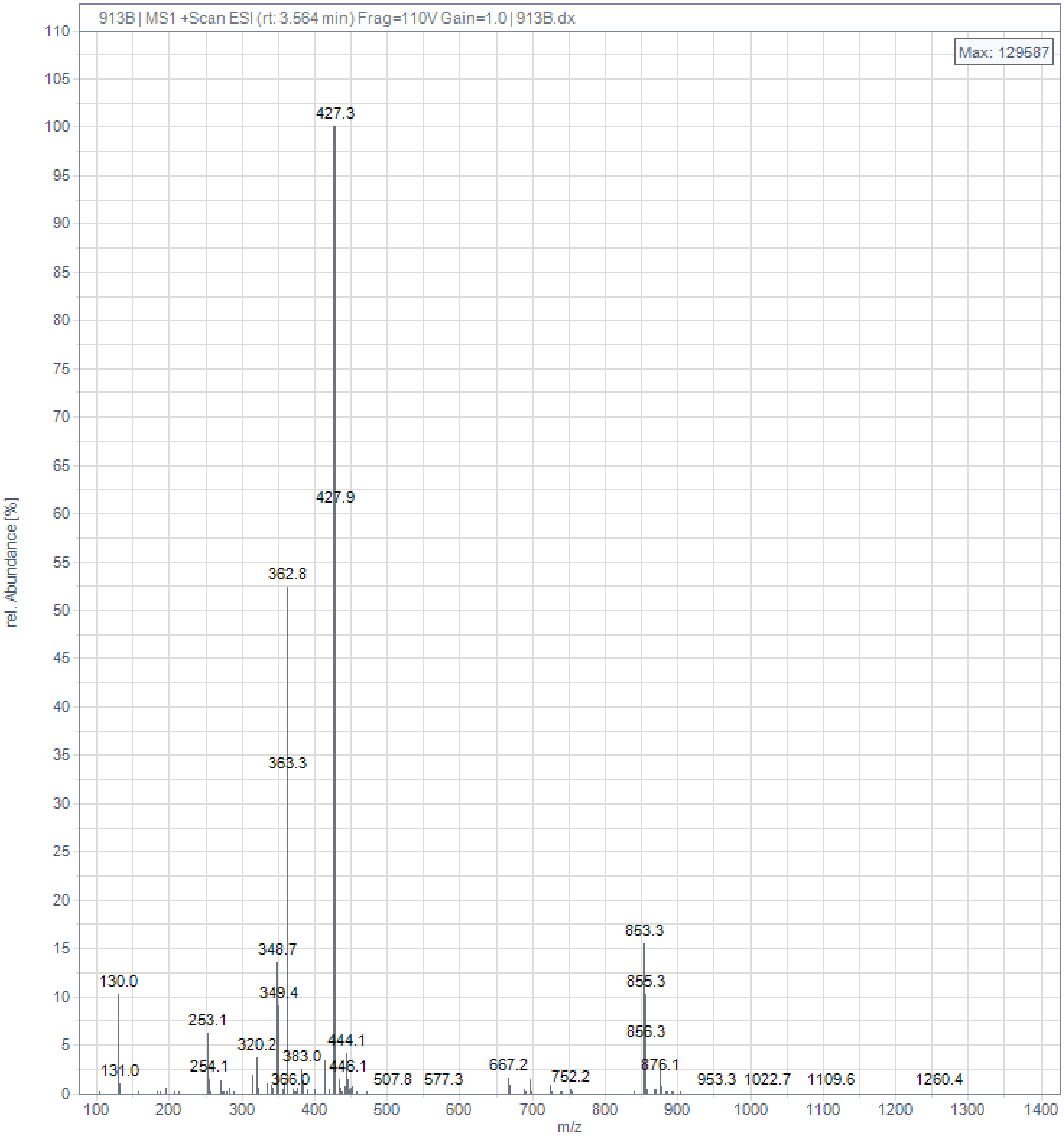

913B | MS1 +Scan ESI (rt: 3.564 min) Frag=110V Gain=1.0 | 913B.dx
Max: 129587
rel. Abundance [%]
m/z
427.3
427.9
362.8
363.3
348.7
349.4
130.0
131.0
253.1
254.1
320.2
383.0
366.0
444.1
446.1
507.8
577.3
667.2
752.2
853.3
855.3
856.3
876.1
953.3
1022.7
1109.6
1260.4

908B

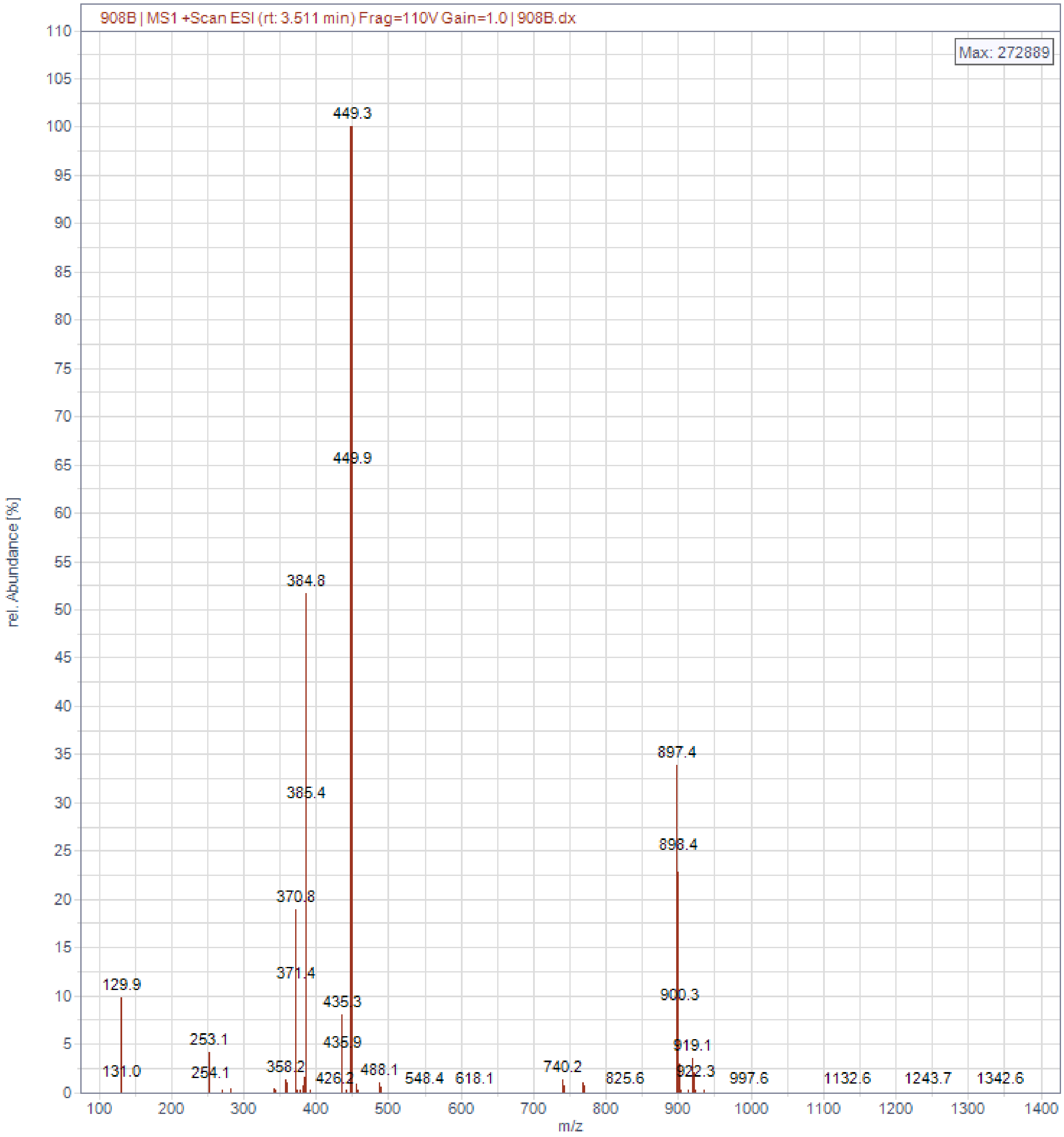

908B | MS1 +Scan ESI (rt: 3.511 min) Frag=110V Gain=1.0 | 908B.dx
Max: 272889
rel. Abundance [%]
m/z
449.3
449.9
384.8
385.4
370.8
371.4
129.9
131.0
253.1
254.1
358.2
426.2
435.3
435.9
488.1
548.4
618.1
740.2
825.6
897.4
898.4
900.3
919.1
922.3
997.6
1132.6
1243.7
1342.6

913C

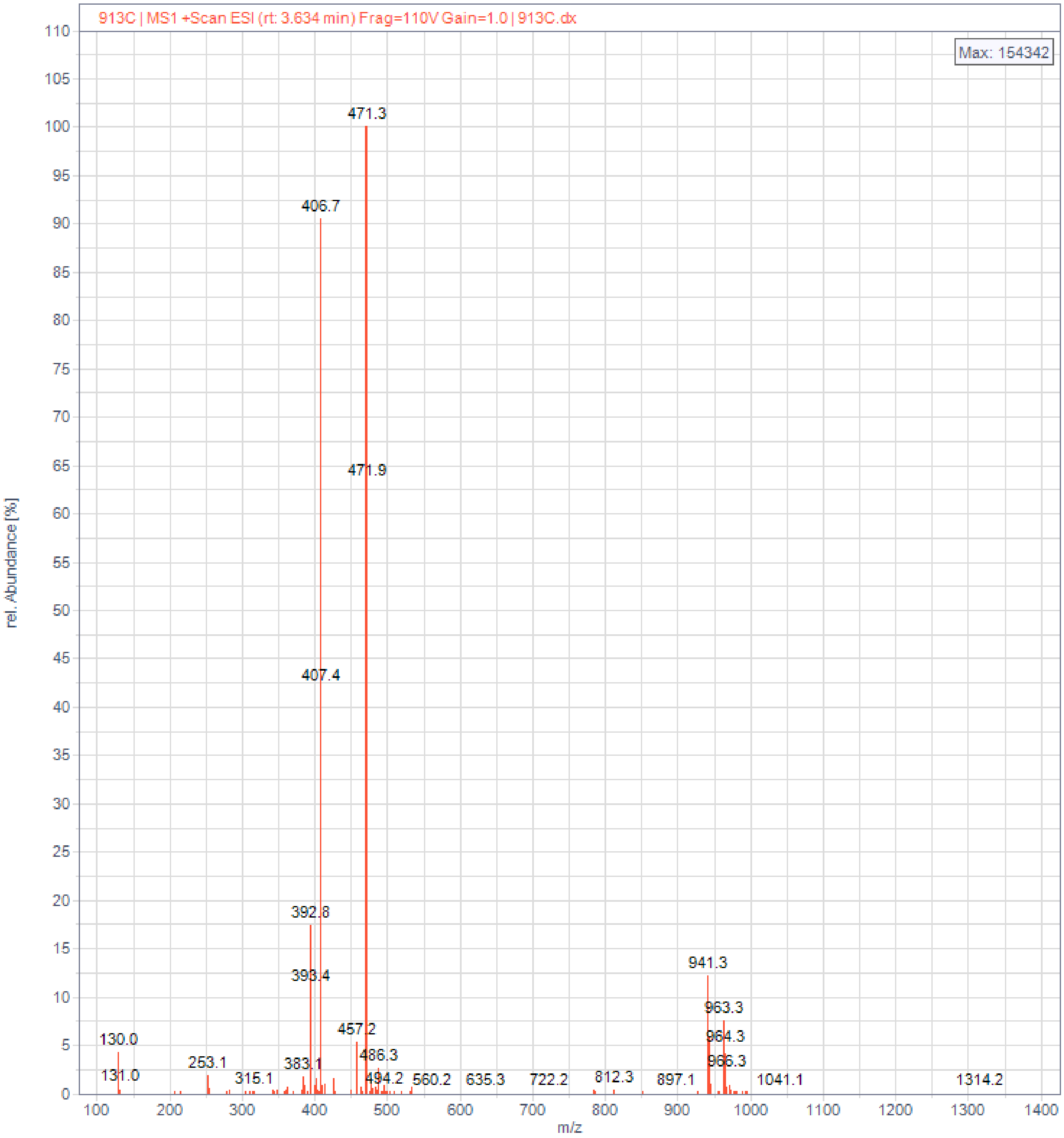
913C | MS1 +Scan ESI (rt: 3.634 min) Frag=110V Gain=1.0 | 913C.dx
Max: 154342
471.3
406.7
471.9
407.4
392.8
393.4
941.3
963.3
964.3
966.3
457.2
486.3
494.2
130.0
131.0
253.1
315.1
383.1
560.2
635.3
722.2
812.3
897.1
1041.1
1314.2
rel. Abundance [%]
m/z

1024F

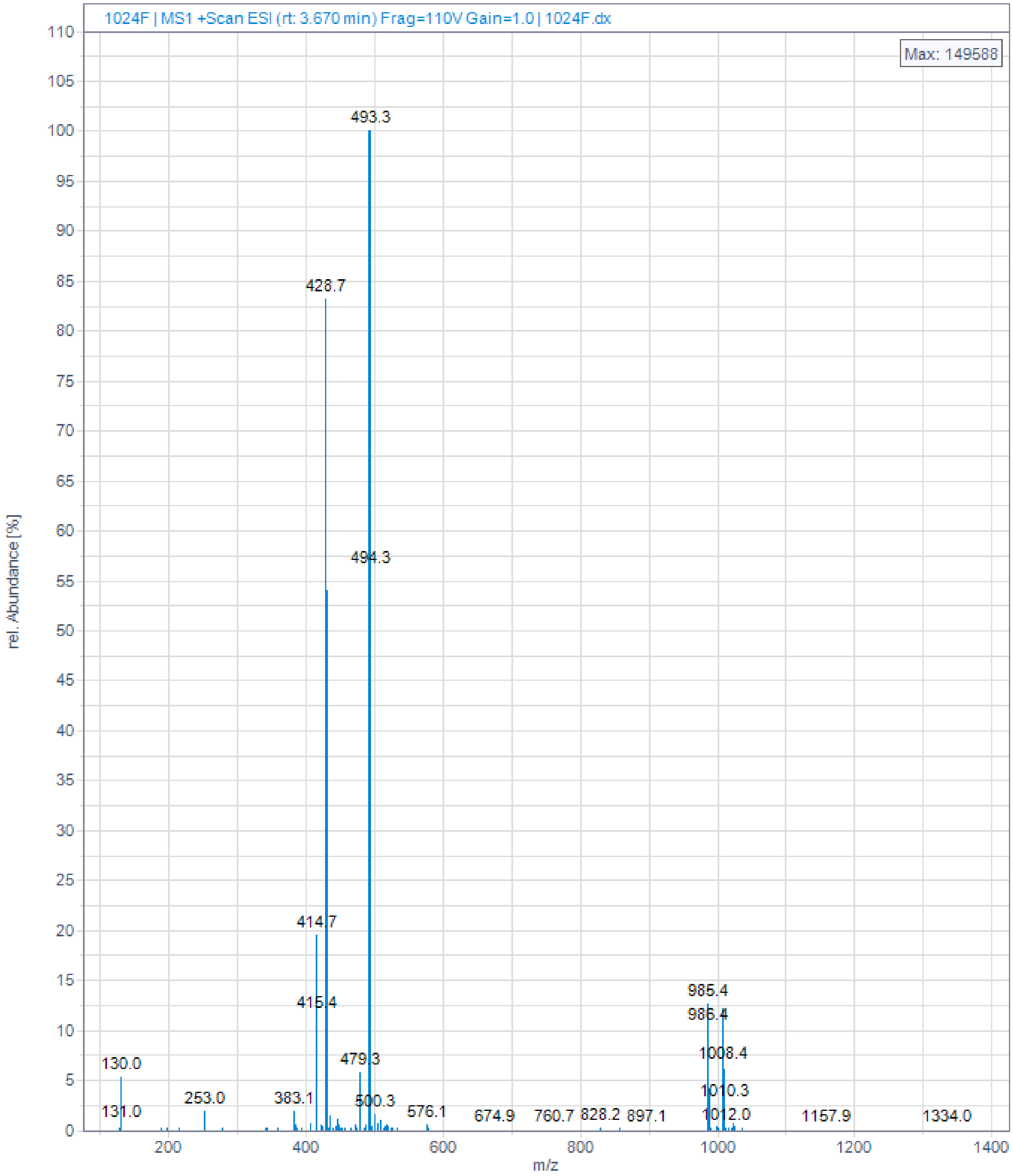

1024F | MS1 +Scan ESI (rt: 3.670 min) Frag=110V Gain=1.0 | 1024F.dx
Max: 149588
493.3
428.7
494.3
414.7
415.4
985.4
986.4
1008.4
1010.3
1012.0
130.0
131.0
253.0
383.1
479.3
500.3
576.1
674.9
760.7
828.2
897.1
1157.9
1334.0
rel. Abundance [%]
m/z

105D

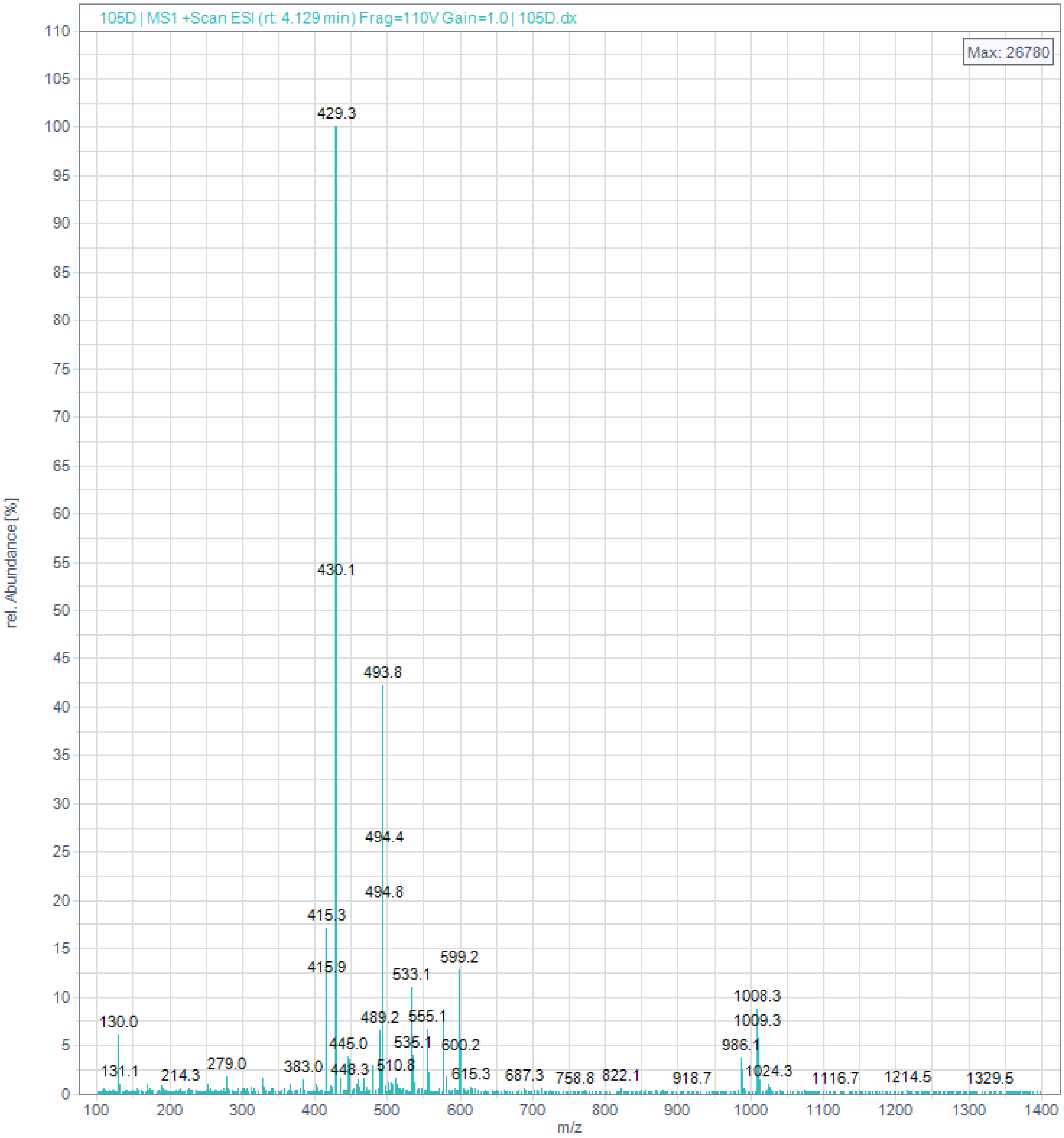
105D | MS1 +Scan ESI (rt: 4.129 min) Frag=110V Gain=1.0 | 105D.dx
Max: 26780
rel. Abundance [%]
m/z
429.3
430.1
493.8
494.4
494.8
415.3
415.9
599.2
533.1
1008.3
1009.3
986.1
130.0
489.2
555.1
445.0
535.1
600.2
131.1
214.3
279.0
383.0
448.3
510.8
615.3
687.3
758.8
822.1
918.7
1024.3
1116.7
1214.5
1329.5

913D

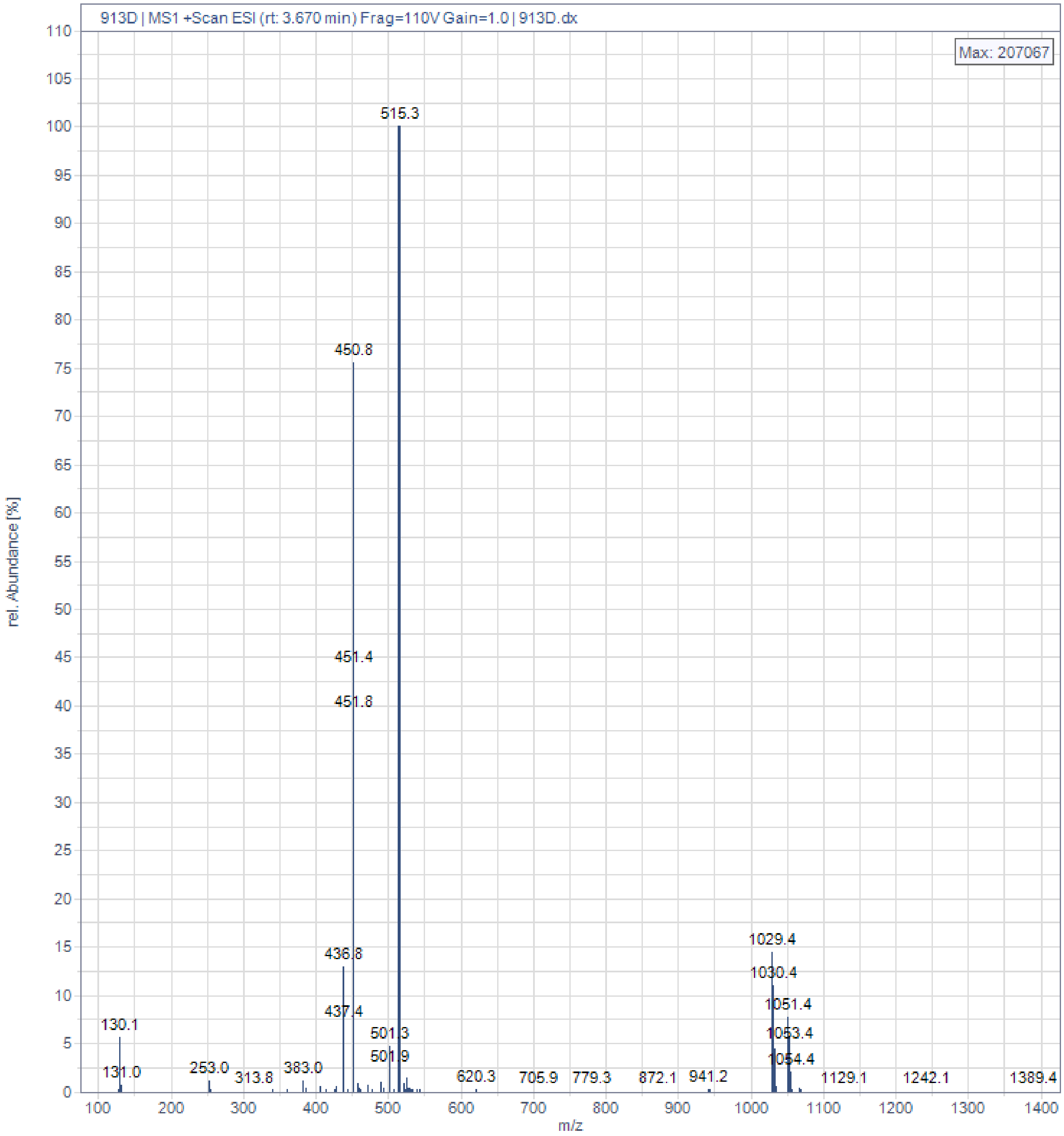
913D | MS1 +Scan ESI (rt: 3.670 min) Frag=110V Gain=1.0 | 913D.dx
Max: 207067
rel. Abundance [%]
m/z
515.3
450.8
451.4
451.8
436.8
437.4
501.3
501.9
130.1
131.0
253.0
313.8
383.0
620.3
705.9
779.3
872.1
941.2
1029.4
1030.4
1051.4
1053.4
1054.4
1129.1
1242.1
1389.4

105A

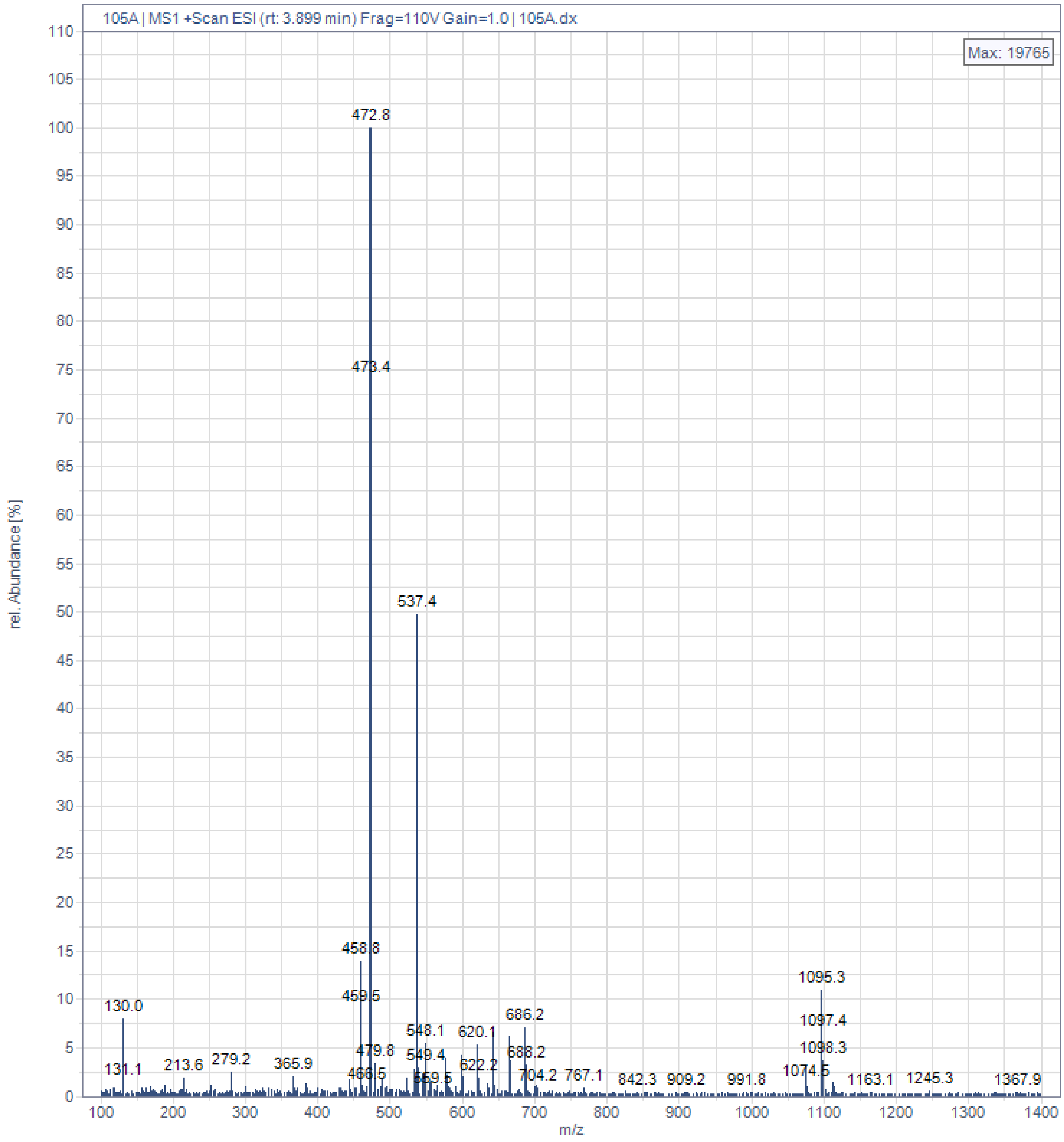

105A | MS1 +Scan ESI (rt: 3.899 min) Frag=110V Gain=1.0 | 105A.dx
Max: 19765
rel. Abundance [%]
m/z
472.8
473.4
537.4
458.8
459.5
130.0
131.1
213.6
279.2
365.9
466.5
479.8
548.1
549.4
559.5
620.1
622.2
686.2
688.2
704.2
767.1
842.3
909.2
991.8
1074.5
1095.3
1097.4
1098.3
1163.1
1245.3
1367.9

105E

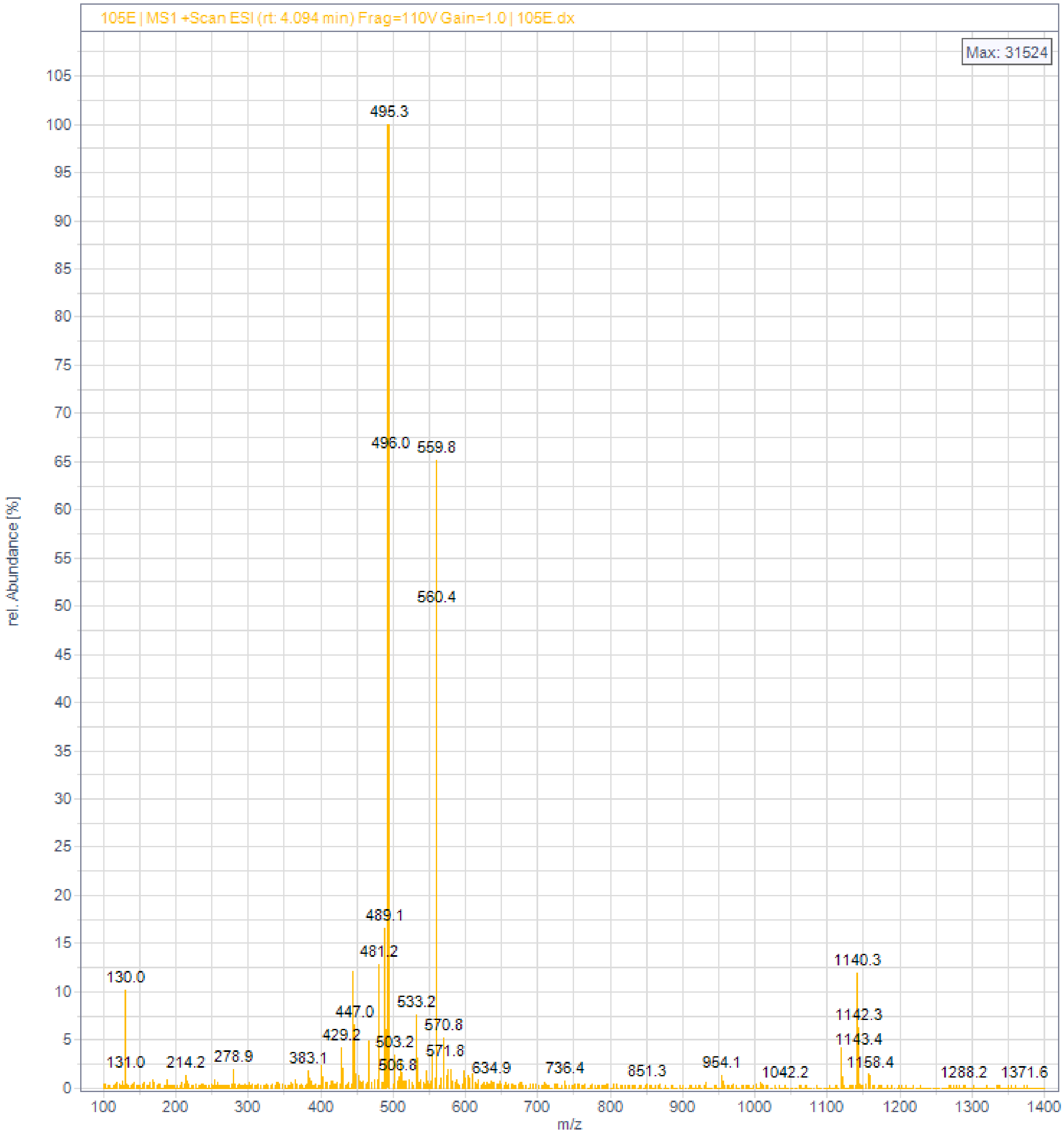

105E | MS1 +Scan ESI (rt: 4.094 min) Frag=110V Gain=1.0 | 105E.dx
Max: 31524
rel. Abundance [%]
m/z
495.3
496.0
559.8
560.4
489.1
481.2
130.0
1140.3
533.2
447.0
570.8
429.2
503.2
571.8
506.8
131.0
214.2
278.9
383.1
634.9
736.4
851.3
954.1
1042.2
1142.3
1143.4
1158.4
1288.2
1371.6

105C

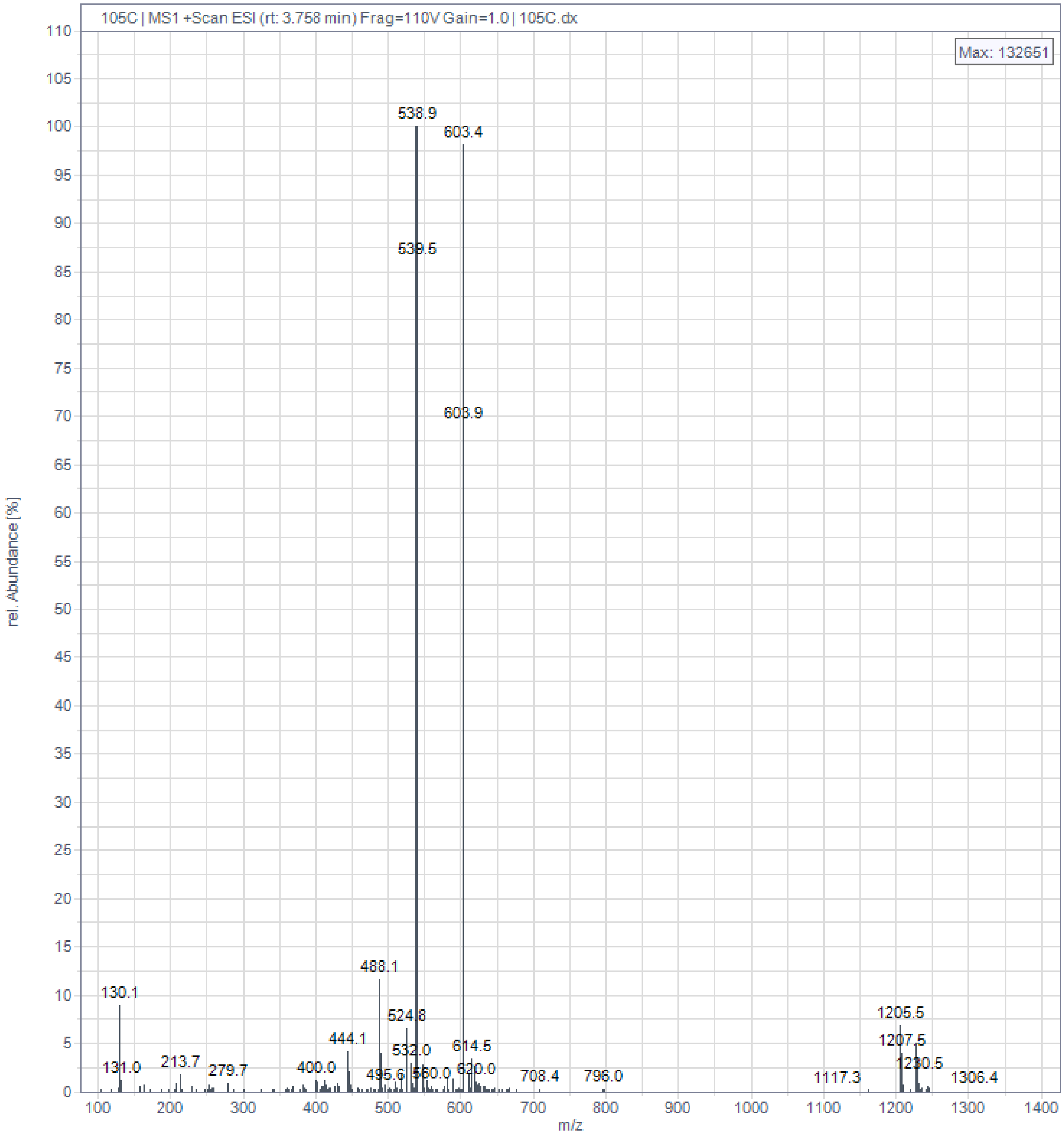
105C | MS1 +Scan ESI (rt: 3.758 min) Frag=110V Gain=1.0 | 105C.dx
Max: 132651
rel. Abundance [%]
m/z
538.9
603.4
539.5
603.9
488.1
130.1
524.8
1205.5
444.1
532.0
614.5
1207.5
131.0
213.7
279.7
400.0
495.6
550.0
620.0
1230.5
708.4
796.0
1117.3
1306.4

1024C

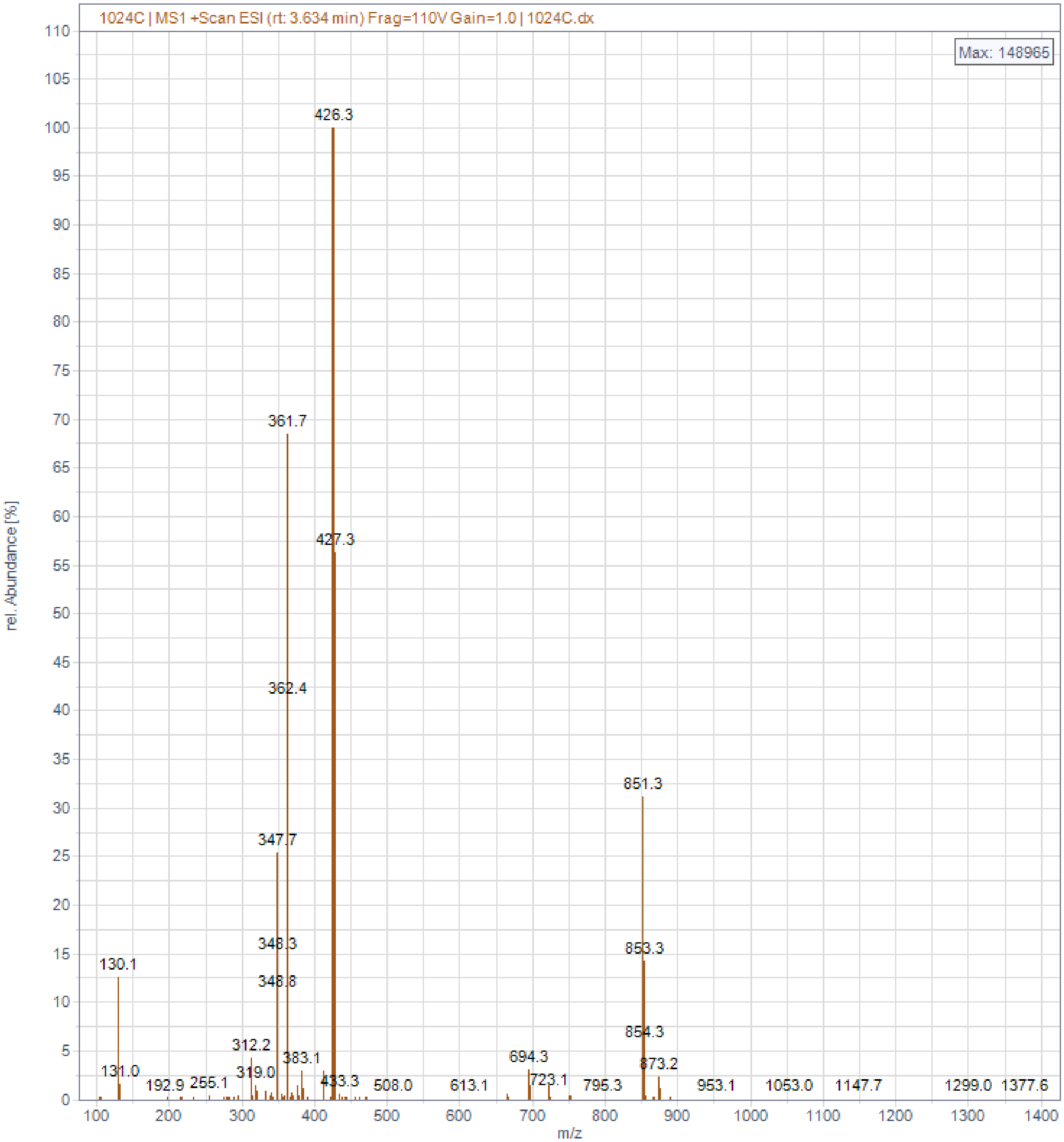

1024C | MS1 +Scan ESI (rt: 3.634 min) Frag=110V Gain=1.0 | 1024C.dx
Max: 148965
rel. Abundance [%]
m/z
426.3
361.7
427.3
362.4
851.3
347.7
348.3
348.8
853.3
130.1
854.3
312.2
383.1
694.3
873.2
131.0
319.0
433.3
723.1
192.9
255.1
508.0
613.1
795.3
953.1
1053.0
1147.7
1299.0
1377.6

1024B

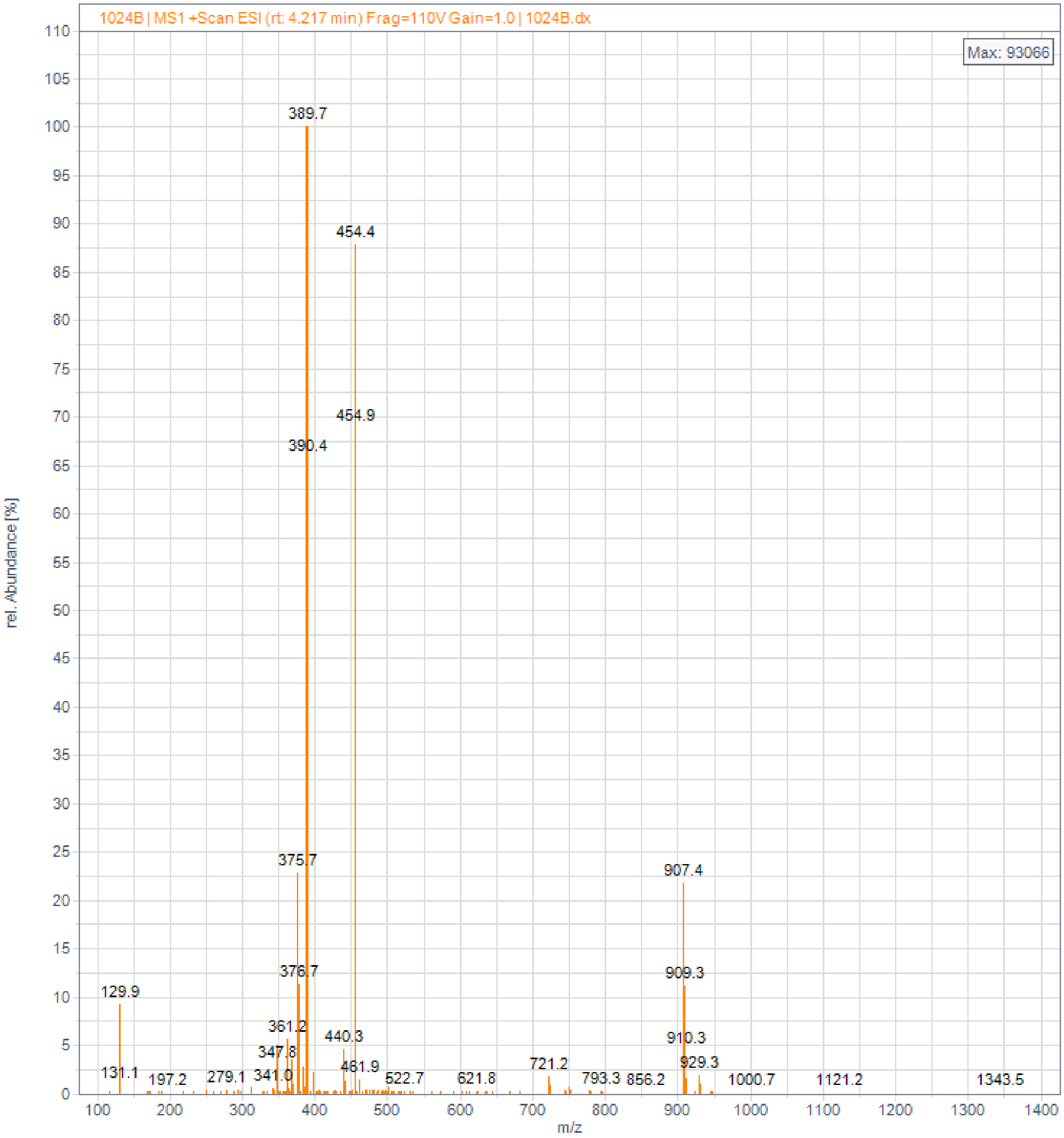

1024B | MS1 +Scan ESI (rt: 4.217 min) Frag=110V Gain=1.0 | 1024B.dx
Max: 93066
rel. Abundance [%]
m/z
389.7
454.4
454.9
390.4
375.7
907.4
376.7
909.3
129.9
361.2
440.3
910.3
347.8
461.9
721.2
929.3
131.1
197.2
279.1
341.0
522.7
621.8
793.3
856.2
1000.7
1121.2
1343.5

1024E

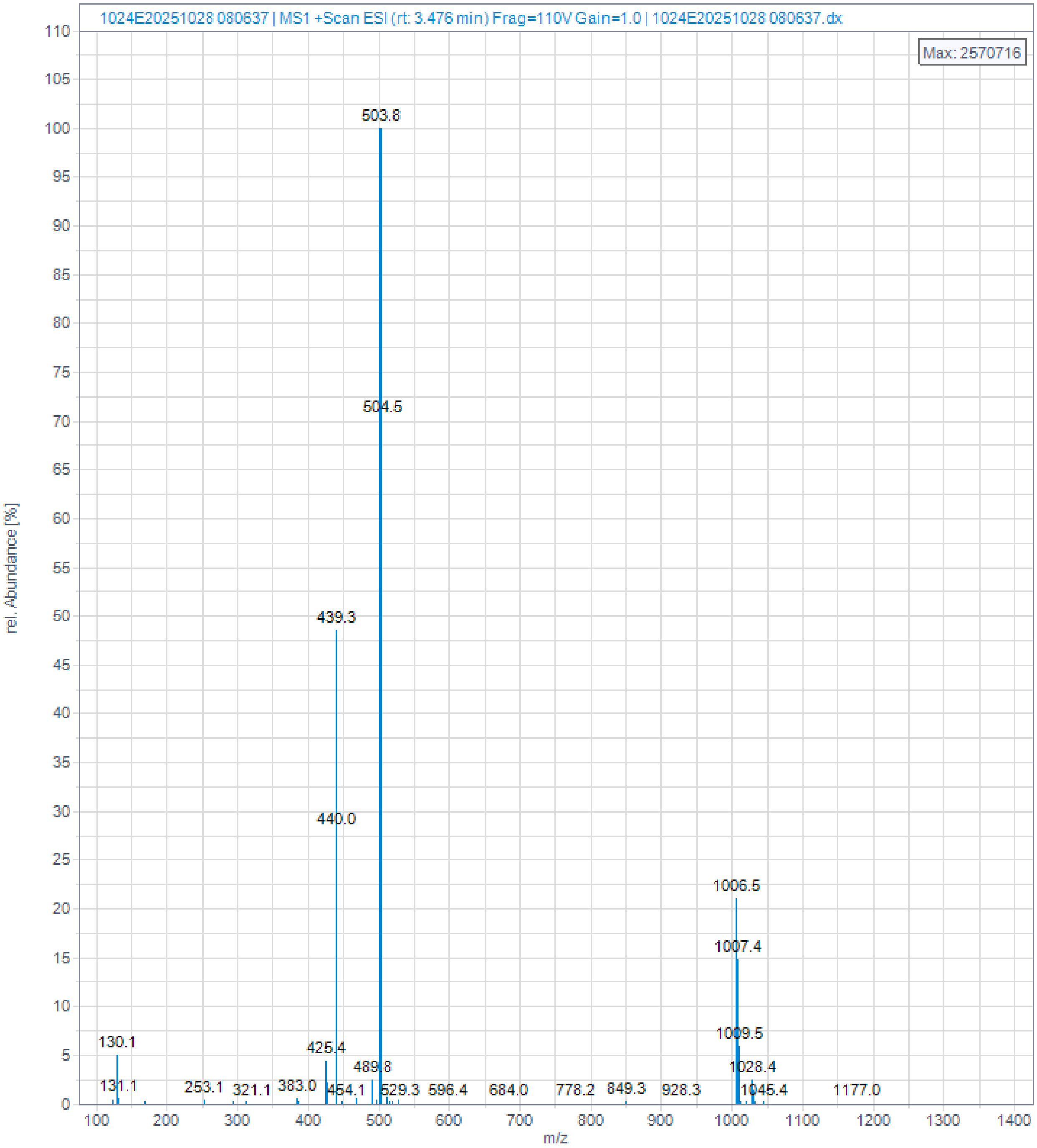

1024E20251028 080637 | MS1 +Scan ESI (rt: 3.476 min) Frag=110V Gain=1.0 | 1024E20251028 080637.dx
Max: 2570716
rel. Abundance [%]
m/z
503.8
504.5
439.3
440.0
1006.5
1007.4
1009.5
1028.4
1045.4
130.1
131.1
253.1
321.1
383.0
425.4
454.1
489.8
529.3
596.4
684.0
778.2
849.3
928.3
1177.0

1024D

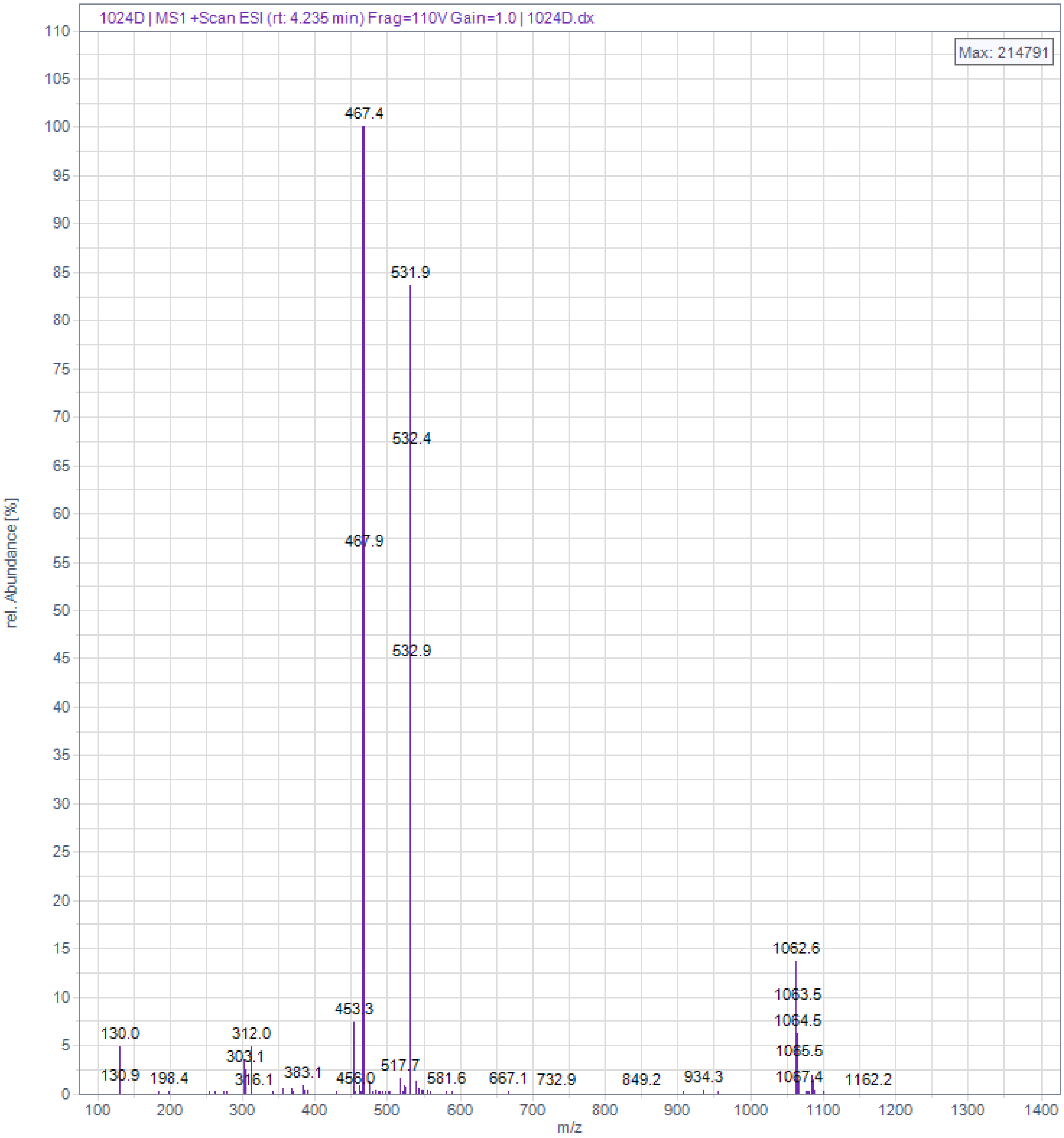
1024D | MS1 +Scan ESI (rt: 4.235 min) Frag=110V Gain=1.0 | 1024D.dx
Max: 214791
rel. Abundance [%]
m/z
467.4
531.9
532.4
467.9
532.9
1062.6
1063.5
1064.5
1065.5
1067.4
453.3
130.0
312.0
303.1
130.9
198.4
316.1
383.1
456.0
517.7
581.6
667.1
732.9
849.2
934.3
1162.2
